# Computational analysis of substituent effects on proton affinity and gas-phase basicity of TEMPO derivatives and their hydrogen bonding interactions with water molecules

**DOI:** 10.1038/s41598-024-58582-x

**Published:** 2024-04-10

**Authors:** Abolfazl Shiroudi, Maciej Śmiechowski, Jacek Czub, Mohamed A. Abdel-Rahman

**Affiliations:** 1 Department of Physical Chemistry, Gdańsk University of Technology, Narutowicza 11/12, 80-233 Gdańsk, Poland; 2 BioTechMed Center, Gdańsk University of Technology, 80-233 Gdańsk, Poland; 3 Department of Chemistry, Faculty of Science, Suez University, P.O. Box: 43221, Suez, Egypt

**Keywords:** TEMPO, Proton affinity, Basicity, NBO, AIM, Stability, Physical chemistry, Theoretical chemistry

## Abstract

The study investigates the molecular structure of 2,2,6,6-tetramethylpiperidine-1-oxyl (TEMPO) and its derivatives in the gas phase using B3LYP and M06-2X functional methods. Intermolecular interactions are analyzed using natural bond orbital (NBO) and atoms in molecules (AIM) techniques. NO_2_-substituted TEMPO displays high reactivity, less stability, and softer properties. The study reveals that the stability of TEMPO derivatives is mainly influenced by LP(e) → *σ*^∗^ electronic delocalization effects, with the highest stabilization observed on the oxygen atom of the nitroxide moiety. This work also considers electron density, atomic charges, and energetic and thermodynamic properties of the studied NO radicals, and their relative stability. The proton affinity and gas-phase basicity of the studied compounds were computed at *T* = 298 K for O-protonation and N-protonation, respectively. The studied DFT method calculations show that O-protonation is more stable than N-protonation, with an energy difference of 16.64–20.77 kcal/mol (22.80–25.68 kcal/mol) at the B3LYP (M06-2X) method. The AIM analysis reveals that the N–O…H interaction in H_2_O complexes has the most favorable hydrogen bond energy computed at bond critical points (3, − 1), and the planar configurations of TEMPO derivatives exhibit the highest E_HB_ values. This indicates stronger hydrogen bonding interactions between the N–O group and water molecules.

## Introduction

Nitroxides are stable organic free radicals with an N–O group containing an unpaired electron^1^, and they have diverse applications in chemistry, biology, and biochemistry^1–5^. Nitroxides play vital roles in the nervous system^6^ and serve various functions in the immune system to counteract infectious and autoimmune diseases^7^. Additionally, nitroxide radicals serve as building blocks for organic magnetic materials, propagating ferromagnetic interactions within supramolecular assemblies^8,9^.

Amphiphilic nitroxide (NO^·^) radicals, like 2,2,6,6-tetramethylpiperidine-1-oxyl (TEMPO) and its derivatives, are highly suitable for studying macromolecular systems, particularly nanoscale inhomogeneities in polymers, due to their ability to probe different regions based on size and polarity^10,11^. The size and amphiphilic character of these can be easily adjusted by changing the structural unit in the 4-position of the piperidine ring, allowing for the use of a wide range of structurally diverse spin probes for several applications^12^. Nitroxides, including TEMPO, are crucial in controlled radical polymerization reactions^13–16^ and are often used in magnetic resonance hyperpolarization methods, such as dynamic nuclear polarization, to enhance the intensity of NMR signals^17–19^. TEMPO radical is well-known for its aqueous stability, and TEMPO derivatization results in even greater stability and water solubility^20^.

Alkorta and coworkers^21^ conducted a comprehensive study involving 1125 structures of nitroxide free radicals and hydrogen-bonded complexes. They aimed to perform both qualitative and quantitative analyses of hydrogen bonds using the second-order Møller–Plesset perturbation theory (MP2) method^22^. Specifically focusing on TEMPO and related compounds, they calculated frequencies using the B3LYP method^23,24^ along with the 6–311++ G(d,p) basis set^25^.

Ismail et al.^26^ investigated the intermolecular hydrogen bonding of a group of 23 nitroxide radicals, with TEMPO as a primary focus. They employed the UM06L/6–311++ G(d,p) level of theory for their analysis. Their study involved a comprehensive and quantitative theoretical examination of the intermolecular NO…H hydrogen bonds formed by NO radicals with HF, H_2_O, and CH_4_ acting as electron acceptors. In the case of all considered compounds especially HF complexes, the hydrogen bonding nature is expected to demonstrate partially closed-shell and shared-type interactions with electron-donating groups, while predominantly exhibiting closed-shell type interactions in systems with electron-withdrawing groups.

Furthermore, Brás et al.^27^ investigated the hydration of TEMPO and identified three types of H-bond donating coordination of the first water molecule to the negative end of the NO bond. These include embedding by the two geminal methyl groups on one half of the puckered ring (referred to as planar), alignment with the two axial methyl groups on one face of the ring (referred to as top), or pointing to the opposite open side of the ring framed by the two equatorial methyl groups (referred to as orthogonal). The relative abundance of 1:1 planar, 1:1 top, and 1:1 orthogonal monohydrate complexes serves as a sensitive probe of the difference between the NO bonds towards H-bonding. The calculations predict that all three structures are energetically closely spaced minima for the TEMPO hydrate.

The computation of thermodynamic properties for molecules, ions, and radicals has long been a fundamental application of quantum chemistry calculations. This approach is particularly effective for gas-phase species as they can be treated as independent, free molecules. Proton affinity (PA) holds a special place among the various molecular properties that can be calculated. Both the studied molecule and its protonated form (cation) are closed-shell species with an equal number of electrons. This symmetry helps offset some inherent errors in computational methods, resulting in higher accuracy in PA values compared to heats of formation or bond dissociation energies, which require calculations for open-shell systems^28^. Proton transfer reactions are crucial in various chemical, biological, and environmental systems^29,30^ as they help determine intrinsic properties like proton affinity and gas-phase basicity (GB), which aid in investigating proton donor and acceptor positions. These values are specific to specific sites within a molecule and provide insights into molecular structures, stability, and reactivity of organic molecules^31^.

Experimental determination of the PAs of molecules is often challenging^32^. Typically, relative PAs are determined using mass spectrometry [in reaction: MH^+^ + B ⇌ M + BH^+^]^33,34^, while absolute PAs can be obtained from ionization thresholds [in reaction: MH → MH^+^ + e^−^]^33^. However, not all MH molecules exist in sufficient quantities for this approach. Various experimental techniques, including ion mobility spectrometry^35^, mass spectrometry^36,37^, calorimetry, kinetic methods, and ion cyclotron resonance methods, have been used for PA and GB values^35,38–41^. However, accurate experimental measurements of nitroxide radicals and their protonated forms are challenging, leading to the development of computational methods as an alternative^42–48^. Compute proton affinity and gas-phase basicity values using computational methods, which require detailed information about the structures, energies, and vibrational frequencies of both nitroxide radicals and their protonated forms.

Density functional theory (DFT) methods^49,50^ with well-justified basis sets have demonstrated their ability to accurately predict the energetic and thermodynamic properties of chemical systems^45,46^. This research focused on examining the geometry, electronic properties, and reactivity descriptors of TEMPO derivatives through DFT calculations, specifically examining the protonation of nitrogen and oxygen atoms in TEMPO and its four different para-substituted derivatives. These substituents include electron-donating (ED) groups (–CH_3_, –NH_2_) and electron-withdrawing (EW) substituents (–CHO, –NO_2_). The choice of the ED and EW substituent groups in the study of PA and GB of TEMPO and its derivatives is motivated by the desire to gain fundamental insights into their electronic structure, stability, reactivity, and potential applications across various scientific disciplines. The study used the B3LYP^23,24^ and M06-2X^51,52^ levels of theory to conduct protonation reactions at *T* = 298 K to manipulate the acidity and basicity of chemical compounds. It utilized the computed energies, frequencies, and structural parameters of both TEMPO derivatives and their protonated forms to determine PA and GB values. To comprehend the frontier molecular orbital (FMO) and natural bond orbital (NBO) analyses at the same theoretical level, we employed the NBO 6 program^53^, which is included in the Gaussian 16 package^54^. In this work, along with the investigation of protonation at oxygen and nitrogen atoms in the considered TEMPO derivatives and the calculation of their proton affinity and gas-phase basicity, we conduct a hydration study of these molecules. Based on the scientific work conducted by Brás et al.^27^, we investigate three types of hydrogen bond donating coordination of the first water to the negative end of the NO bond. Specifically, we analyze topological features such as electron density (ρ), the Laplacian of electron density (∇^2^ρ), and hydrogen bonding interactions at the bond critical points (BCPs). This analysis provides valuable insights into the nature and strength of chemical bonding interactions between water molecules and the TEMPO derivatives under study.

## Computational method

DFT calculations, involving geometry optimizations and frequency calculations of TEMPO derivatives and their protonated forms, were performed using two hybrid functionals, including the B3LYP, and M06-2X functionals, in conjunction with the split valence 6–311++ G(d,p) basis set^25^ that incorporates sufficient polarization and diffuse functions for both hydrogen and non-hydrogen atoms. All calculations in this study were performed using the Gaussian 16 package. Molecular structures were visually analyzed using the ChemCraft program^55^. The nature of all optimized structures is determined based on the harmonic vibrational frequency calculations performed at the same level to confirm a minimum on the potential energy surface under the indicated symmetry constraint^56^. NBO populations, atomic charges, FMO properties, and second-order perturbation stabilization energies were considered at the same theoretical level. For calculating and visualizing the isoelectronic molecular electrostatic potential (MEP) surface of the studied nitroxide radicals, GaussView 6 was utilized^57^. To gain insights into intermolecular hydrogen bonding, the AIM2000 package^58,59^ was employed to calculate significant AIM topological parameters at the bond critical points of NO…H contacts.

PA and GB are crucial factors determining an atom's or molecule's ability to accept a proton in the gas phase [M_(g)_ + H^+^_(g)_ → MH^+^_(g)_], where M represents the nitroxide radicals. The change in enthalpy, Δ*H*^o^_H_^+^, is derived from the equipartition principle and ideal gas law, while Gibbs energy, Δ*G*^o^_H_^+^, is directly obtained from the translational partition function. We used a value of 6.197 kJ/mol for the enthalpy of the proton, which corresponds to 5/2*RT*. This value was determined by considering the translational entropy *S*(H^+^), determined to be 108.95 J/mol K, and its Gibbs free energy Δ°*G*(H^+^), calculated as 5/2*RT*, resulting in − 26.28 kJ/mol^60^.

The proton affinity of a species M is defined as the negative enthalpy change (− Δ*H*_react_) occurring in the reaction: M_(g)_ + H^+^_(g)_ → MH^+^_(g)_ (*T* = 298 K), where 1 mol of H^+^(g) is utilized to produce 1 mol of the MH^+^(g) species. It is noted that the GB values, represented by the Gibbs free energy change (GB = − Δ*G*), and the protonation entropy (Δ_p_*S*), are determined based on the entropy changes associated with the reaction. Proton affinities were computed as the enthalpies of the reaction (*T* = 298 K)^61.^

.1 $${\text{PA}} = - \Delta H = \left[ {\Delta E_{{{\text{elec}}}} + \Delta {\text{ZPVE}} + \Delta E_{{{\text{vib}}}} - \frac{5}{2}RT} \right]$$

where Δ*E*_elec_ represents the differences in total electronic energy, ΔZPVE is the change in zero-point vibrational energy between TEMPO derivatives and their protonated forms, and Δ*E*_vib_ denotes the temperature-dependent vibrational energy of reactants and products at *T* = 298 K. The study also investigates solvation effects by adding an explicit water molecule that forms hydrogen bonds with the studied structures. The water molecule's hydrogen atom is positioned towards each oxygen and nitrogen atom of the nitroxide moiety in the unprotonated state, allowing for various interactions, including top, planar, and orthogonal configurations, based on the findings by Brás et al.^27^.

## Results and discussion

### Structural and energetic features of TEMPO derivatives and protonated forms

**Scheme 1.**
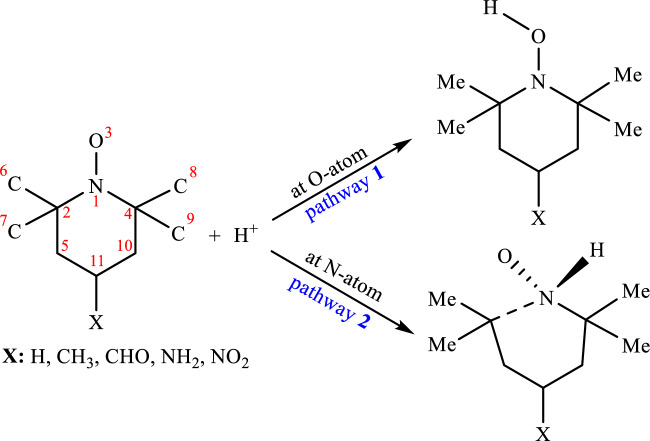
Two possible sites for protonation of the studied radicals in the nitroxide group.

The optimized geometries of all species in the gas phase obtained from these calculations with the B3LYP and M06-2X methods, along with the 6–311++ G(d,p) basis set, were used for further analyses and are summarized in Table 1. Systematic conformational searching was employed when necessary to select the minimum-energy conformation at these theoretical levels. The nature of each stationary point was established through frequency calculations performed at the same theoretical levels. The equilibrium structure of a protonated molecule is established by maximizing the attraction of electron density within the molecule while minimizing the repulsion between its atomic nuclei. As presented in Table 1, bond lengths and bond angles are derived from the optimized structures. To validate the theoretical approach under investigation, the data obtained through the considered DFT methods are compared with available experimental data. TEMPO derivatives, comprising N and O atoms with lone pairs of electrons, are explored with protonation at the oxygen site forming the minimum energy structure, as shown in Table 2. On the other hand, pronation at the nitrogen atom induces the ring opening of cyclic analogs, as shown in Table 1 (cleavage of the N_1_–C_2_ bond in the N-protonation site) and depicted in Scheme 1.

**Table 1:** The primary structural parameters of all examined nitroxide radicals and their predicted protonated forms using the B3LYP and M06-2X (in parenthesis) methods. (Atom labeling in Scheme 1).

Species	Parameter								
	*r*(N_1_–C_4_)	*r*(C_4_–C_10_)	*r*(C_10_–C_11_)	*r*(C_5_–C_11_)	*r*(C_2_–C_5_)	*r*(N_1_–C_2_)	*r*(N_1_–O_3_)	*r*(C_11_–X)	∠C_2_–N_1_–C_4_
TEMPO	1.506 (1.493)	1.541 (1.533)	1.528 (1.524)	1.528 (1.524)	1.541 (1.533)	1.506 (1.493)	1.280 (1.270)	1.094 (1.093)	124.57 (123.89)
TEMPO-CH_3_	1.504 (1.492)	1.540 (1.532)	1.531 (1.526)	1.531 (1.526)	1.540 (1.532)	1.504 (1.492)	1.280 (1.270)	1.533 (1.527)	124.37 (123.80)
TEMPO-CHO	1.503 (1.491)	1.541 (1.533)	1.537 (1.531)	1.537 (1.531)	1.541 (1.533)	1.503 (1.491)	1.279 (1.270)	1.513 (1.509)	124.35 (123.78)
TEMPO-NH_2_	1.505 (1.492)	1.540 (1.531)	1.527 (1.522)	1.527 (1.522)	1.540 (1.531)	1.505 (1.492)	1.280 (1.270)	1.471 (1.464)	124.50 (123.93)
TEMPO-NO_2_	1.505 (1.492)	1.542 (1.533)	1.520 (1.520)	1.524 (1.520)	1.542 (1.533)	1.505 (1.492)	1.279 (1.269)	1.526 (1.512)	124.32 (123.95)
*O-protonation*									
TEMPO-H^+^	1.506 (1.495)	1.544 (1.535)	1.528 (1.524)	1.529 (1.524)	1.543 (1.535)	1.511 (1.498)	1.334 (1.317)	1.092 (1.091)	128.67 (127.97)
TEMPO-CH_3_-H^+^	1.504 (1.493)	1.543 (1.534)	1.532 (1.527)	1.533 (1.527)	1.542 (1.534)	1.508 (1.496)	1.335 (1.317)	1.533 (1.527)	128.52 (127.88)
TEMPO-CHO-H^+^	1.504 (1.494)	1.543 (1.534)	1.534 (1.528)	1.535 (1.530)	1.542 (1.534)	1.509 (1.497)	1.333 (1.316)	1.528 (1.522)	128.55 (127.91)
TEMPO-NH_2_-H^+^	1.504 (1.493)	1.541 (1.532)	1.531 (1.525)	1.532 (1.526)	1.539 (1.532)	1.509 (1.497)	1.334 (1.317)	1.460 (1.455)	128.53 (127.88)
TEMPO-NO_2_-H^+^	1.507 (1.496)	1.542 (1.533)	1.526 (1.522)	1.521 (1.516)	1.543 (1.534)	1.511 (1.499)	1.332 (1.315)	1.536 (1.521)	128.40 (127.74)
*N-protonation*									
TEMPO-H^+^	1.514 (1.556)	1.543 (1.529)	1.534 (1.527)	1.546 (1.527)	1.485 (1.528)	2.428 (1.608)	1.261 (1.346)	1.092 (1.091)	112.53 (119.66)
TEMPO-CH_3_-H^+^	1.514 (1.555)	1.542 (1.528)	1.539 (1.530)	1.552 (1.530)	1.484 (1.527)	2.378 (1.605)	1.262 (1.346)	1.536 (1.528)	112.51 (119.44)
TEMPO-CHO-H^+^	1.513 (1.551)	1.540 (1.530)	1.532 (1.533)	1.551 (1.524)	1.486 (1.525)	2.451 (1.615)	1.258 (1.345)	1.537 (1.523)	111.98 (119.74)
TEMPO-NH_2_-H^+^	1.514 (1.554)	1.541 (1.527)	1.536 (1.529)	1.555 (1.530)	1.482 (1.525)	2.380 (1.606)	1.262 (1.346)	1.460 (1.455)	112.58 (119.40)
TEMPO-NO_2_-H^+^	1.514 (1.554)	1.544 (1.528)	1.528 (1.528)	1.533 (1.524)	1.480 (1.519)	2.482 (1.609)	1.260 (1.347)	1.558 (1.522)	111.98 (119.61)

^a^Bond lengths are given in angstroms (Å), and bond angles are given in degrees.

**Table 2:** Energetic and thermodynamic parameters (in a.u.) for the studied TEMPO derivatives and protonated forms using the B3LYP (in parentheses) and M062X (in bracket) levels of theory (*P* = 1 atm, *T* = 298 K).

Parameter	Species				
	TEMPO-CH_3_	TEMPO	TEMPO-NH_2_	TEMPO-CHO	TEMPO-NO_2_
NO radicals					
*E*	− 522.893 {− 522.645}^a^	− 483.595 {− 483.367}^a^	− 538.946 {− 538.698}^a^	− 596.936 {− 596.669}^a^	− 688.157 {− 687.857}^a^
*H*_thermal_	− 522.879 {− 522.631}^a^	− 483.582 {− 483.354}^a^	− 538.931 {− 538.683}^a^	− 596.920 {− 596.654}^a^	− 688.141 {− 687.842}^a^
G_thermal_	− 522.932 {− 522.683}^a^	− 483.632 {− 483.403}^a^	− 538.984 {− 538.735}^a^	− 596.976 {− 596.709}^a^	− 688.199 {− 687.898}^a^
*S*	111.531 {109.862}^a^	104.590 {103.086}^a^	110.916 {109.276}^a^	116.865 {115.454}^a^	120.573 {119.305}^a^
O-protonated					
*E* (Δ*E*)	− 523.233 (− 212.898)^b^ {− 522.981}^a^ [− 211.408]^c^	− 483.934 (− 212.472)^b^ {− 483.702}^a^ [− 210.969]^c^	− 539.284 (− 212.086)^b^ {− 539.032}^a^ [− 210.843]^c^	− 597.265 (− 206.815)^b^ {− 596.995}^a^ [− 205.447]^c^	− 688.479 (− 202.196)^b^ {− 688.176}^a^ [− 200.992]^c^
*H*_thermal_ (Δ*H*)	− 523.218 (− 212.678)^b^ {− 522.967}^a^ [− 211.220]^c^	− 483.921 (− 212.231)^b^ {− 483.689}^a^ [− 210.781]^c^	− 539.269 (− 211.849)^b^ {− 539.018}^a^ [− 210.592]^c^	− 597.250 (− 206.539)^b^ {− 596.980}^a^ [− 205.698]^c^	− 688.463 (− 201.982)^b^ {− 688.160}^a^ [− 200.301]^c^
G_thermal_ (Δ*G*)	− 523.271 (− 213.052)^b^ {− 523.020}^a^ [− 211.659]^c^	− 483.971 (− 212.658)^b^ {− 483.739}^a^ [− 211.157]^c^	− 539.322 (− 212.267)^b^ {− 539.070}^a^ [− 210.969]^c^	− 597.306 (− 207.290)^b^ {− 597.036}^a^ [− 204.694]^c^	− 688.521 (− 202.352)^b^ {− 688.217}^a^ [− 202.309]^c^
*S* (Δ*S*)	112.785 (1.254)^b^ <111.252>^d^ [1.354]^e^	106.023 (1.433)^b^ <104.512>^d^ [1.328]^e^	112.316 (1.400)^b^ <110.627>^d^ [1.316]^e^	119.383 (2.518)^b^ <118.263>^d^ [− 3.441]^e^	121.814 (1.241)^b^ <119.585>^d^ [6.651]^e^
N-protonated					
*E* (Δ*E*)	− 523.200 (− 192.132)^b^ {− 522.943}^a^ [− 186.832]^c^	− 483.902 (− 192.306)^b^ {− 483.664}^a^ [− 186.379]^c^	− 539.251 (− 191.536)^b^ {− 538.994}^a^ [− 186.077]^c^	− 597.239 (− 190.178)^b^ {− 596.960}^a^ [− 182.646]^c^	− 688.447 (− 181.758)^b^ {− 688.137}^a^ [− 175.314]^c^
*H*_thermal_ (Δ*H*)	− 523.184 (− 191.279)^b^ {− 522.928}^a^ [− 186.594]^c^	− 483.887 (− 191.377)^b^ {− 483.651}^a^ [− 186.138]^c^	− 539.235 (− 190.680)^b^ {− 538.980}^a^ [− 185.851]^c^	− 597.222 (− 189.257)^b^ {− 596.945}^a^ [− 182.518]^c^	− 688.430 (− 180.860)^b^ {− 688.121}^a^ [− 175.153]^c^
G_thermal_ (Δ*G*)	− 523.240 (− 193.294)^b^ {− 522.981}^a^ [− 186.968]^c^	− 483.941 (− 193.588)^b^ {− 483.701}^a^ [− 186.524]^c^	− 539.291 (− 192.665)^b^ {− 539.032}^a^ [− 186.205]^c^	− 597.281 (− 191.426)^b^ {− 597.000}^a^ [− 182.451]^c^	− 688.491 (− 183.673)^b^ {− 688.178}^a^ [− 178.206]^c^
*S* (Δ*S*)	118.290 (6.759)^b^ <111.121>^d^ [1.259]^e^	112.006 (7.416)^b^ <104.379>^d^ [1.293]^e^	117.574 (6.658)^b^ <110.466>^d^ [1.190]^e^	124.139 (7.274)^b^ <115.233>^d^ [− 0.221]^e^	130.007 (9.434)^b^ <119.483>^d^ [0.178]^e^

^a^All data in “braces” were calculated at the M06-2X/6–311++ G(d,p) level are given in a.u

^b^All relative energies in the “parenthesis” were calculated at the B3LYP/6–311++ G(d,p) level and given in kcal/mol.

^c^All relative energies in the “square bracket” were calculated at the M06-2X/6–311++ G(d,p) level and given in kcal/mol.

^d^All entropies in “angle bracket” were calculated at the M06-2X/6–311++ G(d,p) level and given in cal/mol.K

^e^Relative entropies were calculated at the M06-2X/6–311++ G(d,p) level and given in cal/mol.K.

As indicated in Table 1, protonation at the N-atom leads to increased instability in the resulting species. This protonation induces a reduction in the C_2_–N_1_–C_4_ bond angle, typically falling within the range of 111.98° (119.61°) to 112.58° (119.66°), and an elongation of the N_1_–C_2_ bond length, increasing from 2.378 Å (1.605°) to 2.482 Å (1.609°) at the B3LYP (M06-2X) method. These effects are notably pronounced in the protonation of TEMPO derivatives with methyl-containing substitutions, which serve as moderate electron donors. In contrast, protonation, through chemical bonding to the oxygen atom of the nitroxide moiety, stabilizes the studied structures. Upon protonation of the oxygen atom, the C_2_–N_1_–C_4_ bond angle increases compared to the studied TEMPO derivatives, with the observed range for this angle being 128.40° (127.74°) to 128.67° (127.88°) at the B3LYP (M06-2X) method (see Table 1). The calculated structures of TEMPO derivatives and their protonated forms reveal that they all adopt chair-type conformations, with the acidic proton occupying an equatorial position. In these radicals, the oxygen atom in the nitroxide moiety is the preferred site to receive the proton.

The difference in bond lengths between the B3LYP and M06-2X methods can be attributed to their distinct treatment of electron correlation effects, exchange, and dispersion interactions. These methodological disparities result in variations in the calculated energies and geometries of molecular systems. Specifically, the elongation of the N_1_–C_2_ bond and reduction in the C_2_–N_1_–C_4_ bond angle observed at the protonated N-atom may be influenced differently by the exchange–correlation functionals used in B3LYP and M06-2X. The variations in these functionals lead to different electronic structures, resulting in differing bond lengths and angles. The advantages of the M06-2X method over the B3LYP method include its utilization of sophisticated density functionals, leading to higher accuracy in predicting electronic properties, effective consideration of molecular vibrations, superior incorporation of dispersion effects, and computational efficiency, making it suitable for large-scale and complex studies. As depicted in Table 1, this issue can be easily seen in bond lengths, particularly the N_1_–C_2_ bond length observed for the N-protonation of the TEMPO derivatives.

As illustrated in Table 1, the most significant alteration occurs in the N–O bond length of the examined molecules. Before protonation, which takes place at the oxygen atom of the NO moiety, the original N–O bond lengths ranged from 127.866 (127.016 pm) to 128.018 (126.889 pm) at the B3LYP (M06-2X) method. Following protonation at the oxygen atom, these values increase to a range of 133.167 (131.474 pm) to 133.456 (131.740 pm) at the B3LYP (M06-2X) method. The enthalpies and Gibbs free energies for both the neutral and the O-protonated or N-protonated forms of the TEMPO derivatives were calculated.

We present predictions for the energetic and thermodynamic parameters of TEMPO derivatives, including their N- and O-protonated forms, as detailed in Table 2. Protonation is observed as an exothermic reaction, with the standard enthalpy of O-protonated conformers exceeding the N-protonated value by 21.40 (24.74) kcal/mol at the B3LYP (M06-2X) method. We may use the enthalpy and entropy data to calculate benchmark PA and GB. Notably, there is a significant difference in Gibbs free energy (6.30 kJ/mol) and enthalpy of the proton (1.48 kcal/mol), resulting in a total difference of 7.42 (1.29) kcal/mol between N-protonation and O-protonation at the B3LYP (M06-2X) method (refer to Table 2). The study identifies nitrogen and oxygen atoms as potential protonation sites in the considered nitroxide radicals. The studied DFT methods calculations show that oxygen protonation (pathway **1**) is more stable than nitrogen protonation (pathway **2**), with an energy difference of 16.64–20.77 kcal/mol (22.80–25.68 kcal/mol) at the B3LYP (M06-2X) method.

### Natural population analysis (NPA) atomic charges

The Mulliken population analysis is a popular model for predicting individual atomic charges due to its computational simplicity^62,63^. However, it has a high sensitivity to the basis set and unpredictability, causing fluctuations in partial charges. Atomic charges are crucial for identifying active sites, as excessive charge affects a molecule's interaction with a charge depletion receptor site. Calculating NPA charges is essential for electronic structure, dipole moment, and molecular reactivity^64^. (see Table S2 in the Supplementary Information, SI). Table S2 displays the Mulliken atomic charges of TEMPO derivatives and their protonated forms at the B3LYP/6–311++ G(d,p) and M06-2X/6–311++ G(d,p) (in parenthesis) levels of theory. The unpaired spin density is primarily located in the NO moiety. Nitrogen atoms have a slightly lower spin density than oxygen atoms. The incorporation of substituents in the NO radical structure results in a fractional redistribution of unpaired spin density. For instance, the presence of an ED substituent (X: –CH_3_) slightly increases spin density on the N-atom, causing a partial transfer of unpaired electron spin density. However, the introduction of an EW substituent (X: –NO_2_) induces a significant shift in spin density from the N-atom to the O-atom due to a higher contribution from the resonance structure ⟩ N–O^·^ compared to the dipolar resonance structure ⟩ N^·+^–O^–^, leading to a reduction in spin density on the N atom^65^.

As seen in Table S2, the addition of a methyl group increases the PA of the nitrogen atom in TEMPO. Furthermore, the inclusion of a methyl group in TEMPO leads to increased basicity, as demonstrated by the PA of TEMPO-CH_3_ being 896.04 (887.35) kJ/mol, which is 1.87 (1.90) kJ/mol higher than that of TEMPO itself at the B3LYP (M06-2X) method. This suggests that the methyl group specifically stabilizes the carbocations formed upon the protonation of a ring carbon atom.

It is noteworthy that the NH_2_ group, commonly considered an electron-donating group, exhibits electron-withdrawing behavior in the proton affinity and gas-phase basicity values of TEMPO derivatives. This unexpected behavior challenges conventional expectations, as inductive effects typically involve the transmission of electron density through *σ* bonds, with more electronegative atoms withdrawing electron density from neighboring atoms. In the case of the amino group (–NH_2_), the nitrogen atom is more electronegative than the attached carbon atoms. Therefore, the inductive effect of the nitrogen atom would be expected to withdraw electron density from the rest of the molecule, resulting in decreased basicity or proton affinity. This discrepancy between the expected electronic effects based on the classification of the –NH_2_ group as an electron-donating group and the observed results highlights the importance of considering both inductive and mesomeric effects in understanding the electronic properties of functional groups in molecules. In this case, the inductive effect of the –NH_2_ group appears to have a more significant influence on the observed proton affinity and gas-phase basicity values.

The PA for O-protonation exceeds that for N-protonation by 89.54 (103.10) kJ/mol at the B3LYP (M06-2X) method. Similarly, the standard GB for O-protonation is 82.66 (103.23) kJ/mol higher than that for N-protonation, indicating a 9.63 (3.78) kJ/mol difference in entropy effects between the two protonation processes using the B3LYP (M06-2X) method. To provide context, these PA and GB values can be compared with available experimental data, which indicate a proton affinity of 882.3 kJ/mol and a gas-phase basicity of 849.8 kJ/mol for TEMPO^61^.

The computed PA values for TEMPO using the B3LYP and M06-2X methods are 894.17 and 885.45 kJ/mol, respectively, showing a difference of around 1.35% and 0.36% compared to the experiment. This disparity can be attributed to differences in how these two DFT methods handle electronic structure and interactions. M06-2X features a high exact exchange fraction of 54%, relying on Hartree–Fock exchange, which is particularly suited for systems with strong static electron correlation effects^51,52^. In contrast, B3LYP has a lower fraction of 20%, emphasizing DFT exchange–correlation functionals. As a result, the outcomes obtained with M06-2X are in good agreement with available experimental results. This observation is consistent with the tendency of more sophisticated methods and higher basis sets to yield more accurate predictions. As can be seen in Table 3, the PAs for O-protonation of the other NO radicals, namely TEMPO-NO_2_, TEMPO-CHO, TEMPO-NH_2_, and TEMPO-CH_3_, were determined as 851.29 (842.14), 870.36 (861.20), 892.57 (883.83), and 896.04 (887.35) kJ/mol, respectively, using the B3LYP (M06-2X) method. Unfortunately, there are no experimental values available for these derivatives to compare directly.

**Table 3:** The HOMO, LUMO, and HOMO–LUMO gap (in eV), as well as the PA, and GB upon O-protonation and N-protonation of the NO moiety for the studied radicals (in kJ/mol). These values were calculated using the B3LYP and M06-2X (in parenthesis) methods.

Parameter	Species				
	TEMPO-CH_3_	TEMPO	TEMPO-NH_2_	TEMPO-CHO	TEMPO-NO_2_
HOMO	− 5.3225 (− 6.9966)	− 5.3225 (− 6.9985)	− 5.3606 (− 7.0347)	− 5.6327 (− 7.3109)	− 5.8423 (− 7.5424)
LUMO	− 0.3755 (− 0.1801)	− 0.3755 (− 0.1695)	− 0.4952 (− 0.2778)	− 1.3415 (− 0.1973)	− 2.4300 (− 0.8419)
HOMO–LUMO gap	4.947 (6.816)	4.947 (6.829)	4.865 (6.757)	4.291 (7.114)	3.412 (6.701)
Hybrid, NO radical (M)					
B3LYP	(48.16%) 0.694*N_1_ *sp*^2.65^	(48.12%) 0.694*N_1_ *sp*^2.65^	(48.17%) 0.694*N_1_ *sp*^2.65^	(48.24%) 0.695*N_1_ *sp*^2.64^	(48.30%) 0.695*N_1_ *sp*^2.63^
	(51.84%) 0.720*O_3_ *sp*^3.11^	(51.88%) 0.720*O_3_ *sp*^3.11^	(51.83%) 0.720*O_3_ *sp*^3.11^	(51.76%) 0.719*O_3_ *sp*^3.12^	(51.70%) 0.719*O_3_ *sp*^3.12^
M06-2X	(48.16%) 0.694*N_1_ *sp*^2.65^	(48.12%) 0.694*N_1_ *sp*^2.65^	(47.92%) 0.692*N_1_ *sp*^2.86^	(48.24%) 0.695*N_1_ *sp*^2.64^	(48.31%) 0.695*N_1_ *sp*^2.62^
	(51.86%) 0.720*O_3_ *sp*^2.96^	(51.88%) 0.720*O_3_ *sp*^2.96^	(51.83%) 0.722*O_3_ *sp*^3.10^	(51.76%) 0.719*O_3_ *sp*^2.98^	(51.69%) 0.719*O_3_ *sp*^2.98^
Spin density					
N	0.4591 (0.4223)	0.4545 (0.4202)	0.4496 (0.4115)	0.4549 (0.4224)	0.4379 (0.3943)
O	0.5190 (0.5530)	0.5214 (0.5545)	0.5229 (0.5563)	0.5234 (0.5559)	0.5311 (0.5655)
*O-protonation*					
HOMO	− 11.8696 (− 13.4786)	− 11.9158 (− 13.5289)	− 10.2042 (− 11.9479)	− 10.5226 (− 12.3216)	− 11.7036 (− 13.6968)
LUMO	− 4.1606 (− 3.5380)	− 4.1905 (− 3.5655)	− 4.1905 (− 3.5592)	− 4.5225 (− 3.7269)	− 5.5049 (− 4.0727)
HOMO–LUMO gap	7.709 (9.941)	7.725 (9.963)	6.014 (8.389)	6.000 (8.595)	6.199 (9.624)
PA	896.04 (887.35)	894.17 (885.45) [882.3]^*a*^	892.57 (883.83)	870.36 (861.20)	851.29 (842.14)
GB	865.16 (856.63)	863.51 (854.78) [849.8]^*a*^	861.87 (853.06)	841.05 (832.25)	820.39 (810.04)
Hybrid, protonated (MH^+^)					
B3LYP	(42.92%) 0.655*N_1_ *sp*^3.55^	(42.91%) 0.655*N_1_ *sp*^3.54^	(42.93%) 0.655*N_1_ *sp*^3.53^	(43.00%) 0.656*N_1_ *sp*^3.52^	(43.04%) 0.656*N_1_ *sp*^3.4^
	(57.08%) 0.756*O_3_ *sp*^3.05^	(57.09%) 0.756*O_3_ *sp*^3.05^	(57.07%) 0.756*O_3_ *sp*^3.05^	(57.00%) 0.755*O_3_ *sp*^3.06^	(56.96%) 0.755*O_3_ *sp*^3.06^
M06-2X	(42.52%) 0.652*N_1_ *sp*^3.49^	(42.52%) 0.652*N_1_ *sp*^3.49^	(42.54%) 0.652*N_1_ *sp*^3.48^	(42.59%) 0.653*N_1_ *sp*^3.47^	(42.66%) 0.653*N_1_ *sp*^3.50^
	(57.48%) 0.758*O_3_ *sp*^2.80^	(57.48%) 0.758*O_3_ *sp*^2.80^	(57.46%) 0.758*O_3_ *sp*^2.80^	(57.41%) 0.758*O_3_ *sp*^2.82^	(57.34%) 0.757*O_3_ *sp*^2.97^
Spin density					
N	0.7176 (0.7052)	0.7124 (0.7005)	0.7137 (0.7015)	0.7161 (0.7049)	0.7072 (0.7054)
O	0.2296 (0.2408)	0.2324 (0.2440)	0.2317 (0.2428)	0.2324 (0.2438)	0.2373 (0.2397)
*N-protonation*					
HOMO	− 10.8600 (− 14.2481)	− 10.8111 (− 14.6193)	− 10.5117 (− 12.1314)	− 10.8628 (− 12.610)	− 11.0668 (− 13.902)
LUMO	− 7.1811 (− 3.5620)	− 7.3416 (− 3.6088)	− 7.2219 (− 3.5190)	− 7.5865 (− 3.6727)	− 7.7960 (− 4.1062)
HOMO–LUMO gap	3.679 (10.686)	3.469 (11.011)	3.290 (8.612)	3.276 (8.937)	3.271 (9.796)
PA	806.50 (786.90)	806.92 (785.00)	804.08 (783.80)	787.45 (769.85)	763.11 (739.04)
GB	782.50 (756.02)	783.72 (754.16)	780.16 (752.83)	763.25 (737.12)	741.84 (706.81)
Hybrid, protonated (MH^+^)					
B3LYP	(48.19%) 0.694*N_1_ *sp*^2.54^	(48.14%) 0.694*N_1_ *sp*^2.51^	(48.21%) 0.694*N_1_ *sp*^2.53^	(48.10%) 0.694*N_1_ *sp*^2.49^	(48.18%) 0.694*N_1_ *sp*^2.47^
	(51.81%) 0.720*O_3_ *sp*^3.06^	(51.86%) 0.720*O_3_ *sp*^3.05^	(51.79%) 0.720*O_3_ *sp*^3.06^	(51.90%) 0.720*O_3_ *sp*^3.02^	( 51.82%) 0.720*O_3_ *sp*^3.05^
M06-2X	(50.24%) 0.709*N_1_ *sp*^3.99^	(50.24%) 0.709*N_1_ *sp*^4.00^	(50.26%) 0.709*N_1_ *sp*^3.99^	(50.30%) 0.709*N_1_ *sp*^3.98^	(50.45%) 0.710*N_1_ *sp*^3.99^
	(49.76%) 0.705*O_3_ *sp*^4.23^	(49.76%) 0.705*O_3_ *sp*^4.24^	(49.74%) 0.705*O_3_ *sp*^4.24^	(49.70%) 0.705*O_3_ *sp*^4.24^	( 49.55%) 0.704*O_3_ *sp*^4.30^
Spin density					
N	0.1965 (− 0.0637)	− 0.0252 (− 0.0628)	− 0.0252 (− 0.0558)	− 0.0331 (− 0.0562)	0.2215 (− 0.0586)
O	0.5353 (0.9155)	0.8167 (0.9179)	0.8206 (0.9155)	0.8350 (0.9148)	0.5180 (0.9185)

^a^Experimental PA and GB values. (Ref.^61^).

It is worth noting that compounds containing EW groups (X: –CHO, –NO_2_) exhibit lower PAs compared to TEMPO, indicating their stability consistent with their negative heats of formation. Moreover, these compounds exhibit lower basicity when compared to compounds containing ED groups (X: –NH_2_, –CH_3_). This observation can be attributed to the presence of the O-atom, which possesses higher electronegativity than the N-atom and acts as a weaker π-donor group. The presence of a potent EW group such as NO_2_ in the piperidine ring significantly reduces the basicity by 43.12 (44.74) kJ/mol at the B3LYP (M06-2X) method compared to its counterparts, resulting in the lowest PA among the investigated compounds (see Table 2).

Upon examination of the results, it becomes apparent that the studied TEMPO derivatives are potent bases, with PA values ranging from 750 to 900 kJ/mol. The estimated PA of TEMPO using the B3LYP method serves as a reference for super-basicity. Nitroxide radicals with PA values lower or higher than TEMPO are classified as superbases. Following this classification, the examined substituents can be ranked in terms of their basicity as follows: –CH_3_ > –H > –NH_2_ > –CHO > –NO_2_. Consequently, the methyl substituent, exhibiting a higher PA, reveals stronger basicity, while the nitrate substituent, with a lower PA, indicates weaker basicity.

Moreover, the NBO analysis provides insights into the percentage of NBO contribution to the N–O hybrid bond through natural atomic hybrids (NAHs). This analysis reveals that approximately 48% and 52% of the NBOs contribute to the N_1_ and O_3_ hybrid orbitals, respectively (see Table 3). Furthermore, the hybrid labels indicate that the N_1_ hybrid orbital is *sp*^2.63–2.65^ (*sp*^3.48–3.55^), while the hybrid orbital on O_3_ is *sp*^3.11–3.12^ (*sp*^3.05–3.06^) at the B3LYP (M06-2X) method. Regarding the protonation of the considered TEMPO derivatives, two scenarios arise depending on the attachment of the hydrogen atom:

1. When the proton (H^+^) is attached to the oxygen atom in the nitroxide moiety, the NAHs on the N–O bond contribute nearly 43% and 57% to the N_1_ and O_3_ hybrid orbitals, respectively. Moreover, the polarization coefficients are around 0.66 and 0.76 for nitrogen and oxygen atoms, respectively. The hybrid labels for the N_1_ and O_3_ hybrid orbitals are *sp*^3.48–3.55^ (*sp*^3.47–3.50^) and *sp*^3.05–3.06^ (*sp*^2.80–2.97^), respectively, at the B3LYP (M06-2X) method.
2. When the proton attacks the nitrogen atom in the NO moiety, the NAHs on the N–O bond contribute nearly 48% (50%) to the N_1_ and 52% (50%) to the O_3_ hybrid orbitals, at the B3LYP (M06-2X) method. Also, the polarization coefficients are around 0.69 (0.71) and 0.72 (0.71) for nitrogen and oxygen atoms, respectively. The hybrid labels for the N_1_ and O_3_ hybrid orbitals are *sp*^2.47–2.54^ (*sp*^3.99–4.00^) and *sp*^3.02–3.06^ (*sp*^4.23–4.30^), respectively.

The considered DFT methods calculations on TEMPO derivatives reveal that protonation at the oxygen atom (pathway **1**) is more favorable than protonation at the nitrogen atom (pathway **2**).

The characteristics of the investigated groups appear to have an impact on the proton affinity of the studied NO radicals. Introducing electron-donating groups at the *para*-position of the piperidine ring, relative to the nitroxide moiety, increases electron density around that particular group, as observed in the electronic distribution of the HOMO and LUMO surfaces. Thus, it is found that compounds featuring a methyl group substitution display higher PA values than TEMPO. In contrast, when electron-withdrawing groups are substituted at the same position within the piperidine ring, they reduce the electron density around the nitroxide moiety. As a result, compounds with electron-withdrawing groups exhibit lower PA values compared to TEMPO, as shown in Fig. S1 of the Supplementary Information.

The observed results can be explained by the electronic interactions between substituent groups and electrons within the piperidine ring. In the case of NH_2_, the more electronegative nitrogen atom in the ring induces an inductive effect, withdrawing electrons. However, the nitrogen atom also possesses lone pairs of electrons, which function as π-donors. These π-donor interactions prevail over the inductive effect, contributing additional electron density to the nitroxide group. Instead, the NO_2_ group, with its positive charge due to electron sharing with oxygen atoms, is relatively unstable and experiences an unfavorable resonance effect, which draws electrons away from the ring, resulting in a decrease in the electron density.

As seen in Table 3, the HOMO analysis of TEMPO derivatives reveals that the central nitrogen atom in the nitroxide moiety is more prone to protonation than other atoms, as the HOMO is primarily located on the central nitrogen atom. The analysis in Table 3, along with Figs. S1–S3 in the Supplementary Information, shows the energies of both the HOMO and LUMO when transitioning from natural TEMPO derivatives to their O- and N-protonated states.

At the B3LYP method, we observe an increase in the HOMO–LUMO energy gap for the O-protonated forms. This suggests that protonation at the oxygen atom stabilizes the frontier molecular orbitals (HOMO and LUMO), resulting in a larger energy gap. This effect may arise from the increased electronegativity of the oxygen atom compared to nitrogen, leading to a stronger interaction with the proton. Conversely, for the N-protonated forms, there is a decrease in the HOMO–LUMO energy gap, indicating destabilization of the frontier molecular orbitals upon protonation at the nitrogen atom. This could be attributed to the disruption of the original electronic structure caused by the addition of a proton to the nitrogen atom. Additionally, at the M06-2X method, we observe an increase in the HOMO–LUMO energy gap for both protonated forms. This suggests a more general trend of stabilization of the frontier molecular orbitals upon protonation, regardless of the protonation site. The M06-2X functional may handle the electronic structure and interactions differently compared to B3LYP, resulting in this contrasting behavior.

Overall, the differences in the HOMO–LUMO energy gap changes between the two methods likely stem from the inherent approximations and parameterizations employed by each method in describing electronic structure and molecular interactions. The B3LYP and M06-2X functionals have different exchange–correlation components and levels of dispersion correction, leading to variations in their predictions for the electronic properties of molecules, especially in cases involving significant structural modifications such as protonation. Additionally, the sensitivity of these methods to the specific electronic environment and bonding characteristics of the system can also contribute to the observed differences.

### Molecular electrostatic potential (MEP) analysis

The molecular electrostatic potential (MEP) is a crucial tool in understanding proton affinity, as it is used to study electrophilic reactivity. Negative MEP values increase molecular basicity, proton affinity, and gas-phase basicity. In systems with multiple sites of the same basic element type, the most basic site has the most negative MEP value, indicating a stronger molecular basicity. This understanding is essential for understanding proton affinity and gas-phase basicity^66,67^.

**Figure 1:**
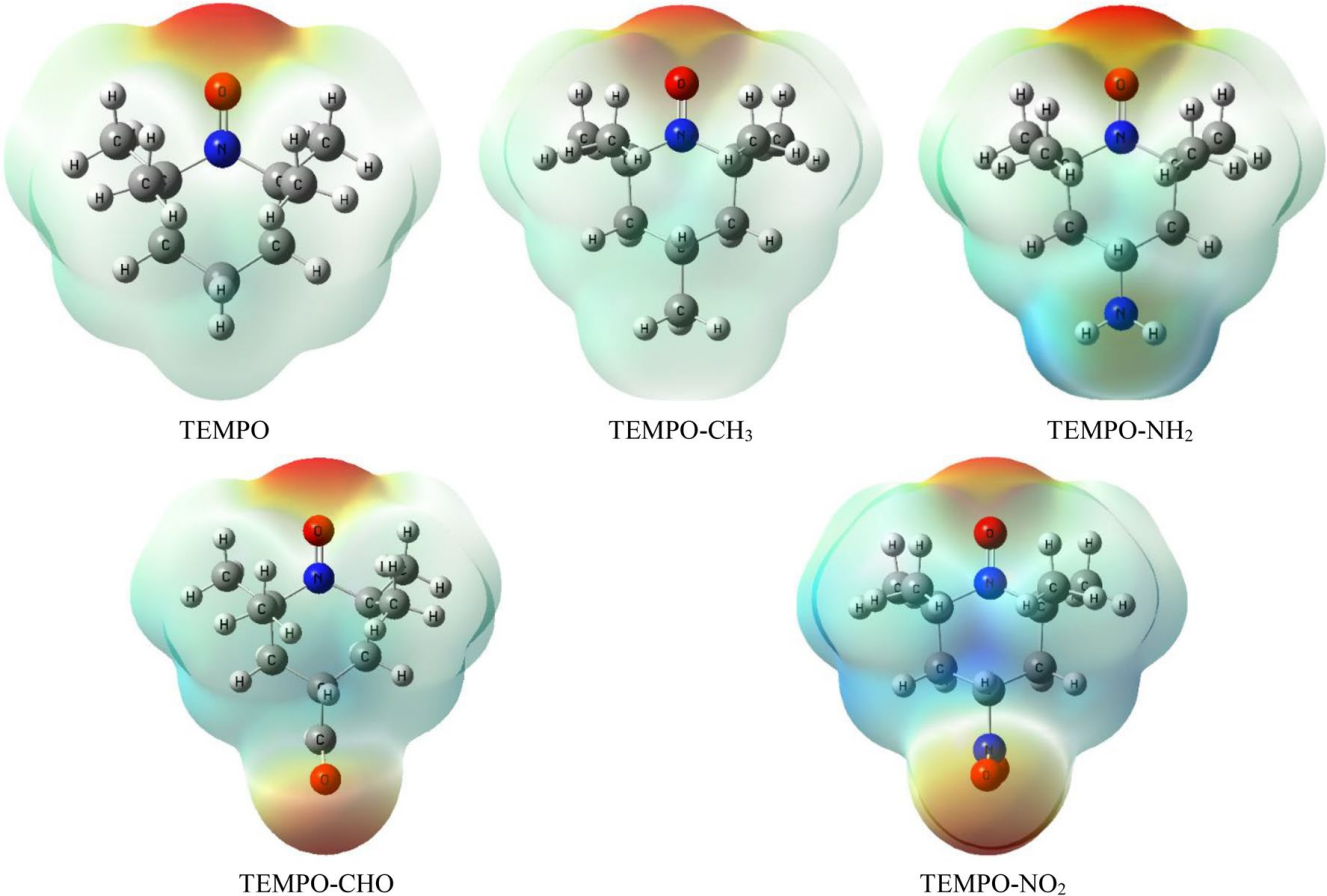
The 3D-representative molecular structures of the electrostatic potential surface of the studied TEMPO derivatives at the M06-2X/6–311++ G(d,p) level.

The MEP serves as a useful tool for illustrating charge distributions in terms of atomic charge distributions, with red and blue indicating high and low electron density, respectively, representing electrophilic and nucleophilic sites^68^. As shown in Fig. 1, the MEP map of TEMPO derivatives indicates that the oxygen atom of the nitroxide moiety has the most negative electrostatic potential, suggesting that it is more *electron-rich* than other parts of the studied molecules. Therefore, the oxygen atom is the most preferable reactive site for electrophilic reactions. MEP maps illustrate charge delocalization and reactive sites for nucleophilic and electrophilic attacks, revealing the main negative potential regions around the oxygen atom of the nitroxide group. They also validate the different negative and positive potential sites of the molecule under the total electron density surface (see Fig. S3 in the SI). The ESPs of the TEMPO derivatives and their O-protonated and N-protonated species at the studied DFT methods are presented in Table S3–S8 in the SI. The reported data in these tables shows that for both O-protonation and N-protonation scenarios, the ESPs at nitrogen (N_1_) and oxygen (O_3_) atoms in the nitroxide moiety decrease in magnitude compared to their neutral counterparts. This reduction in ESPs suggests a decrease in electron density around these atoms, consistent with the addition of a proton. This decrease in ESPs makes them less nucleophilic or less prone to donating electrons compared to their neutral forms. Understanding these changes in ESP values is crucial for predicting the reactivity and properties of TEMPO derivatives upon protonation, particularly in terms of their potential involvement in electrophilic or nucleophilic reactions.

The MEP's most negative potential value (*V*_min_) is a crucial factor in evaluating through-bond electronic effects in organic molecules^69,70^. *V*_min_ is expected to be located in the lone-pair region of the oxygen atom within the NO moiety, representing the *electron-rich* region of the molecule. Thus, the *V*_min_ value of the oxygen atom in the NO part is employed to assess the electronic effects of nitroxide radicals. The isopotential surface is present around the lone-pair regions of the NO group and extends to the lone pair of the nitrogen atom of the heterocyclic radical. Table 4 provides the *V*_min_ values for all examined radicals, which range from − 22.373 to − 22.392 kcal/mol for substituted systems. The negative character of the NO radical moiety's *V*_min_ points is predicted to be influenced by the ED or EW nature of the substituent at the C_10_ position^70^.

**Table 4:** The deepest (*V*_min_) values (in kcal/mol) and the inductive parameter (σ_I_) of TEMPO derivatives at the M06-2X/6–311++ G(d,p) level of theory.

Species	*V*_min_	σ_I_
TEMPO-NO_2_	− 22.3731	0.66
TEMPO-CHO	− 22.3806	0.46
TEMPO-NH_2_	− 22.3903	0.11
TEMPO-CH_3_	− 22.3917	0.03
TEMPO	− 22.3918	0.00

As presented in Table 4, the introduction of electron-donating alkyl groups (e.g., CH_3_) causes only marginal alterations in *V*_min_ compared to the unsubstituted NO radical (TEMPO). However, the presence of an EW group, such as X = –NO_2_, leads to significant changes in *V*_min_. The impact of substituents is often correlated with electronegativity^70,71^, and in this case, the –NO_2_ group likely exhibits higher electronegativity values, causing a decrease in the negative character of *V*_min_. Electron-donating group effects, like –CH_3_, enhance the electron richness of the nitroxide radical, whereas NO_2_ substitution withdraws electrons from the carbon chain, yielding an electron-poor nitroxide radical. Generally, the shift from EW to ED groups is associated with an increase in the negative value of *V*_min_^26^. In specific cases, the unsubstituted radical (TEMPO) shows more negative *V*_min_ values than CH_3_-substituted radicals due to the steric effects of the methyl group, which deforms of ring structure and reduces electron density on the oxygen atom of the nitroxide functional group^72^. Since the *V*_min_ value corresponding to the NO moiety reflects the ED or EW nature of the substituent, there is a correlation between MEP values and inductive substituent constants, denoted as σ_I_.

**Figure 2:**
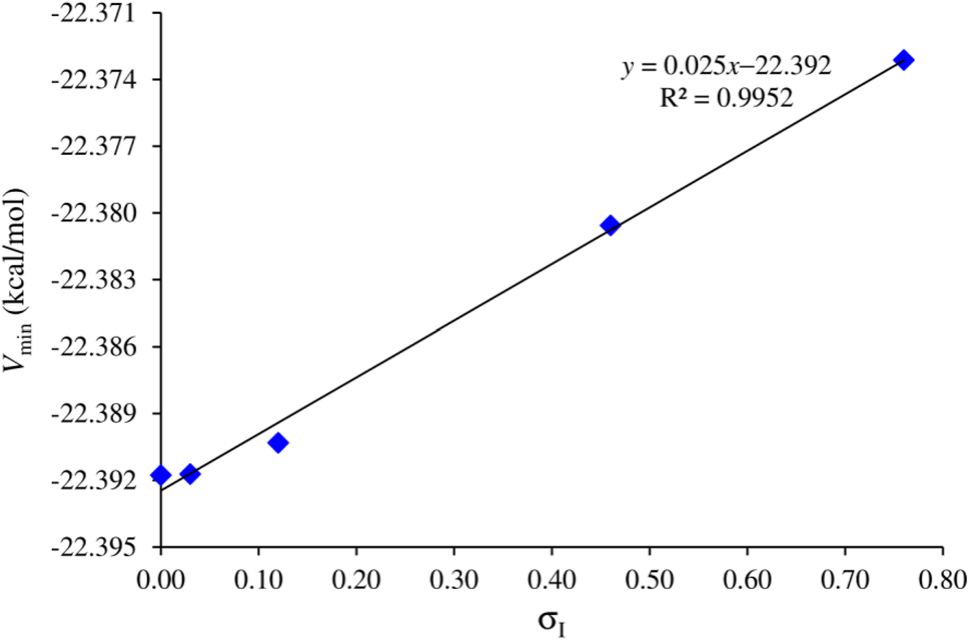
The correlation between σ_I_ and *V*_min_ for TEMPO and its derivatives using the M06-2X method.

As depicted in Fig. 2, the *V*_min_ values of the NO moiety are strongly correlated with the σ_I_ value (*R* = 0.995; with slope and intercept of 0.025 and − 22.392, respectively) at the M06-2X/6–311++ G(d,p) level. This implies that the deepest *V*_min_ accurately measures the inductive effect of substituents. When an atom or group is more electronegative than a hydrogen atom, its EW power is expected to be greater. The σ_I_ parameters align with this pattern, and the magnitude of the *V*_min_ values reflects the higher EW capacity of each substituent compared to a hydrogen atom^70^.

### Natural Bond Orbital (NBO) analysis

Intramolecular electron displacements play a crucial role as they determine the characteristic stability of a compound. The NBO analysis is a valuable tool for assessing hyperconjugation interactions, providing valuable insights into the stabilizing effects of occupancy values and associated delocalization energies^73^. The NBO analysis identifies acceptors and donors in the molecule, their occupancy values, and potential transitions between them. The second perturbation energies (*E*_2_) related to the delocalization *i* → *j* are calculated for each donor NBO(*i*) and acceptor NBO(*j*) expressed as^74^

2 $$E_{2} {\kern 1pt} = \Delta E_{ij} {\kern 1pt} = q_{i} \left[ {{{F_{(i,j)}^{2} } \mathord{\left/ {\vphantom {{F_{(i,j)}^{2} } {(\varepsilon_{i} - \varepsilon_{j} )}}} \right. \kern-0pt} {(\varepsilon_{i} - \varepsilon_{j} )}}} \right]$$

where *q*_*i*_ is the occupancy of the *i*th donor orbital, *F*(*i*,*j*) denotes the off-diagonal NBO Fock matrix elements, and ε_*i*_ and ε_*j*_ are diagonal elements. Intramolecular hyperconjugative interactions involve *σ* and π electrons of C–H, C–C, C–N, N–O, and LP(e)_O_ with the antibonding C–H, C–C, N–O, and C–N bonds, contributing to the stabilization of specific parts of the piperidine ring. Tables S9 and S10 in the Supporting Information present information on the occupancies of NBOs for the studied TEMPO derivatives, facilitating electron transitions that allow movement from donor bonding orbitals to acceptor lone pairs and antibonding orbitals, along with corresponding occupancies and energy levels. This study emphasizes the most significant of these electron transitions. The intramolecular hyperconjugative interaction of the C_6_–H_14_ bond arises from the orbital overlap between the σ_C6–H14_ bonding orbital and the σ^*^_N1–C2_ antibonding orbital, resulting in an average increase in electron density (ED) of 0.0391 a.u. At the B3LYP (M06-2X) method, this interaction yields a stabilization energy of 2.35 (2.72) kcal mol^−1^. Similarly, *σ* → π^*^ interactions occur between the σ_C2–C6_ bonding orbital and the π^*^_N1–O3_ antibonding orbital, increasing the average electron density by approximately 0.0077 (0.0277) a.u., leading to a stabilization of 1.67 (2.20) kcal mol^−1^ for TEMPO derivatives according to the B3LYP (M06-2X) method.

The NBO analysis also emphasizes the exclusive *p*-character (> 99.95%) of the second lone pair of oxygen, LP(2)_O3_, with an occupation number ranging from 0.9603 (0.9637) to 0.9613 (0.9645) a.u. at the B3LYP (M06-2X) method. This contributes to enhanced stabilization interactions by providing a nearly pure *p*-type lone-pair orbital for electron donation in the LP(1)_O3_ → σ^*^_N1–C2_ interaction. The lone-pair LP(1)_O3_ occupies a higher-energy orbital (~ 0.9912 a.u.) with a 24.4% *p*-character at both DFT methods, making electrons readily available for interactions. The LP(*n*) → σ^*^ interaction, particularly between the LP(2)_O3_ nonbonding orbital and σ^*^_N1–C4_ antibonding orbitals, results in a higher stabilization energy of 4.99 (5.92) kcal mol^−1^, thereby enhancing intramolecular charge transfer and system stabilization. Similarly, intramolecular hyperconjugative interactions LP(1)_O3_ → σ^*^_N1–C2_ also occur, yielding a stabilization energy of 1.66 (1.91) kcal mol^−1^ at the B3LYP (M06-2X) method.

The NBO analysis of TEMPO derivatives protonation at oxygen and nitrogen sites was performed using the same theoretical levels, and the results are presented in Tables S11–S14 in the Supporting Information. As previously mentioned, the molecules under investigation possess two atoms with lone pairs capable of accepting electrophilic protons. However, protonation at the N-site leads to the cleavage of the C–N bond. These protonation events at different atoms induce alterations in the LP(1)_O3_ → σ^*^_N1–C2_ interactions. In the case of O-protonation, this results in a bond stabilization of 3.05 (3.62) kcal/mol, along with an energy increase of 1.39 (1.71) kcal/mol at the B3LYP (M06-2X) method (refer to Tables S11–S12 in the SI). Conversely, protonation at the N-site forms a π-bond between the N and O atoms, which is conjugated with the oxygen lone pair. The LP(1)_O3_ → σ^*^_N1–C2_ delocalization indicates the crucial role of oxygen atom delocalization in stabilizing TEMPO derivatives and O-protonated species.

The analysis of *E*_2_ values for the lone pair of electrons on the oxygen atom indicates their contribution to higher stabilization in O-protonated species, amounting to 3.05 (3.62) kcal/mol at the B3LYP (M06-2X) method (see Tables S11–S12 in the SI). Furthermore, there is additional stabilization from LP(2)_O3_ → σ^*^_C4–C10_ interactions in N-protonated forms, which is also observed in the studied TEMPO derivatives. The lower PA values may be attributed to a smaller stabilization due to the delocalization of the lone pair on the O-atom, as the LP(2)_O3_ → σ^*^_C4–C10_ interaction remains intact (see Tables S13–S14 in the SI). A comprehensive summary of all interactions and occupancies involving different atoms in the O-protonated and N-protonated considered nitroxide radicals is summarized in Tables S11–S14 in the SI.

The investigation reveals that the addition of a proton (H^+^) to an oxygen atom in the studied NO radicals reduces the lone pair occupancy [LP(1)_O3_] on the oxygen atom from 0.9912 (0.9913) to 0.9903 (0.9905) a.u. at the B3LYP (M06-2X) method, as depicted in Tables S11–S14 of the SI. Conversely, N-protonation of the studied molecules increases the lone pair occupancy, rising from 0.9912 (0.9913) to 0.9928 (0.9946) a.u. at the B3LYP (M06-2X) method. Notably, the occupancy of the second lone pair [LP(2)_O3_] decreases with N-protonation, shifting from 0.9611 (0.9645) to 0.9577 (0.9608) a.u., while the occupancy of the σ^*^_C4–C10_ antibonding orbital increases from 0.0153 (0.0151) to 0.0160 (0.0148) a.u. at the B3LYP (M06-2X) method. This alteration is attributed to electron repulsion from the added proton (H^+^) to the nitrogen atom, leading to the disruption of the *σ* bond between carbons C_2_ and C_5_.

### Frontier molecular orbital (FMO) analysis

Molecular orbitals, including energy, are crucial for physicists and chemists, and FMO analysis is a widely used technique to understand the optical and electronic properties of organic compounds. Understanding the HOMO and LUMO, along with their associated energies, is particularly valuable for assessing the chemical reactivity of molecules. The LUMO is an electron acceptor in molecular interactions, while the HOMO is an electron donor^64,75^. A high frontier orbital gap (ε_HOMO–LUMO_ gap) indicates high kinetic stability and low chemical reactivity^76–78^. The global electrophilicity index (*ω*) is calculated by^79,80^

3 $$\omega = {{\mu^{2} } \mathord{\left/ {\vphantom {{\mu^{2} } {2\eta }}} \right. \kern-0pt} {2\eta }}$$

where *η* is the global chemical hardness [*η* = (*E*_LUMO_ − *E*_HOMO_)/2] and *μ* represents the electronic chemical potential [*μ* = (*E*_HOMO_ + *E*_LUMO_)/2]^81^. Chemical hardness denotes a compound's ability to resist deformation^82^, while polarizability, or softness, is the inverse measure of resistance to deformation^83^ and is inversely related to hardness^84^. This study calculates HOMO and LUMO energies using the same theoretical level, focusing on chemical hardness and polarizability (see Table 5). Analysis of the table reveals that TEMPO is characterized as hard and more stable, indicating lower reactivity. Conversely, TEMPO-NO_2_ is identified as soft and the least stable in the gas phase, signifying higher reactivity. Additionally, absolute electronegativity (χ = − μ) serves as a measure of an atom's ability to attract shared electrons in a covalent bond to itself [χ = (*IP* + *EA*)/2]. Thus, TEMPO-NO_2_ exhibits higher electronegativity and greater charge flow.

**Table 5:** Calculated global reactivity descriptors for the studied TEMPO derivatives.

Substituent	Parameter										
	HOMO (eV)	LUMO (eV)	ΔE (eV)	*μ* (eV)	*η* (eV)	*ω* (eV)	*S* (eV)^−1^	χ (eV)	Δ*N*_max_	*IP* (eV)	*EA* (eV)
TEMPO	− 5.323	− 0.376	4.947	− 2.849	2.474	44.636	10.999	2.849	31.336	5.323	0.375
TEMPO-CH_3_	− 5.323	− 0.376	4.947	− 2.848	2.474	44.631	11.001	2.848	31.336	5.322	0.375
TEMPO-NH_2_	− 5.361	− 0.495	4.865	− 2.928	2.432	47.971	11.189	2.928	32.765	5.360	0.496
TEMPO-CHO	− 5.633	− 1.342	4.291	− 3.487	2.146	77.091	12.678	3.487	44.213	5.634	1.341
TEMPO-NO_2_	− 5.842	− 2.430	3.412	− 4.137	1.706	136.439	15.948	4.137	65.968	5.843	2.430

Table 5 shows that para-functional groups significantly affect the energy levels of the HOMO and LUMO orbitals at the B3LYP/6–311++ G(d,p) level. The EW groups like –CHO and –NO_2_ decrease frontier orbital energy levels, while the studied ED substitutes (CH_3_, NH_2_) have the opposite effect. Energy levels increase from the most strongly EW group (–NO_2_) to the most strongly ED group (–CH_3_). For instance, TEMPO-CH_3_ displays a HOMO energy of − 5.32 eV, while the nitro-substituted derivative has a HOMO energy of − 5.84 eV. The studied EW groups, by removing electrons, result in decreased HOMO and LUMO energies^85^. In contrast, the considered ED groups typically affect occupied nonbonding orbitals. The minimum E_HOMO–LUMO_ energy gap is observed with a –NO_2_ substituent, enhancing the reactivity of the piperidine ring, which has a six-membered structure with five methylene bridges (–CH_2_–) and one amine bridge (–NH–). Furthermore, a small ionization potential value, in conjunction with a low electron affinity, leads to high nucleophilicity and high electrophilicity, respectively. Thus, TEMPO-CH_3_ emerges as the most nucleophilic species, while TEMPO-NO_2_ exhibits strong electrophilicity. Δ*N*_max_, representing the maximum electronic charge (Δ*N*_max_ = − *μ*/*η*), and *S* denoting global softness (*S* = 1/*η*), are utilized to measure the direction of electron transfer. The study shows positive Δ*N*_max_ values, indicating their role as electron acceptors. Figure S2 (in the SI) illustrates the frontier molecular orbitals and energy levels of the examined NO radicals.

### Hydrogen bonding interaction in the studied nitroxide radicals

**Figure 3:**
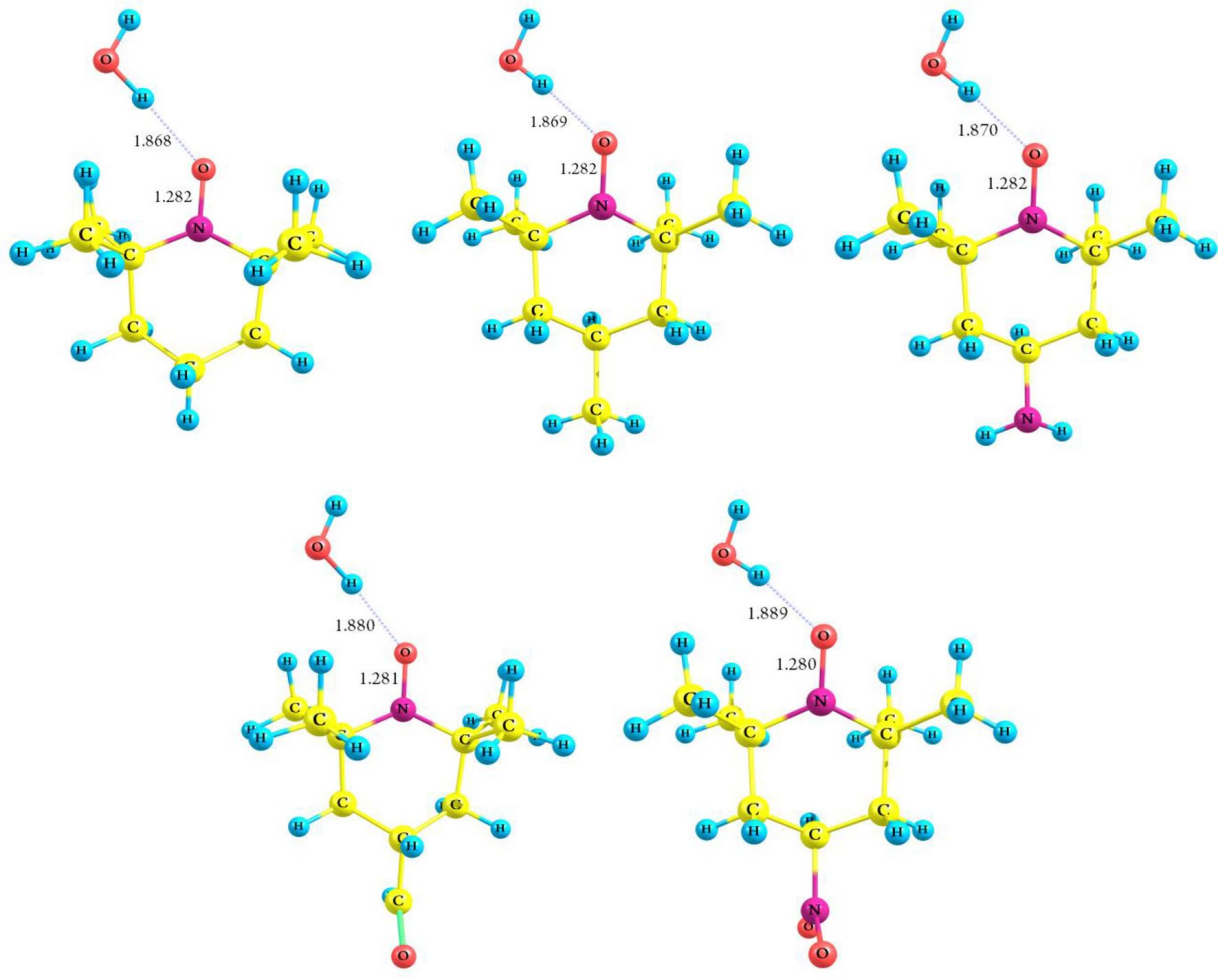
Optimized geometries of the most stable of the considered TEMPO derivatives with water molecules. The distance parameters *d*_1_ (N–O bond) and *d*_2_ (between the hydrogen atom of the water molecule and the oxygen atom of the nitroxide moiety) are given in Å.

The hydrogen bond interactions of the investigated TEMPO derivatives (**M**) with water molecules were examined. Consistent with the preceding study^27^, three conformations (top, planar, and orthogonal) were examined for the studied TEMPO derivatives. Table 6 provides the NO…H distances (*d*_2_ distances) and the corresponding calculated interaction energy values (*E*_int_) for each complex at the B3LYP/6–311++ G(d,p) level. The findings reveal that the planar conformation is more stable compared to the other conformers. Thus, Fig. 3 shows all the planar conformers of the TEMPO derivatives.

**Table 6:** NO…H distances (*d*_2_) and interaction energies (*E*_int_) of various H-bonded complexes.

Structure	Parameter			
	M + H_2_O $\leftrightarrows$ M(H_2_O)			
	Geometry	*d*_2_ (Å)	*E*_NO-radicals_ (a.u.)	*E*_int_ (kJ/mol)
TEMPO-CH_3_	Planar	1.869	− 599.3379	− 26.620
	Top	1.883	− 599.3378	− 26.342
	Orthogonal	1.907	− 599.3375	− 25.444
TEMPO	Planar	1.868	− 560.0399	− 26.672
	Top	1.884	− 560.0394	− 25.336
	Orthogonal	1.912	− 560.0398	− 26.176
TEMPO-NO_2_	Planar	1.889	− 764.6008	− 24.577
	Top	1.905	− 764.6007	− 24.331
	Orthogonal	1.943	− 764.6001	− 22.661
TEMPO-NH_2_	Planar	1.870	− 615.3904	− 26.612
	Top	1.886	− 615.3902	− 26.147
	Orthogonal	1.910	− 615.3899	− 25.213
TEMPO-CHO	Planar	1.880	− 673.3798	− 25.717
	Top	1.898	− 673.3795	− 24.974
	Orthogonal	1.924	− 673.3795	− 24.940

As indicated in Table 6, the distance of *d*_2_ shows an increase for both the examined ED group (–CH_3_, –NH_2_) and the EW group (–CHO, –NO_2_) when compared to TEMPO radicals. The lowest *E*_int_ value (− 24.58 kJ/mol), suggesting a weak H-bonding interaction with a distance of 1.8892 Å, was observed in a –NO_2_ substitution complex, while the strongest interaction with a distance of 1.8683 Å was observed in TEMPO (*E*_int_ = − 26.67 kJ/mol). The trend suggests that a shorter distance *d*_2_ corresponds to a larger *E*_int_, indicating a stronger H-bonded complex. The higher *E*_int_ values observed in complexes containing CH_3_-substituted nitroxide radicals are due to the electron-donating inductive effects of the –CH_3_ group, while stronger H-bonds are observed in unsubstituted species (TEMPO) due to steric hindrance caused by CH_3_ groups (see Fig. 3).

**Figure 4:**
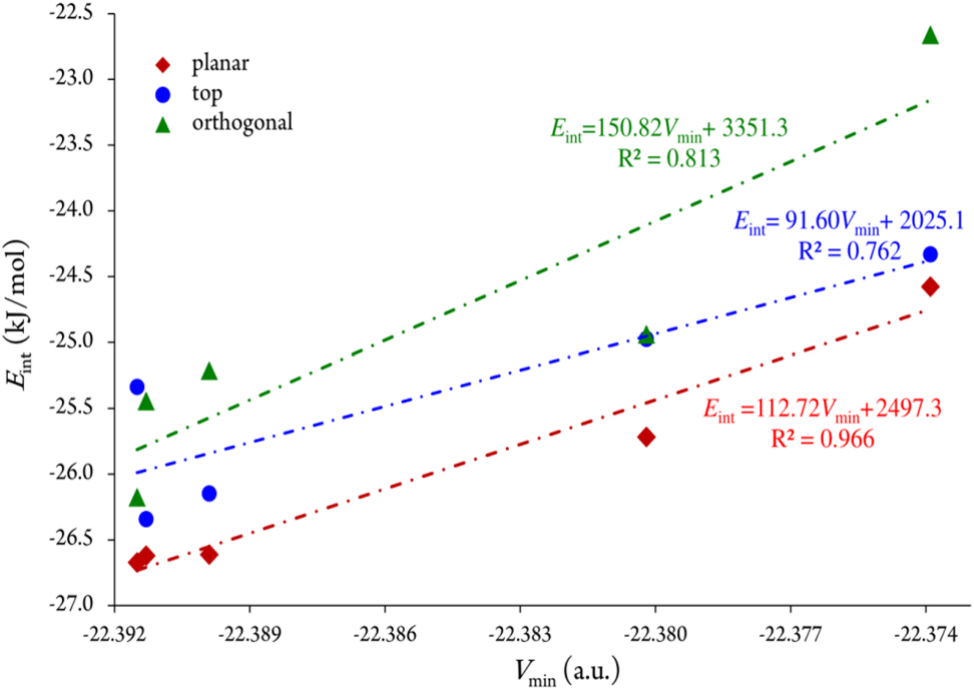
Correlation between the interaction energy *E*_int_ and *V*_min_ of the studied NO…H_2_O complexes.

Figure 4 depicts the correlation between the *V*_min_ values of oxygen in the nitroxide moiety of compounds and the corresponding *E*_int_ for the related hydrogen-bonded complexes. A high correlation, characterized by a correlation coefficient of 0.966, is observed for the *planar* form, indicating a strong relationship. The *orthogonal* and *top* forms display moderately good correlations, with coefficients of 0.813 and 0.762, respectively. The observed correlation between *V*_min_ and *E*_int_ implies that *V*_min_ values reflect the electronic impact of TEMPO derivatives, making them a suitable descriptor for evaluating the strength of hydrogen bond interactions in this context.

We performed an additional analysis of the spin density redistribution in the NO moieties resulting from hydrogen bonding interactions in unsubstituted NO…H_2_O complexes. Across all complexes, the existence of non-zero spin densities on H_2_O fragments confirms the partial spin transfer from the studied molecules. Table 7 shows that the computed electron spin density on the nitrogen atom slightly increases in H_2_O complexes, indicating a transfer of spin density from the oxygen atom to the nitrogen atom, while the overall spin density remains nearly constant^86^.

**Table 7:** Spin densities of the nitroxide moiety for the considered TEMPO derivatives and the hydrated forms at the B3LYP/6–311 +  + G(d,p) level.

Species	Mode							
	Natural		Hydrated of the NO radicals					
			Planar		Top		Orthogonal	
	N	O	N	O	N	O	N	O
TEMPO-CH_3_	0.4591	0.5190	0.4820	0.4964	0.4993	0.4609	0.4990	0.4616
TEMPO	0.4545	0.5213	0.4822	0.4978	0.4943	0.4627	0.4955	0.4630
TEMPO-NH_2_	0.4495	0.5229	0.4768	0.4984	0.4914	0.4643	0.4945	0.4620
TEMPO-CHO	0.4549	0.5234	0.4789	0.5019	0.4976	0.4657	0.4984	0.4653
TEMPO-NO_2_	0.4379	0.5311	0.4673	0.5061	0.4811	0.4737	0.4783	0.4718

### Characterization of chemical bonds

The Atoms in Molecules (AIM) method was used to analyze intermolecular hydrogen bond interactions using the electron density function [ρ(r)] and Laplacian [∇^2^ρ(r)], which is crucial in understanding the nature of chemical bonding^47^. The ∇^2^*ρ*(r) value at the BCP indicates electron density concentration, distinguishing between shared-shell and closed-shell bonding interactions. Additionally, the ρ(*r*) value is linked to the hydrogen bond energy, E_HB_. According to Espinosa,^87^ for H-bond energy (*E*) and potential energy density (V_BCP_) at the H…O contact is expressed as E_HB_ = V(*r*)/2. Besides, ρ(*r*) and ∇^2^*ρ*(*r*), kinetic energy density [G(*r*)], potential energy density [V(*r*)], E_HB_, and |V(*r*)|/G(*r*) are effective parameters for characterizing hydrogen bonding. The V(*r*) value correlates with the E_HB_^88^, and the ratio, |V(r)|/G(r), indicates the type of interaction, with values greater than 2 for covalent bonds, between 1 and 2 for mixed-character interactions, and lower than 1 for the ionic, H-bond and van der Waals interactions^89^.

The presence of a (3, − 1) bond critical point for the “proton (H)…acceptor (A)” contact confirms the existence of the H-bonding interaction^90^. In the general context, it is commonly recognized that the electron density (*ρ*_H…A_) value for a typical H-bond should fall within the 0.002–0.040 a.u. range and the Laplacian ∇^2^*ρ*(*r*_BCP_) value should be between 0.024 and 0.139 a.u., respectively^91,92^. The strength of H-bond interactions at the BCP can be classified into three types^93^: (*a*) strong H-bonds are characterized by ∇^2^ρ(*r*) < 0 and H(*r*) < 0, exhibiting covalent characteristics; (*b*) medium H-bonds are considered by ∇^2^ρ(*r*) > 0 and H(*r*) < 0, showing partial covalency; and (*c*) weak H-bonds are categorized by ∇^2^ρ(*r*) > 0 and H(*r*) > 0, typically displaying electrostatic properties, with the distance between interacting atoms exceeding the sum of their van der Waals radii.

The investigation assesses the hydrogen bonds formed in NO…H_2_O complexes by analyzing AIM parameters at the BCP of the NO…H bond across all examined conformers. The ρ(*r*) value at the BCP is considered indicative of the strength of hydrogen bonding interactions^43^. The study reveals that the strongest and most stable TEMPO derivative is the “planar” conformer, which forms the NO…H_2_O bond. The total electron density H(r) values can provide valuable insights into the nature of a chemical bond [H(*r*) = G(*r*) + V(*r*)]. Notably, all investigated nitroxide complexes displayed negative H(*r*) values within the range of (− 0.0021)–(− 0.0027) a.u. Thus, the *ρ*(*r*) value decreases with NO_2_ substitution, indicating a reduction in the strength of the H-bonding interaction compared to X = H or CH_3_ complexes.

The AIM parameters for hydration reactions involving all nitroxide radicals were computed at the BCP(3, − 1), with the results detailed in Table 8. The molecular graph for all conformers (top, planar, and orthogonal) of the investigated TEMPO derivatives is depicted in Fig. S4 in the Supplementary Information. Tables 6 and 8 present geometrical and topological factors governing H-bonds among atoms, categorizing interactions in molecular graphs based on their geometrical, topological, and energetic properties. As indicated in Table 8, the ratio |V(*r*)|/G(*r*) for the N–O…H_2_O interaction is found to be less than unity, indicating the noncovalent character of the N–O…H_2_O bond. The calculated interaction energy at the BCP indicates strong H-bonds with a covalent character. The AIM results suggest that the stability of TEMPO derivative inclusion complexes is significantly influenced by intermolecular hydrogen interactions.

**Table 8:** Topological parameters computed at the BCP(3, − 1) for the N–O…H interaction in the TEMPO derivatives at the B3LYP/6–311++ G(d,p) level. All the parameters are in a.u., except for E_HB_ (in kcal/mol).

Species	Parameter							
	Geometry	Hydrated form						
		*ρ*(*r*)	∇^2^*ρ*(*r*)	G(*r*)	H(*r*)	V(*r*)	|V(*r*)|/G(*r*)	E_HB_
TEMPO	Planar	0.0279	− 0.0263	0.0241	− 0.0021	0.0220	0.9129	6.903
	Top	0.0259	− 0.0252	0.0226	− 0.0025	0.0200	0.8850	6.275
	Orthogonal	0.0247	− 0.0235	0.0210	− 0.0025	0.0185	0.8810	5.804
TEMPO-CH_3_	Planar	0.0278	− 0.0262	0.0241	− 0.0021	0.0220	0.9129	6.903
	Top	0.0259	− 0.0251	0.0225	− 0.0025	0.0200	0.8889	6.275
	Orthogonal	0.0245	− 0.0233	0.0208	− 0.0025	0.0183	0.8798	5.742
TEMPO-NH_2_	Planar	0.0277	− 0.0261	0.0240	− 0.0021	0.0219	0.9125	6.871
	Top	0.0257	− 0.0249	0.0224	− 0.0026	0.0198	0.8839	6.212
	Orthogonal	0.0277	− 0.0261	0.0240	− 0.0021	0.0219	0.9125	6.871
TEMPO-CHO	Planar	0.0270	− 0.0256	0.0234	− 0.0022	0.0211	0.9017	6.620
	Top	0.0253	− 0.0245	0.0219	− 0.0026	0.0193	0.8813	6.055
	Orthogonal	0.0237	− 0.0225	0.0199	− 0.0025	0.0174	0.8744	5.459
TEMPO-NO_2_	Planar	0.0265	− 0.0252	0.0229	− 0.0023	0.0206	0.8996	6.463
	Top	0.0247	− 0.0241	0.0214	− 0.0027	0.0188	0.8785	5.899
	Orthogonal	0.0230	− 0.0219	0.0194	− 0.0026	0.0168	0.8660	5.271

As can be seen from Table 8, planar configurations consistently exhibit the highest E_HB_ values among the different configurations for each TEMPO derivative. This suggests stronger hydrogen bonding interactions between the N–O and hydrogen atoms in the studied molecules compared to the other conformers (top and orthogonal). These values provide insights into the stability and strength of hydrogen bonding within the molecules studied.

## Conclusion

A density functional theory (DFT) computational investigation was carried out using B3LYP and M06-2X functionals in association with a 6–311++ G(d,p) basis set to verify the level of theory. Frequency calculations were performed at the same level of theory to obtain the thermodynamic data of the optimized structures on the global minimum of their potential energy surfaces. The primary objective of this study was to explore the molecular structural characteristics, thermodynamic parameters, natural bond order (NBO) analysis, the molecular electrostatic potential (MEP), and hydrogen bond interactions for the studied TEMPO derivatives (X: –H, –CH_3_, –NH_2_, –CHO, and –NO_2_). The study explored NBO to analyze intermolecular interactions and stabilization energies, providing insights into charge distributions within molecules. Furthermore, the quantum theory of atoms in molecules (QTAIM) was used to investigate topological parameters at the bond critical point.

The LUMO–HOMO energy gap (E_H–L_) values indicate a molecule's chemical activity, with a reduction indicating charge transfer interactions. The frontier molecular orbital (FMO) analysis indicates that TEMPO ≈ TEMPO-CH_3_ and TEMPO-NO_2_ have the highest and lowest stabilities in the gas phase, respectively. Furthermore, intramolecular charge transfer contributes to molecule stabilization, and the computed E_H–L_ energy gap aligns with intramolecular hyperconjugative interactions [LP(e) → σ^*^].

The proton affinities (PAs) and associated thermochemical parameters of the examined TEMPO derivatives were assessed by a comprehensive entropy analysis. The PA for these nitroxide radicals were determined as follows: 894.17 (885.45) kJ/mol (TEMPO), 896.04 (887.35) kJ/mol (TEMPO-CH_3_), 892.57 (883.83) kJ/mol (TEMPO-NH_2_), 870.36 (861.20) kJ/mol (TEMPO-CHO), and 851.29 (842.14) kJ/mol (TEMPO-NO_2_). Gas-phase basicities (GBs) and protonation entropies (Δ_p_*S*) for these radicals were also calculated accordingly. The GB values are notably lower than the PA values, mostly due to the Gibbs free energy of the proton (− 6.30 kJ/mol). The six-membered cyclic nitroxides are identified as robust super- and hyperbases, with computed PAs well above the threshold for super-basicity.

Furthermore, it is essential to address the discrepancy observed in the HOMO–LUMO gap behavior between the O-protonated and N-protonated forms at the B3LYP and M06-2X methods. The B3LYP method exhibits an increase in the HOMO–LUMO gap for the O-protonated form and a decrease for the N-protonated form, while the M06-2X method shows an increase in the HOMO–LUMO gap for both protonated forms. These methodological discrepancies result in contrasting behaviors in the HOMO–LUMO gap, depending on the protonation site. Moreover, it is worth noting that the observed changes in the HOMO–LUMO gap reflect alterations in the molecular orbital energies and electronic interactions induced by protonation. The presence of the proton disrupts the original electronic structure of the TEMPO derivatives, leading to variations in orbital energies and overall stability. These changes highlight the sensitivity of the HOMO–LUMO gap to protonation and emphasize the importance of selecting an appropriate computational method for accurate predictions.

The AIM methodology was employed to investigate the factors influencing conformational preference and hydrogen bond strength. The negative values of the Laplacian of electron density [∇^2^ρ(*r*) < 0] and total electron density [H(*r*) < 0] in the N–O…H interaction indicate a partially covalent nature of bonding. AIM analysis demonstrates that in H_2_O complexes, the N–O…H interaction exhibits the strongest hydrogen bond strength. Electronic structure characterization of hydrogen bonds through AIM analysis for the more stable conformers of the examined TEMPO derivatives (in the planar mode) revealed N–O…H bonds. These bonds are characterized by hydrogen bond energies of 6.90, 6.90, 6.87, 6.62, and 6.46 kcal/mol for –H, –CH_3_, –NH_2_, –CHO, and –NO_2_ substitution, respectively, all computed at bond critical points (3, − 1). Finally, the findings revealed that in the hydration of TEMPO derivatives, the planar configurations consistently exhibit the highest E_HB_ values for each TEMPO derivative compared to the top and orthogonal conformers. This suggests stronger hydrogen bonding interactions between the N–O and hydrogen atoms of water molecules in the planar conformer.

### Supplementary Information

Supplementary Information.

## Data Availability

All data generated through this study are collected in this manuscript and the Supporting Information file.
