# Supplementary material for: Computational analysis of substituent effects on proton affinity and gas-phase basicity of TEMPO derivatives and their hydrogen bonding interactions with water molecules
Source: Sci Rep. 2024 Apr 10;14:8434. doi: 10.1038/s41598-024-58582-x (PMC11006853; doi:10.1038/s41598-024-58582-x)
Supplement: Supplementary file 1 — Supplementary Information. [file 41598_2024_58582_MOESM1_ESM.docx]

## Computational analysis of substituent effects on the proton affinity and gas-phase basicity of TEMPO derivatives and their hydrogen bonding interactions with water molecules

Abolfazl Shiroudi^[[1]](#footnote-1)^*^1,2🖂^, Maciej Śmiechowski^1^, Jacek Czub^1,2^

& Mohamed A. Abdel-Rahman^3🖂^

^a^ Department of Physical Chemistry, Gdańsk University of Technology, Narutowicza 11/12, Gdańsk 80-233, Poland

^b^ BioTechMed Center, Gdańsk University of Technology, Gdańsk 80-233, Poland

^c^ Chemistry Department, Faculty of Science, Suez University, Suez 43518, Egypt

**Supplementary Information**

**Table S1**. Cartesian coordinates of TEMPO derivatives and the protonated forms were optimized using the considered DFT methods, along with the split valence 6-311++G(d,p) basis set.

| **TEMPO** [optimized at the B3LYP/6-311++G(d,p) level]  0 2  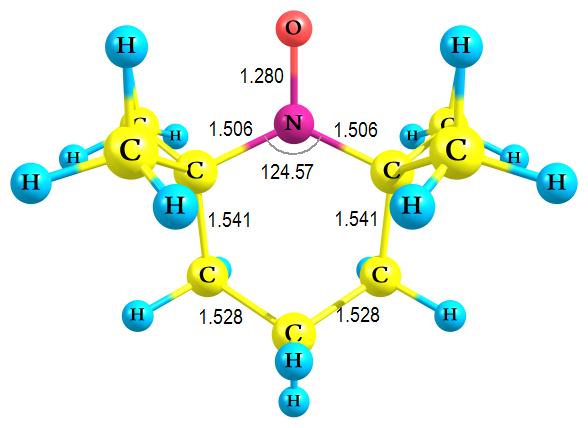 N -0.75060800 0.19830700 0.00000000  C -0.07133400 0.02719900 1.33348300  O -2.02276700 0.05555300 0.00000000  C -0.07133400 0.02719900 -1.33348300  C 1.39603100 0.48972100 1.24494700  C -0.17248400 -1.45024700 1.76608300  C -0.82457400 0.90408700 2.34514200  C -0.82457400 0.90408700 -2.34514200  C -0.17248400 -1.45024700 -1.76608300  C 1.39603100 0.48972100 -1.24494700  C 2.12496400 -0.01303100 0.00000000  H 1.90677900 0.16743200 2.15767900  H 1.41924800 1.58573200 1.24329700  H 0.17409400 -1.55724700 2.79772600  H 0.43075100 -2.10918500 1.13912600  H -1.21158400 -1.77966700 1.71363200  H -0.33014700 0.84126900 3.31841500  H -1.85809500 0.57664200 2.45111700  H -0.82661100 1.94871400 2.02464000  H -0.33014700 0.84126900 -3.31841500  H -0.82661100 1.94871400 -2.02464000  H -1.85809500 0.57664200 -2.45111700  H 0.17409400 -1.55724700 -2.79772600  H -1.21158400 -1.77966700 -1.71363200  H 0.43075100 -2.10918500 -1.13912600  H 1.90677900 0.16743200 -2.15767900  H 1.41924800 1.58573200 -1.24329700  H 2.19054800 -1.10592700 0.00000000  H 3.15552800 0.35504500 0.00000000  Zero-point correction= 0.260688 (Hartree/Particle)  Thermal correction to Energy= 0.272734  Thermal correction to Enthalpy= 0.273678  Thermal correction to Gibbs Free Energy= 0.223984  Sum of electronic and zero-point Energies= -483.595372  Sum of electronic and thermal Energies= -483.583326  Sum of electronic and thermal Enthalpies= -483.582382  Sum of electronic and thermal Free Energies= -483.632076  **TEMPO-CH_3_** [optimized at the B3LYP/6-311++G(d,p) level]  0 2  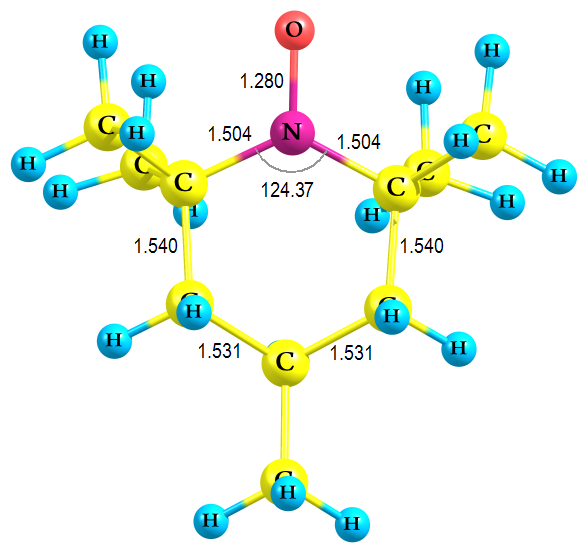 N -1.05578700 0.00000000 -0.24482300  C -0.39204100 1.33034800 -0.01651800  O -2.33559700 0.00000000 -0.21419000  C -0.39204100 -1.33034800 -0.01651800  C 1.10957300 1.23877900 -0.34726700  C -0.62475600 1.76808300 1.44481700  C -1.06172100 2.34216100 -0.95917400  C -1.06172000 -2.34216100 -0.95917400  C -0.62475500 -1.76808300 1.44481700  C 1.10957300 -1.23877900 -0.34726700  C 1.81303100 0.00000000 0.21417000  H 1.58946900 2.15327700 0.01712700  H 1.23200100 1.23619500 -1.43771900  H -0.28305700 2.79804400 1.58038000  H -0.08792800 1.13965300 2.15753900  H -1.68988700 1.72289400 1.67826100  H -0.57578400 3.31576100 -0.85165000  H -2.12094700 2.44724500 -0.72722800  H -0.96822800 2.02134700 -1.99950600  H -0.57578300 -3.31576100 -0.85165000  H -0.96822800 -2.02134700 -1.99950600  H -2.12094600 -2.44724500 -0.72722800  H -0.28305700 -2.79804400 1.58038000  H -1.68988600 -1.72289400 1.67826100  H -0.08792800 -1.13965300 2.15753900  H 1.58946900 -2.15327700 0.01712700  H 1.23200100 -1.23619500 -1.43771900  H 1.72462900 0.00000000 1.30711800  C 3.30626100 0.00000000 -0.13199400  H 3.80716300 0.88395000 0.27359900  H 3.80716300 -0.88394900 0.27359900  H 3.45662900 0.00000000 -1.21680800  Zero-point correction= 0.288239 (Hartree/Particle)  Thermal correction to Energy= 0.301845  Thermal correction to Enthalpy= 0.302789  Thermal correction to Gibbs Free Energy= 0.249797  Sum of electronic and zero-point Energies= -522.893395  Sum of electronic and thermal Energies= -522.879790  Sum of electronic and thermal Enthalpies= -522.878846  Sum of electronic and thermal Free Energies= -522.931838  **TEMPO-NH_2_** [optimized at the B3LYP/6-311++G(d,p) level]  0 2  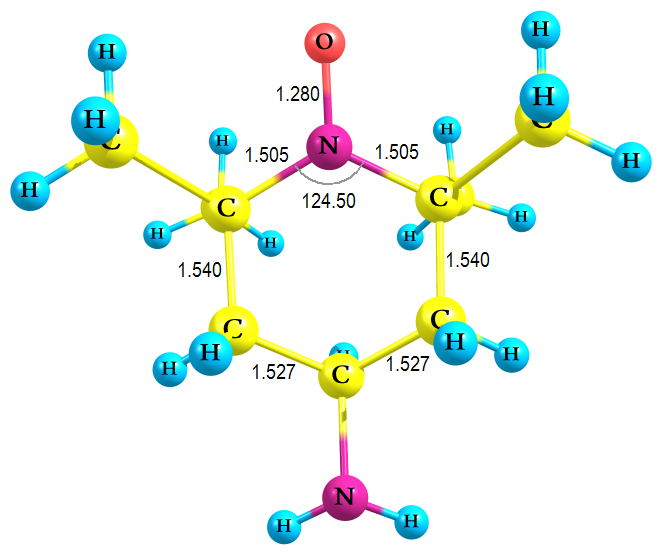 N -1.04624400 0.00000000 -0.23345000  C -0.37962600 1.33154500 -0.01786800  O -2.32525100 0.00000000 -0.18474900  C -0.37962600 -1.33154500 -0.01786800  C 1.11795100 1.23678200 -0.36224500  C -0.60507100 1.77885600 1.44201300  C -1.05451200 2.33759600 -0.96310800  C -1.05451200 -2.33759500 -0.96310800  C -0.60507100 -1.77885600 1.44201300  C 1.11795000 -1.23678300 -0.36224500  C 1.81397900 0.00000000 0.20175500  H 1.60529000 2.14939700 -0.00091600  H 1.24640200 1.22002900 -1.44962300  H -0.25121900 2.80537400 1.57259800  H -0.07576100 1.14709100 2.15749600  H -1.67054100 1.74752900 1.67618300  H -0.57051300 3.31286400 -0.86257200  H -2.11283600 2.44092900 -0.72613800  H -0.96432400 2.01179600 -2.00200500  H -0.57051500 -3.31286400 -0.86257200  H -0.96432500 -2.01179500 -2.00200500  H -2.11283700 -2.44092800 -0.72613900  H -0.25122100 -2.80537400 1.57259800  H -1.67054100 -1.74752900 1.67618300  H -0.07576100 -1.14709100 2.15749600  H 1.60529000 -2.14939800 -0.00091600  H 1.24640200 -1.22002900 -1.44962300  H 1.72248300 0.00000000 1.29817200  N 3.22105400 -0.00000100 -0.22543200  H 3.70704400 0.81665700 0.13296300  H 3.70704400 -0.81665900 0.13296200  Zero-point correction= 0.277599 (Hartree/Particle)  Thermal correction to Energy= 0.291064  Thermal correction to Enthalpy= 0.292008  Thermal correction to Gibbs Free Energy= 0.239309  Sum of electronic and zero-point Energies= -538.945849  Sum of electronic and thermal Energies= -538.932384  Sum of electronic and thermal Enthalpies= -538.931440  Sum of electronic and thermal Free Energies= -538.984140  **TEMPO-CHO** [optimized at the B3LYP/6-311++G(d,p) level]  0 2  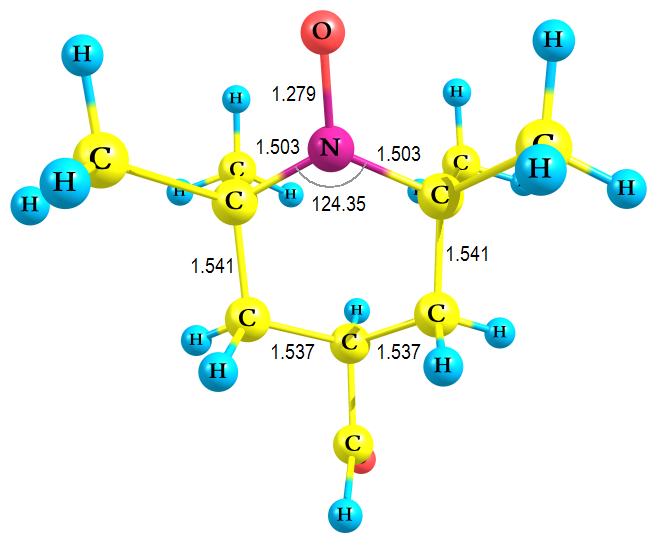 N 1.32243900 0.00004500 -0.21406700  C 0.64994800 -1.32960600 -0.01381500  O 2.59814400 0.00010300 -0.11723000  C 0.64982800 1.32963700 -0.01382100  C -0.82924900 -1.24621700 -0.43914600  C 0.78986700 -1.75815700 1.46143500  C 1.37327100 -2.34836800 -0.90817600  C 1.37305800 2.34846000 -0.90818700  C 0.78970800 1.75820600 1.46142700  C -0.82936200 1.24611200 -0.43915100  C -1.55697400 -0.00008600 0.08859500  H -1.32957500 -2.15782900 -0.09893500  H -0.88316000 -1.24389200 -1.53439500  H 0.44055100 -2.78715000 1.58200500  H 0.21138500 -1.12640400 2.13718600  H 1.83871600 -1.71173200 1.75890600  H 0.88042300 -3.32063300 -0.82450700  H 2.41525900 -2.45338500 -0.60900100  H 1.34701200 -2.03412200 -1.95424500  H 0.88012600 3.32068300 -0.82451900  H 1.34682400 2.03420900 -1.95425500  H 2.41503900 2.45356900 -0.60901500  H 0.44029400 2.78716700 1.58199400  H 1.83856200 1.71188300 1.75889600  H 0.21128800 1.12640000 2.13718100  H -1.32977100 2.15767800 -0.09894200  H -0.88327300 1.24377700 -1.53440100  H -1.59541700 -0.00008300 1.18040800  C -2.98970200 -0.00014000 -0.39866000  H -3.10543700 -0.00012300 -1.50690300  O -3.96292800 -0.00002600 0.31322900  Zero-point correction= 0.269533 (Hartree/Particle)  Thermal correction to Energy= 0.283797  Thermal correction to Enthalpy= 0.284741  Thermal correction to Gibbs Free Energy= 0.229215  Sum of electronic and zero-point Energies= -596.935545  Sum of electronic and thermal Energies= -596.921281  Sum of electronic and thermal Enthalpies= -596.920337  Sum of electronic and thermal Free Energies= -596.975863  **TEMPO-NO_2_** [optimized at the B3LYP/6-311++G(d,p) level]  0 2  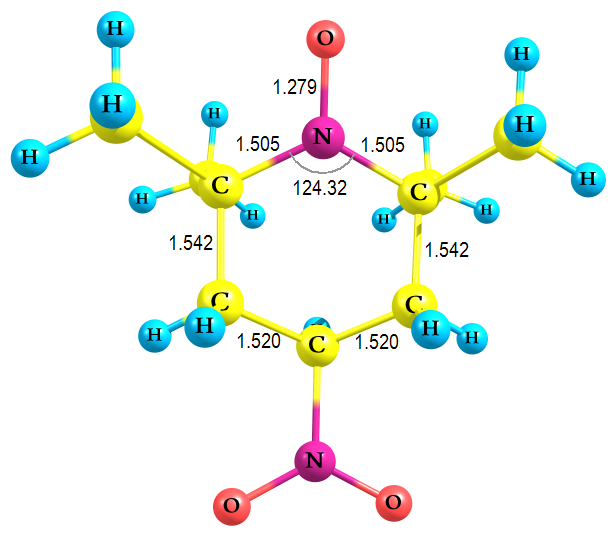 N -1.54002100 0.00000200 -0.25998000  C -0.88096800 1.33047000 -0.01618700  O -2.81887500 0.00000700 -0.24638100  C -0.88097700 -1.33046700 -0.01616300  C 0.62574700 1.24850100 -0.33443800  C -1.12923100 1.76660400 1.44272500  C -1.53279500 2.34609400 -0.96662500  C -1.53281200 -2.34610300 -0.96658400  C -1.12924400 -1.76657300 1.44275500  C 0.62573800 -1.24851500 -0.33441600  C 1.26939000 -0.00000500 0.24740400  H 1.11223800 2.14819900 0.04187700  H 0.76648100 1.23525700 -1.41995900  H -0.78704100 2.79503500 1.58413000  H -0.60741500 1.13754500 2.16664600  H -2.19756400 1.72315800 1.66008000  H -1.04854000 3.31889000 -0.84908600  H -2.59464200 2.44950800 -0.74761300  H -1.42573200 2.02768800 -2.00613400  H -1.04856300 -3.31890000 -0.84902900  H -1.42574900 -2.02771500 -2.00609800  H -2.59465900 -2.44950600 -0.74756900  H -0.78706200 -2.79500400 1.58417900  H -2.19757600 -1.72311500 1.66011000  H -0.60742300 -1.13750600 2.16666600  H 1.11222200 -2.14821100 0.04191400  H 0.76647400 -1.23529200 -1.41993700  H 1.22347900 0.00000600 1.34017000  N 2.78775300 -0.00000600 -0.01023400  O 3.34679300 -1.08253000 -0.08311400  O 3.34681400 1.08251800 -0.08296500  Zero-point correction= 0.263223 (Hartree/Particle)  Thermal correction to Energy= 0.277079  Thermal correction to Enthalpy= 0.278024  Thermal correction to Gibbs Free Energy= 0.223661  Sum of electronic and zero-point Energies= -688.155840  Sum of electronic and thermal Energies= -688.141983  Sum of electronic and thermal Enthalpies= -688.141039  Sum of electronic and thermal Free Energies= -688.195401  **TEMPO** [optimized at the M06-2X/6-311++G(d,p) level]  0 2  N -0.74430500 0.22471500 0.00000000  C -0.07094900 0.02588100 1.31741500  O -2.00754200 0.09240700 0.00000000  C -0.07094900 0.02588100 -1.31741500  C 1.38300200 0.50527700 1.24006500  C -0.15721000 -1.45612000 1.71012100  C -0.83184500 0.86392700 2.34507500  C -0.83184500 0.86392700 -2.34507500  C -0.15721000 -1.45612000 -1.71012100  C 1.38300200 0.50527700 -1.24006500  C 2.11612500 0.00714200 0.00000000  H 1.88988000 0.18629100 2.15540800  H 1.38939100 1.60124500 1.23435400  H 0.14877100 -1.57498400 2.75244600  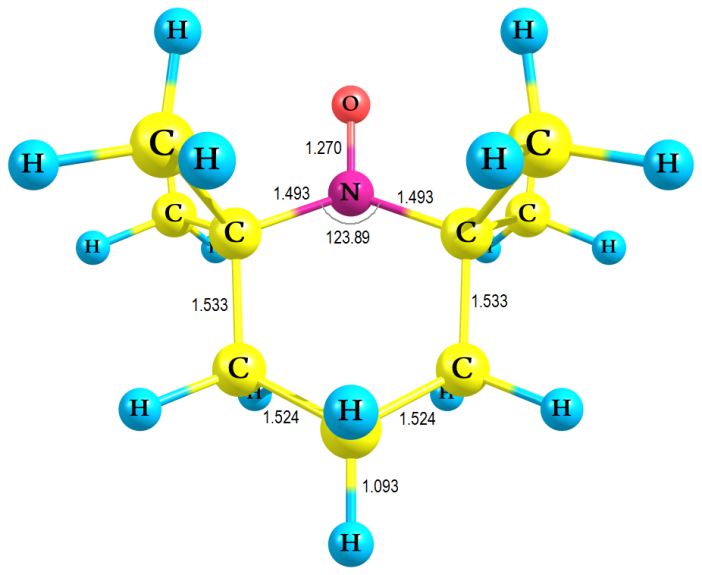 H 0.48490300 -2.08781200 1.09523900  H -1.18845400 -1.79874300 1.60739400  H -0.31074800 0.81332400 3.30414000  H -1.84867100 0.49458700 2.46991700  H -0.87923400 1.90669200 2.02462400  H -0.31074800 0.81332400 -3.30414000  H -0.87923400 1.90669200 -2.02462400  H -1.84867100 0.49458700 -2.46991700  H 0.14877100 -1.57498400 -2.75244600  H -1.18845400 -1.79874300 -1.60739400  H 0.48490300 -2.08781200 -1.09523900  H 1.88988000 0.18629100 -2.15540800  H 1.38939100 1.60124500 -1.23435400  H 2.18331400 -1.08491400 0.00000000  H 3.14276600 0.38103200 0.00000000  Zero-point correction= 0.264118 (Hartree/Particle)  Thermal correction to Energy= 0.275845  Thermal correction to Enthalpy= 0.276789  Thermal correction to Gibbs Free Energy= 0.227810  Sum of electronic and zero-point Energies= -483.366967  Sum of electronic and thermal Energies= -483.355240  Sum of electronic and thermal Enthalpies= -483.354296  Sum of electronic and thermal Free Energies= -483.403275  **TEMPO-CH_3_** [optimized at the M06-2X/6-311++G(d,p) level]  0 2  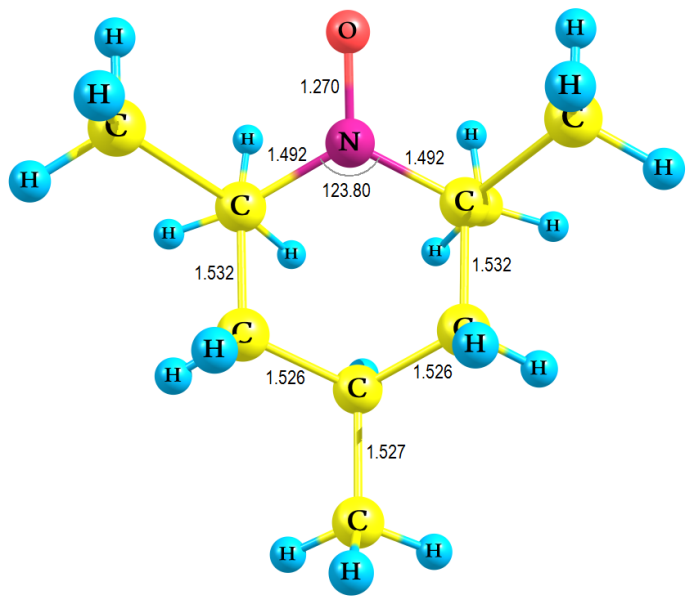 N 1.04212100 0.00000000 -0.27417000  C 0.38924500 -1.31583500 -0.01440400  O 2.31222900 0.00000000 -0.26306100  C 0.38924500 1.31583500 -0.01440400  C -1.10360800 -1.23398400 -0.34830500  C 0.62168000 -1.71500700 1.45026400  C 1.06160600 -2.34200000 -0.92716500  C 1.06160600 2.34200000 -0.92716500  C 0.62168000 1.71500700 1.45026400  C -1.10360800 1.2 3398400 -0.34830500  C -1.80310700 0.00000000 0.21437500  H -1.58078500 -2.14948900 0.01688000  H -1.22180600 -1.22560500 -1.43939300  H 0.32068700 -2.75531700 1.59639200  H 0.05209600 -1.09735600 2.14556900  H 1.68317800 -1.62174300 1.68674200  H 0.54838600 -3.30135200 -0.82681900  H 2.11041500 -2.46696500 -0.66172500  H 1.00401200 -2.01903200 -1.96863200  H 0.54838600 3.30135200 -0.82681900  H 1.00401200 2.01903200 -1.96863200  H 2.11041500 2.46696600 -0.66172500  H 0.32068700 2.75531700 1.59639200  H 1.68317800 1.62174300 1.68674200  H 0.05209600 1.09735600 2.14556900  H -1.58078500 2.14948900 0.01688000  H -1.22180600 1.22560500 -1.43939300  H -1.71653000 0.00000000 1.30685000  C -3.28688200 0.00000000 -0.14473200  H -3.78921100 -0.88467200 0.25376200  H -3.78921100 0.88467200 0.25376200  H -3.41724100 0.00000000 -1.23126600  Zero-point correction= 0.291956 (Hartree/Particle)  Thermal correction to Energy= 0.305215  Thermal correction to Enthalpy= 0.306160  Thermal correction to Gibbs Free Energy= 0.253960  Sum of electronic and zero-point Energies= -522.645321  Sum of electronic and thermal Energies= -522.632062  Sum of electronic and thermal Enthalpies= -522.631117  Sum of electronic and thermal Free Energies= -522.683317  **TEMPO-NH_2_** [optimized at the M06-2X/6-311++G(d,p) level]  0 2  N 1.03406800 0.00000000 -0.26173700  C 0.37726800 -1.31685200 -0.01595900  O 2.30360300 0.00000000 -0.23079200  C 0.37726800 1.31685200 -0.01595900  C -1.11100700 -1.23174800 -0.36609900  C 0.60016000 -1.72594100 1.44778800  C 1.05556600 -2.33769700 -0.93046300  C 1.05556600 2.33769700 -0.93046300  C 0.60016000 1.72594100 1.44778800  C -1.11100700 1.23174 800 -0.36609900  C -1.80509100 0.00000000 0.19830500  H -1.59574000 -2.14568400 -0.00525200  H -1.23386600 -1.20839200 -1.45421100  H 0.28457400 -2.76265300 1.58862300  H 0.03887400 -1.10314500 2.14531200  H 1.66245700 -1.64864700 1.68623600  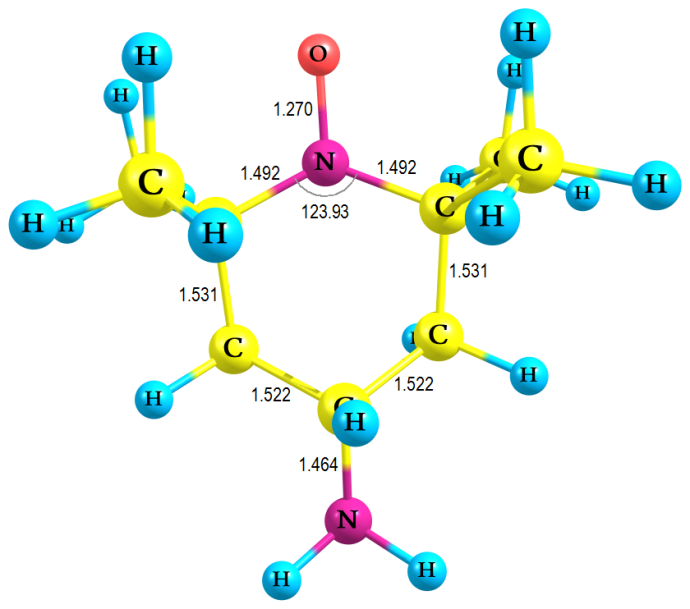 H 0.54440200 -3.29876300 -0.83687300  H 2.10325300 -2.46077400 -0.65972300  H 1.00150500 -2.01017100 -1.97051600  H 0.54440200 3.29876300 -0.83687300  H 1.00150500 2.01017000 -1.97051600  H 2.10325300 2.46077400 -0.65972300  H 0.28457400 2.76265300 1.58862300  H 1.66245700 1.64864700 1.68623600  H 0.03887400 1.10314500 2.14531200  H -1.59574000 2.14568400 -0.00525200  H -1.23386500 1.20839200 -1.45421100  H -1.71560600 0.00000000 1.29401300  N -3.20279100 0.00000000 -0.23696800  H -3.68818500 -0.81600800 0.12150400  H -3.68818500 0.81600800 0.12150400  Zero-point correction= 0.281329 (Hartree/Particle)  Thermal correction to Energy= 0.294448  Thermal correction to Enthalpy= 0.295392  Thermal correction to Gibbs Free Energy= 0.243472  Sum of electronic and zero-point Energies= -538.697552  Sum of electronic and thermal Energies= -538.684433  Sum of electronic and thermal Enthalpies= -538.683488  Sum of electronic and thermal Free Energies= -538.735409  **TEMPO-CHO** [optimized at the M06-2X/6-311++G(d,p) level]  0 2  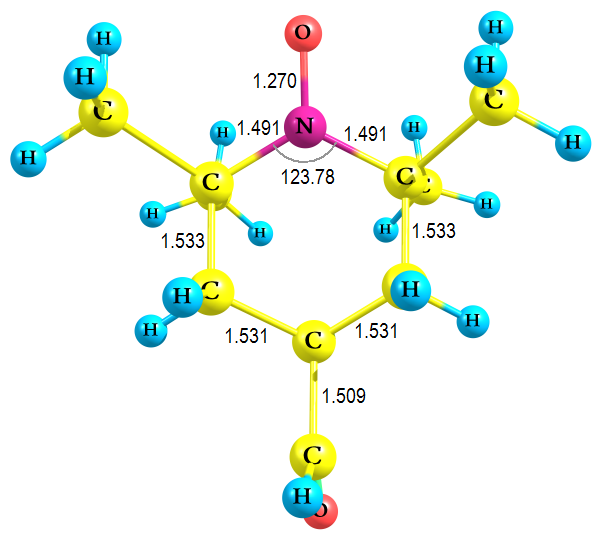 N 1.32243900 0.00004500 -0.21406700  C 0.64994800 -1.32960600 -0.01381500  O 2.59814400 0.00010300 -0.11723000  C 0.64982800 1.32963700 -0.01382100  C -0.82924900 -1.24621700 -0.43914600  C 0.78986700 -1.75815700 1.46143500  C 1.37327100 -2.34836800 -0.90817600  C 1.37305800 2.34846000 -0.90818700  C 0.78970800 1.75820600 1.46142700  C -0.82936200 1.24611200 -0.43915100  C -1.55697400 -0.00008600 0.08859500  H -1.32957500 -2.15782900 -0.09893500  H -0.88316000 -1.24389200 -1.53439500  H 0.44055100 -2.78715000 1.58200500  H 0.21138500 -1.12640400 2.13718600  H 1.83871600 -1.71173200 1.75890600  H 0.88042300 -3.32063300 -0.82450700  H 2.41525900 -2.45338500 -0.60900100  H 1.34701200 -2.03412200 -1.95424500  H 0.88012600 3.32068300 -0.82451900  H 1.34682400 2.03420900 -1.95425500  H 2.41503900 2.45356900 -0.60901500  H 0.44029400 2.78716700 1.58199400  H 1.83856200 1.71188300 1.75889600  H 0.21128800 1.12640000 2.13718100  H -1.32977100 2.15767800 -0.09894200  H -0.88327300 1.24377700 -1.53440100  H -1.59541700 -0.00008300 1.18040800  C -2.98970200 -0.00014000 -0.39866000  H -3.10543700 -0.00012300 -1.50690300  O -3.96292800 -0.00002600 0.31322900  Zero-point correction= 0.269533 (Hartree/Particle)  Thermal correction to Energy= 0.283797  Thermal correction to Enthalpy= 0.284741  Thermal correction to Gibbs Free Energy= 0.229215  Sum of electronic and zero-point Energies= -596.935545  Sum of electronic and thermal Energies= -596.921281  Sum of electronic and thermal Enthalpies= -596.920337  Sum of electronic and thermal Free Energies= -596.975863  **TEMPO-NO_2_** [optimized at the M06-2X/6-311++G(d,p) level]  0 2  N -1.50874000 -0.00000100 -0.28250000  C -0.86642600 1.31728700 -0.00131300  O -2.77746000 -0.00000300 -0.30299300  C -0.86642300 -1.31728800 -0.00131500  C 0.63352500 1.24258400 -0.30999600  C -1.12947000 1.71512200 1.45807600  C -1.51224800 2.34534800 -0.93051500  C -1.51224600 -2.345350 00 -0.93051600  C -1.12946200 -1.71512300 1.45807400  C 0.63352700 -1.24258200 -0.31000000  C 1.26930100 0.00000100 0.29205200  H 1.11172600 2.14765200 0.07464500  H 0.78878100 1.21164500 -1.39172700  H -0.82883200 2.75392200 1.61265300  H -0.58231000 1.09644100 2.17064900  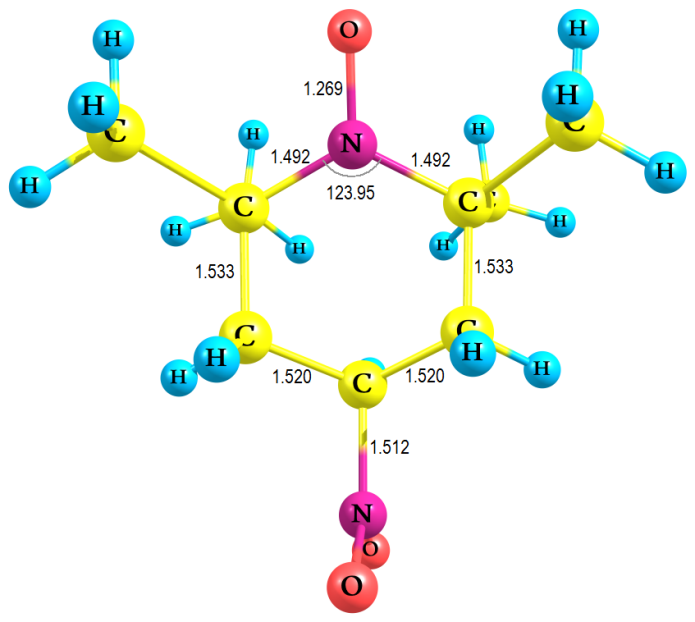 H -2.19672600 1.62546500 1.66706400  H -1.00315100 3.30483900 -0.81518500  H -2.56672500 2.46804000 -0.68869400  H -1.42920100 2.02370800 -1.97028600  H -1.00314700 -3.30484000 -0.81518700  H -1.42920100 -2.02371000 -1.97028700  H -2.56672200 -2.46804300 -0.68869300  H -0.82882500 -2.75392300 1.61265000  H -2.19671800 -1.62546500 1.66706700  H -0.58229800 -1.09644300 2.17064500  H 1.11173000 -2.14765000 0.07463700  H 0.78878200 -1.21163800 -1.39173100  H 1.24256600 -0.00000200 1.38013200  N 2.74574700 0.00000100 -0.03164700  O 3.51964900 -0.00000500 0.89897200  O 3.05665600 0.00000800 -1.20180400  Zero-point correction= 0.267420 (Hartree/Particle)  Thermal correction to Energy= 0.281849  Thermal correction to Enthalpy= 0.282793  Thermal correction to Gibbs Free Energy= 0.226107  Sum of electronic and zero-point Energies= -687.857134  Sum of electronic and thermal Energies= -687.842706  Sum of electronic and thermal Enthalpies= -687.841761  Sum of electronic and thermal Free Energies= -687.898447  **TEMPO-H^+^**  [H-atom bonded to the nitrogen atom of the NO moiety at the B3LYP/6-311++G(d,p) level]  1 2  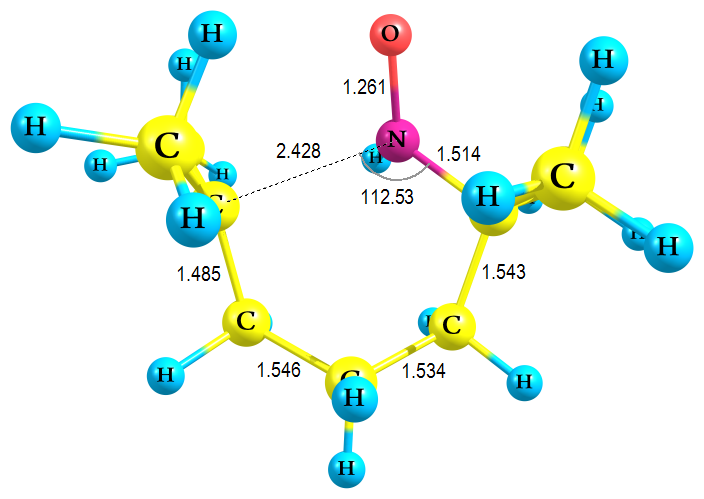 N -0.39053000 -0.91604200 -0.45766100  C -1.50969300 0.00908500 -0.02823900  O -0.24034600 -2.05062300 0.07073000  C 1.80786600 0.01520100 -0.01478900  C -1.10948600 1.44131700 -0.43845900  C -1.75708600 -0.15434800 1.47054400  C -2.74924800 -0.43654500 -0.83039300  C 2.47755600 -0.86011300 -1.00691600  C 1.91526100 -0.37565200 1.40750300  C 1.46996400 1.41024300 -0.39411200  C 0.17013200 2.02281300 0.17548200  H -1.94875000 2.08695900 -0.16680700  H -1.04248400 1.49744800 -1.53266100  H -2.58787600 0.49182600 1.75961100  H -0.89316800 0.11903200 2.07633300  H -2.02576300 -1.18246900 1.71134700  H -3.58934600 0.21814200 -0.58810700  H -3.02536500 -1.46154300 -0.57703000  H -2.57899800 -0.37357400 -1.90900200  H 3.54908700 -0.60276800 -0.96898200  H 2.15610300 -0.67657100 -2.03311800  H 2.39958800 -1.91801900 -0.75216400  H 2.98701900 -0.47677900 1.63534600  H 1.48999300 -1.37094500 1.57148600  H 1.48019500 0.34164700 2.09922700  H 2.31827900 1.99753600 0.00308000  H 1.49985800 1.52902600 -1.48025100  H 0.15316100 1.95255500 1.26503800  H 0.20297500 3.08993600 -0.05426900  H -0.17962600 -0.87615900 -1.46502600  Zero-point correction= 0.269235 (Hartree/Particle)  Thermal correction to Energy= 0.282761  Thermal correction to Enthalpy= 0.283705  Thermal correction to Gibbs Free Energy= 0.230488  Sum of electronic and zero-point Energies= -483.901831  Sum of electronic and thermal Energies= -483.888305  Sum of electronic and thermal Enthalpies= -483.887360  Sum of electronic and thermal Free Energies= -483.940578  **TEMPO-CH_3_-H^+^**  [H-atom bonded to the nitrogen atom of the NO moiety at the B3LYP/6-311++G(d,p) level]  1 2  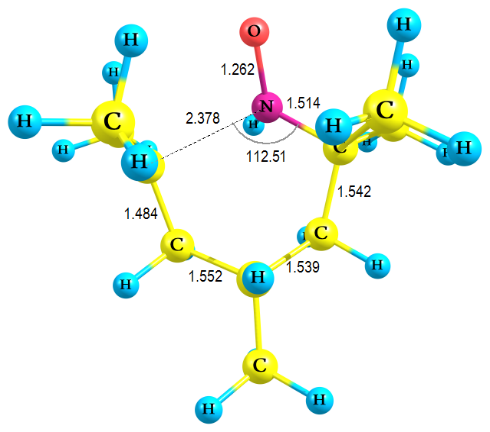 N -0.61666200 -1.11778300 -0.46929400  C -1.54206700 -0.00584500 -0.02201400  O -0.70532200 -2.27620500 0.02313800  C 1.66732300 -0.64173700 -0.00930100  C -0.88046700 1.33474600 -0.40004900  C -1.83128300 -0.15235700 1.47126200  C -2.83727300 -0.19388200 -0.83910300  C 2.17134800 -1.60772200 -1.01845500  C 1.70749700 -1.07452900 1.40670000  C 1.62012900 0.79997000 -0.35776100  C 0.48232000 1.67982300 0.22588400  H -1.59404800 2.11447400 -0.11993900  H -0.79560900 1.40052400 -1.49330900  H -2.52837900 0.63270800 1.76970200  H -0.93815900 -0.05951800 2.08872300  H -2.29207600 -1.11604900 1.68635300  H -3.54130800 0.60175300 -0.58563200  H -3.30277300 -1.15392500 -0.60933800  H -2.64971700 -0.14132800 -1.91541300  H 3.27117300 -1.55986600 -0.96740200  H 1.90101000 -1.34437700 -2.04209400  H 1.88960900 -2.63647700 -0.78985300  H 2.75173200 -1.34122000 1.62888700  H 1.13295700 -1.99334300 1.55453200  H 1.39163700 -0.31055600 2.11287800  H 2.57353100 1.18670800 0.04771000  H 1.67547700 0.93532000 -1.44179600  H 0.43507900 1.52665100 1.30754900  H -0.39791300 -1.09384600 -1.47553300  C 0.82025000 3.15747300 -0.02361600  H 0.05198500 3.80469800 0.40407100  H 1.77549100 3.42894800 0.43230600  H 0.88286000 3.37719800 -1.09373200  Zero-point correction= 0.297089 (Hartree/Particle)  Thermal correction to Energy= 0.312051  Thermal correction to Enthalpy= 0.312996  Thermal correction to Gibbs Free Energy= 0.256801  Sum of electronic and zero-point Energies= -523.199575  Sum of electronic and thermal Energies= -523.184613  Sum of electronic and thermal Enthalpies= -523.183669  Sum of electronic and thermal Free Energies= -523.239863  **TEMPO-NH_2_-H^+^**  [H-atom bonded to the nitrogen atom of the NO moiety at the B3LYP/6-311++G(d,p) level]  1 2  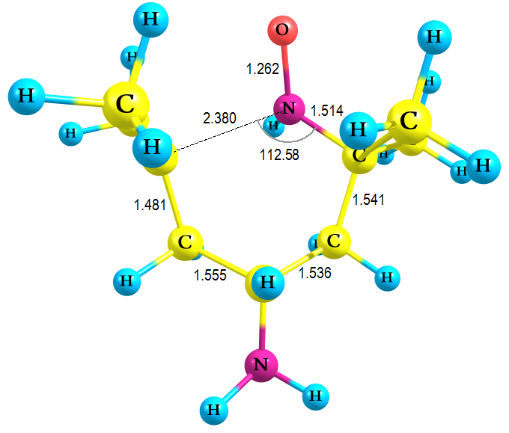  N -0.54798200 -1.13640300 -0.47130400  C -1.53642900 -0.08201700 -0.01947200  O -0.57242600 -2.30051400 0.01510900  C 1.70771500 -0.53293400 -0.00837100  C -0.95350900 1.29424000 -0.39276400  C -1.82203100 -0.25491900 1.47221100  C -2.81709600 -0.33819100 -0.84141000  C 2.26619400 -1.45837300 -1.02632700  C 1.77086000 -0.97669400 1.40367900  C 1.57480700 0.90262700 -0.34924000  C 0.38391500 1.71274100 0.23703000  H -1.70904300 2.03182000 -0.10571200  H -0.86098400 1.38013200 -1.48224400  H -2.55989800 0.49051500 1.77430700  H -0.93725800 -0.12161700 2.09416600  H -2.23368400 -1.24271500 1.67736100  H -3.56712000 0.41283200 -0.58389000  H -3.22567600 -1.32543300 -0.61903300  H -2.63049700 -0.26686200 -1.91663200  H 3.36157400 -1.34850700 -0.97378400  H 1.98275900 -1.19949800 -2.04745000  H 2.04334500 -2.50372300 -0.80809800  H 2.83269000 -1.15722400 1.63065900  H 1.27343800 -1.94113000 1.53795900  H 1.39109800 -0.24843500 2.11603400  H 2.49744000 1.35182100 0.06288100  H 1.61344200 1.06547900 -1.42854300  H 0.34565800 1.55350200 1.32253400  H -0.32940300 -1.09415400 -1.47694400  N 0.62933700 3.10483000 -0.12629700  H -0.16441600 3.69809500 0.08984900  H 1.43991700 3.49134700 0.34689300  Zero-point correction= 0.286206 (Hartree/Particle)  Thermal correction to Energy= 0.301061  Thermal correction to Enthalpy= 0.302005  Thermal correction to Gibbs Free Energy= 0.246052  Sum of electronic and zero-point Energies= -539.251135  Sum of electronic and thermal Energies= -539.236280  Sum of electronic and thermal Enthalpies= -539.235336  Sum of electronic and thermal Free Energies= -539.291288  **TEMPO-CHO-H^+^**  [H-atom bonded to the nitrogen atom of the NO moiety at the B3LYP/6-311++G(d,p) level]  1 2  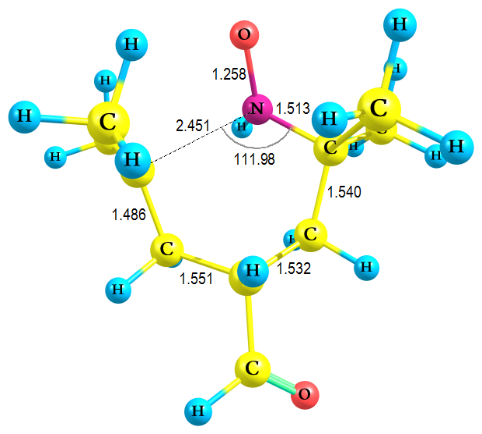  N -1.35049300 0.66824300 -0.47799400  C -0.22176500 1.56157000 -0.01076600  O -2.50081800 0.74329700 0.02449400  C -0.89137000 -1.69815300 -0.03389600  C 1.10335300 0.86316400 -0.37000400  C -0.38706000 1.85010200 1.48032800  C -0.35533900 2.86227300 -0.83000200  C -1.82481400 -2.15169300 -1.09266700  C -1.38271600 -1.73296000 1.35972000  C 0.56460700 -1.66099700 -0.32737200  C 1.38329100 -0.49362400 0.28328300  H 1.91243700 1.54470800 -0.09939900  H 1.17949300 0.75711300 -1.45929200  H 0.42848800 2.49975600 1.80281500  H -0.36412800 0.94927300 2.09449400  H -1.32868900 2.36220600 1.67597100  H 0.45399100 3.54261500 -0.55613100  H -1.30717000 3.35373500 -0.62185200  H -0.28158600 2.67462200 -1.90491800  H -1.80961400 -3.25427100 -1.06572400  H -1.51177200 -1.86756200 -2.09841900  H -2.85515300 -1.84887400 -0.89912800  H -1.74780800 -2.75280800 1.55275500  H -2.25957300 -1.08717700 1.47844700  H -0.63017100 -1.48588700 2.10528200  H 0.94911600 -2.60656400 0.10019800  H 0.74078200 -1.71591400 -1.40477700  H 1.18929700 -0.45046600 1.36269200  H -1.32748400 0.45470000 -1.48556000  C 2.87105600 -0.86446800 0.18443000  H 3.11752200 -1.88635600 0.54558300  O 3.71957000 -0.12727700 -0.22891800  Zero-point correction= 0.277660 (Hartree/Particle)  Thermal correction to Energy= 0.293298  Thermal correction to Enthalpy= 0.294242  Thermal correction to Gibbs Free Energy= 0.235573  Sum of electronic and zero-point Energies= -597.234482  Sum of electronic and thermal Energies= -597.218845  Sum of electronic and thermal Enthalpies= -597.217900  Sum of electronic and thermal Free Energies= -597.276570  **TEMPO-NO_2_-H^+^**  [H-atom bonded to the nitrogen atom of the NO moiety at the B3LYP/6-311++G(d,p) level]  1 2  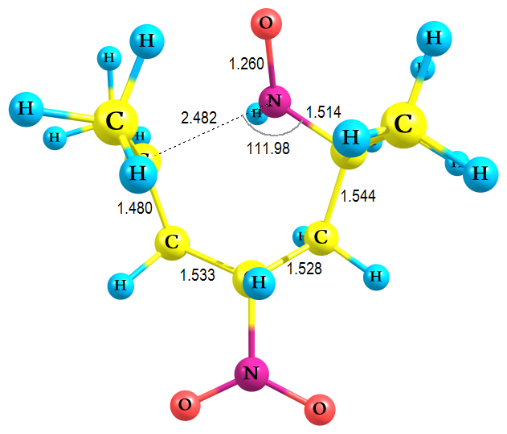 N 1.77386200 -0.21288300 -0.44850000  C 0.99062300 -1.44587700 -0.05008000  O 2.84715300 0.09657700 0.13369800  C 0.50612400 1.87531500 -0.00941300  C -0.48073700 -1.23202800 -0.46767700  C 1.17235700 -1.70023800 1.44598500  C 1.58280700 -2.60845800 -0.87228700  C 1.27786600 2.67345100 -0.98933800  C 0.86820500 2.02592600 1.41468800  C -0.80060600 1.31552600 -0.42188400  C -1.18927200 -0.04153800 0.17646500  H -1.02476600 -2.13902100 -0.20288200  H -0.54926300 -1.14297900 -1.55840800  H 0.59948100 -2.58673600 1.72342500  H 0.83400200 -0.87015300 2.06748800  H 2.21997000 -1.88269700 1.68262800  H 1.03848400 -3.52728100 -0.64332700  H 2.63427600 -2.75759100 -0.62171100  H 1.49822200 -2.43022900 -1.94800000  H 0.85683300 3.69275800 -0.95761000  H 1.16601600 2.32781000 -2.01767700  H 2.33000800 2.76385000 -0.71389100  H 0.89065400 3.10190100 1.64043800  H 1.89528100 1.67992700 1.58281100  H 0.18916900 1.53635800 2.10902600  H -1.54748000 2.05395500 -0.07329600  H -0.89290700 1.28898800 -1.50996800  H -1.06438300 -0.05187500 1.26069700  H 1.74854700 -0.00235900 -1.45646600  N -2.73220800 -0.20193100 0.03294500  O -3.16972600 -1.33152900 0.09245900  O -3.38716900 0.81952700 -0.07780100  Zero-point correction= 0.271177 (Hartree/Particle)  Thermal correction to Energy= 0.287415  Thermal correction to Enthalpy= 0.288359  Thermal correction to Gibbs Free Energy= 0.226811  Sum of electronic and zero-point Energies= -688.446809  Sum of electronic and thermal Energies= -688.430571  Sum of electronic and thermal Enthalpies= -688.429627  Sum of electronic and thermal Free Energies= -688.491174  **TEMPO-H^+^**  [H-atom bonded to the oxygen atom of the NO moiety at the B3LYP/6-311++G(d,p) level]  1 2  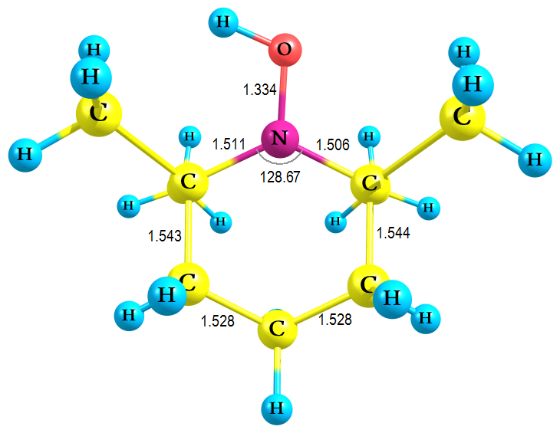 N 0.00149217 -0.68198905 -0.14464466  C -1.35428271 -0.02921464 -0.01223934  O 0.05053875 -2.01388824 -0.08157878  C 1.36445740 -0.05477763 -0.01554261  C -1.23614023 1.41742138 -0.53472599  C -1.76276447 -0.07653609 1.47920026  C -2.36027748 -0.82227650 -0.86628510  C 2.35139500 -0.85753438 -0.87955535  C 1.77785032 -0.12729576 1.47397260  C 1.26492509 1.39964222 -0.52274809  C 0.01761720 2.14615712 -0.05245189  H -2.14231854 1.94211933 -0.22293718  H -1.24333001 1.39854657 -1.62932688  H -2.78945361 0.28684821 1.55677817  H -1.13120199 0.55614445 2.10053550  H -1.73488665 -1.09340597 1.87675419  H -3.29705280 -0.26312756 -0.88551404  H -2.61716638 -1.80361521 -0.44825885  H -2.02009441 -0.93975021 -1.89716043  H 3.30220159 -0.32148095 -0.87987429  H 2.00667919 -0.93347549 -1.91264647  H 2.52559015 -1.85855904 -0.48718290  H 2.80976369 0.22105896 1.54794789  H 1.73835568 -1.15285216 1.84356007  H 1.16113241 0.50633722 2.11063073  H 2.17374311 1.91021039 -0.19667475  H 1.28651962 1.39056529 -1.61721819  H 0.01224462 2.26571474 1.03473282  H 0.02485109 3.15667018 -0.46653615  H -0.85701270 -2.35643379 -0.18021335  Zero-point correction= 0.273030 (Hartree/Particle)  Thermal correction to Energy= 0.285460  Thermal correction to Enthalpy= 0.286404  Thermal correction to Gibbs Free Energy= 0.236029  Sum of electronic and zero-point Energies= -483.933967  Sum of electronic and thermal Energies= -483.921537  Sum of electronic and thermal Enthalpies= -483.920593  Sum of electronic and thermal Free Energies= -483.970968  **TEMPO-CH_3_-H^+^**  [H-atom bonded to the oxygen atom of the NO moiety at the B3LYP/6-311++G(d,p) level]  1 2  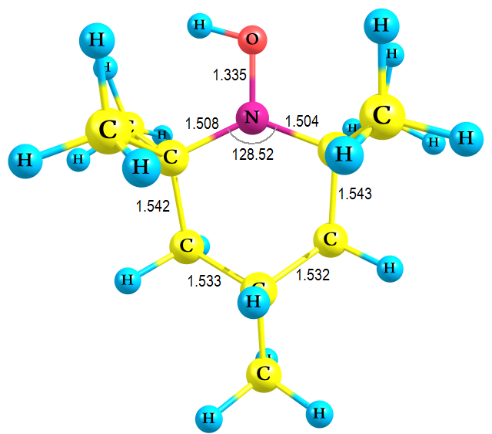  N 0.98600900 -0.03876900 -0.19009400  C 0.31881500 -1.37293000 -0.00129300  O 2.31484500 -0.14132700 -0.25883300  C 0.40300000 1.33874800 0.00362200  C -1.17490300 -1.20921800 -0.35037000  C 0.53342800 -1.80408400 1.46986500  C 0.98243200 -2.38531400 -0.95077400  C 1.13870900 2.31292100 -0.93538000  C 0.62634200 1.74811700 1.47915500  C -1.09341700 1.27646700 -0.36193700  C -1.83906800 0.05897300 0.19595000  H -1.68327000 -2.10126900 0.02401600  H -1.28504900 -1.22265300 -1.44041200  H 0.14539000 -2.81877000 1.57696500  H 0.00371500 -1.16373100 2.17452700  H 1.59396000 -1.81728900 1.72485900  H 0.41019800 -3.31321300 -0.89739700  H 2.01168800 -2.60350600 -0.66882900  H 0.96139100 -2.03501300 -1.98455300  H 0.61817400 3.27113200 -0.89883900  H 1.13136300 1.96753600 -1.97113800  H 2.16891500 2.53016100 -0.62651700  H 0.30743500 2.78637900 1.59051500  H 1.67919400 1.68746300 1.76344600  H 0.04500300 1.13875100 2.16901600  H -1.54419800 2.20495700 -0.00166400  H -1.19257600 1.28241100 -1.45313400  H -1.77028900 0.06252700 1.28962800  C -3.32573100 0.10843300 -0.17641700  H -3.85843500 -0.75502800 0.22844300  H -3.80050900 1.00840400 0.22139100  H -3.46247500 0.10792600 -1.26205100  H 2.68190800 0.75213100 -0.39147700  Zero-point correction= 0.300685 (Hartree/Particle)  Thermal correction to Energy= 0.314641  Thermal correction to Enthalpy= 0.315585  Thermal correction to Gibbs Free Energy= 0.261997  Sum of electronic and zero-point Energies= -523.232670  Sum of electronic and thermal Energies= -523.218714  Sum of electronic and thermal Enthalpies= -523.217770  Sum of electronic and thermal Free Energies= -523.271358  **TEMPO-NH_2_-H^+^**  [H-atom bonded to the oxygen atom of the NO moiety at the B3LYP/6-311++G(d,p) level]  1 2  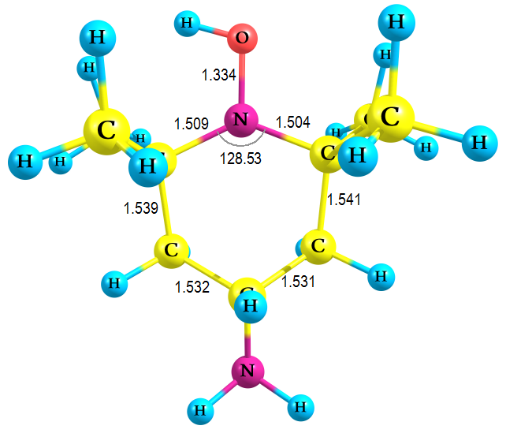  N 0.97306639 -0.06130151 -0.18533655  C 0.27280743 -1.37943827 -0.00065022  O 2.29880975 -0.19587350 -0.24662750  C 0.42191758 1.33032616 0.00341449  C -1.21395027 -1.17855999 -0.35153094  C 0.48266038 -1.82335301 1.46766070  C 0.90884287 -2.40298099 -0.95773685  C 1.17702693 2.28215595 -0.94338943  C 0.66076116 1.74179604 1.47637543  C -1.07284296 1.30094667 -0.36378240  C -1.84409243 0.10304824 0.19908696  H -1.74663199 -2.05746444 0.02182481  H -1.33815665 -1.17249124 -1.43846165  H 0.06071450 -2.82451895 1.57389827  H -0.01919002 -1.16792161 2.17888806  H 1.54404620 -1.87446861 1.71412503  H 0.31616427 -3.31798319 -0.90601008  H 1.93394507 -2.64485116 -0.68005962  H 0.89087231 -2.04751234 -1.98966014  H 0.68056801 3.25309402 -0.90967315  H 1.15668276 1.93163852 -1.97708757  H 2.21342616 2.47441047 -0.63884356  H 0.35544695 2.78430855 1.58611774  H 1.71506242 1.67011002 1.75281116  H 0.07738831 1.14203156 2.17298229  H -1.50568127 2.23948026 -0.00610658  H -1.18831977 1.29269457 -1.45164148  H -1.77232592 0.10425595 1.29757227  N -3.22296541 0.18316309 -0.27441896  H -3.77026851 -0.61715282 0.02620368  H -3.69204213 1.01499490 0.06988766  H 2.68833024 0.68765764 -0.38214500  Zero-point correction= 0.289843 (Hartree/Particle)  Thermal correction to Energy= 0.303687  Thermal correction to Enthalpy= 0.304632  Thermal correction to Gibbs Free Energy= 0.251264  Sum of electronic and zero-point Energies= -539.283833  Sum of electronic and thermal Energies= -539.269989  Sum of electronic and thermal Enthalpies= -539.269045  Sum of electronic and thermal Free Energies= -539.322412  **TEMPO-CHO-H^+^**  [H-atom bonded to the oxygen atom of the NO moiety at the B3LYP/6-311++G(d,p) level]  1 2  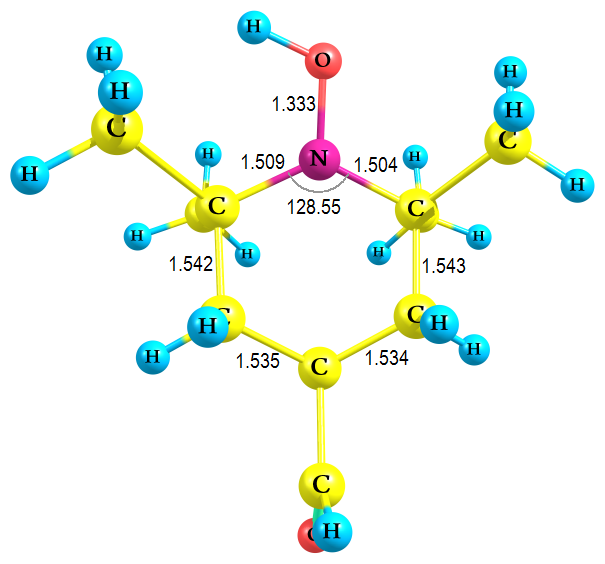 N 1.25113290 -0.00300872 -0.16017503  C 0.59852850 -1.35384082 -0.00057859  O 2.58357633 0.04161001 -0.15427601  C 0.63556330 1.36032053 0.00016354  C -0.86899997 -1.22995617 -0.45848397  C 0.70923408 -1.76929720 1.48573876  C 1.34399008 -2.36322562 -0.89355165  C 1.39796457 2.34689656 -0.90099731  C 0.77673277 1.77432899 1.48472204  C -0.84065151 1.27003503 -0.44047050  C -1.58037355 0.02524188 0.06693574  H -1.38173951 -2.13475863 -0.12285743  H -0.90170893 -1.23255534 -1.55349992  H 0.33790149 -2.79206453 1.57642527  H 0.11447577 -1.13383641 2.13956609  H 1.74428590 -1.75526257 1.83377565  H 0.77886680 -3.29663381 -0.88970758  H 2.34099565 -2.62696096 -0.51983001  H 1.41805889 -2.02061022 -1.92767207  H 0.86595963 3.29959159 -0.87447896  H 1.42494038 2.00306887 -1.93684057  H 2.41652370 2.51727641 -0.55504656  H 0.43104130 2.80584557 1.57504537  H 1.81902358 1.73642681 1.80386483  H 0.17520052 1.15742840 2.15153707  H -1.32946507 2.18076815 -0.08644361  H -0.88438050 1.29297963 -1.53479129  H -1.63809869 0.01494302 1.15764015  C -3.02623239 0.03729023 -0.42850817  H -3.15228289 0.09891164 -1.53160837  O -3.97518499 -0.01832744 0.30344898  H 2.92080566 -0.86651760 -0.26705596  Zero-point correction= 0.281568 (Hartree/Particle)  Thermal correction to Energy= 0.296272  Thermal correction to Enthalpy= 0.297217  Thermal correction to Gibbs Free Energy= 0.240494  Sum of electronic and zero-point Energies= -597.265126  Sum of electronic and thermal Energies= -597.250422  Sum of electronic and thermal Enthalpies= -597.249478  Sum of electronic and thermal Free Energies= -597.306200  **TEMPO-NO_2_-H^+^**  [H-atom bonded to the oxygen atom of the NO moiety at the B3LYP/6-311++G(d,p) level]  1 2  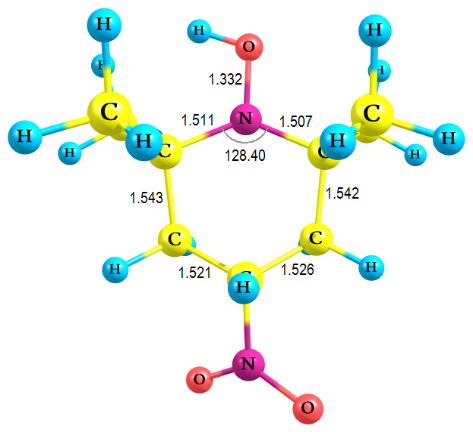  N -1.46079500 0.03017800 -0.21587900  C -0.80245000 1.37687400 -0.06399600  O -2.78286700 0.12721700 -0.34257800  C -0.88403100 -1.33526000 0.07869800  C 0.70147400 1.20463600 -0.35767500  C -1.06687400 1.87869400 1.37576600  C -1.42530600 2.34270700 -1.08653200  C -1.58017800 -2.36344500 -0.83189800  C -1.16002300 -1. 66364300 1.56562100  C 0.62433300 -1.29832300 -0.24378200  C 1.30215300 -0.04332400 0.28355200  H 1.21468300 2.09684300 0.00191300  H 0.85459600 1.15260600 -1.43999600  H -0.67982400 2.89677400 1.44766400  H -0.56800700 1.27501400 2.13378500  H -2.13611800 1.90511100 1.58901900  H -0.85551100 3.27308100 -1.05469100  H -2.46389500 2.57162800 -0.85224700  H -1.36601000 1.94362200 -2.10084200  H -1.06006400 -3.31646000 -0.72305800  H -1.53533500 -2.07642900 -1.88432700  H -2.61978300 -2.56548600 -0.54653400  H -0.83530100 -2.69008400 1.74737500  H -2.22416900 -1.60091200 1.80243300  H -0.61697900 -1.01094200 2.24754900  H 1.07423500 -2.19588500 0.18226200  H 0.77107600 -1.35585700 -1.32477000  H 1.27534300 0.01844800 1.37283100  N 2.80696900 -0.06201200 -0.02403600  O 3.25543600 -1.02396000 -0.61995600  O 3.43267100 0.90665600 0.36392400  H -3.14865400 -0.77104200 -0.44861100  Zero-point correction= 0.275395 (Hartree/Particle)  Thermal correction to Energy= 0.290507  Thermal correction to Enthalpy= 0.291452  Thermal correction to Gibbs Free Energy= 0.233614  Sum of electronic and zero-point Energies= -688.479365  Sum of electronic and thermal Energies= -688.464252  Sum of electronic and thermal Enthalpies= -688.463308  Sum of electronic and thermal Free Energies= -688.521145  **TEMPO-H^+^**  [H-atom bonded to the nitrogen atom of the NO moiety at the M06-2X/6-311++G(d,p) level]  1 2  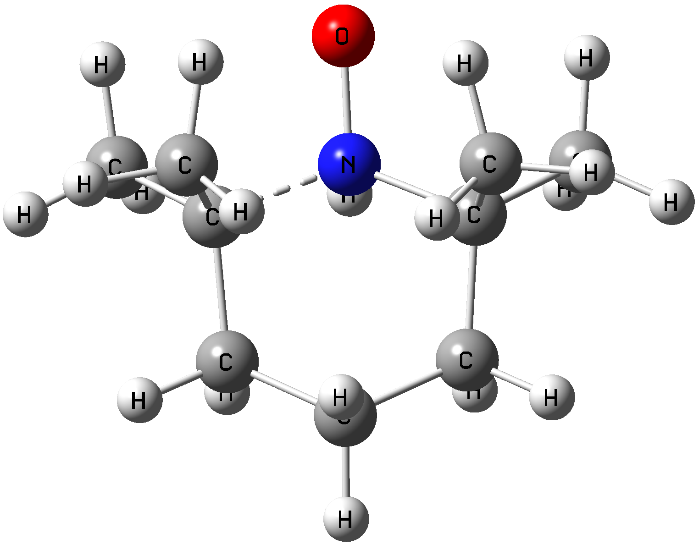 N -0.02103000 -0.71208900 -0.44127900  C -1.36272100 -0.05279400 -0.01109900  O 0.03652100 -2.00889700 -0.08405500  C 1.37211100 -0.03778900 -0.00642500  C -1.26928800 1.40102500 -0.47643100  C -1.59077400 -0.20374600 1.48771300  C -2.42455200 -0.82549300 -0.79267300  C 2.43270600 -0.78661600 -0.80643000  C 1.59747200 -0.20319500 1.48881700  C 1.24237100 1.41471900 -0.46325800  C -0.01998400 2.12628100 0.01973300  H -2.17601100 1.90151100 -0.12717400  H -1.30137700 1.43375200 -1.57259000  H -2.65743100 -0.05937900 1.66730400  H -1.05855900 0.53590300 2.07993900  H -1.33205400 -1.20379600 1.83703200  H -3.39069600 -0.34314000 -0.63891500  H -2.50029800 -1.85826100 -0.44369500  H -2.22209700 -0.82403100 -1.86767300  H 3.37641900 -0.25093900 -0.68871900  H 2.20155800 -0.80879400 -1.87499800  H 2.57124100 -1.80711700 -0.44852100  H 2.64438700 0.04625000 1.67439500  H 1.44245100 -1.23204700 1.81564500  H 0.98924900 0.46281900 2.09406500  H 2.13876600 1.92281000 -0.09550600  H 1.29054500 1.45213700 -1.55810500  H -0.02442100 2.21479000 1.10834100  H -0.01997000 3.14575900 -0.36802700  H 0.01928100 -0.66678400 -1.47109800  Zero-point correction= 0.275732 (Hartree/Particle)  Thermal correction to Energy= 0.287842  Thermal correction to Enthalpy= 0.288786  Thermal correction to Gibbs Free Energy= 0.239193  Sum of electronic and zero-point Energies= -483.663980  Sum of electronic and thermal Energies= -483.651870  Sum of electronic and thermal Enthalpies= -483.650926  Sum of electronic and thermal Free Energies= -483.700519  **TEMPO-CH_3_-H^+^**  [H-atom bonded to the nitrogen atom of the NO moiety at the M06-2X/6-311++G(d,p) level]  1 2  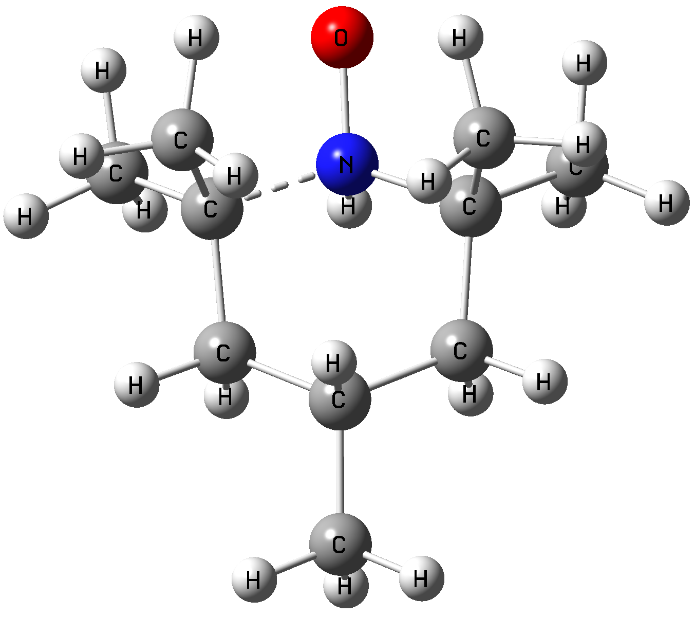 N -0.98563700 0.05143000 -0.49027800  C -0.32407800 1.36986100 0.00231700  O -2.31179700 0.03973300 -0.26016000  C -0.40152200 -1.35826700 0.00758900  C 1.16267000 1.22248300 -0.31980800  C -0.61478900 1.61142300 1.47844900  C -0.97808300 2.45576700 -0.85202900  C -1.10183100 -2.39762400 -0.86223900  C -0.72070500 -1.57668600 1.47913100  C 1.09095900 -1.27511600 -0.30624000  C 1.81737300 -0.04296000 0.23810700  H 1.65904700 2.11428300 0.07428300  H 1.30553400 1.24805700 -1.40880200  H -0.44544100 2.67236100 1.66995500  H 0.03903700 1.05308400 2.14283900  H -1.65473200 1.39586100 1.72565000  H -0.47787500 3.40437700 -0.65304100  H -2.03641700 2.57050800 -0.60519000  H -0.87958100 2.24841700 -1.92153500  H -0.61172700 -3.35782200 -0.69190600  H -1.01110200 -2.16945400 -1.92779700  H -2.15673500 -2.50139700 -0.60650100  H -0.52171900 -2.63023700 1.68671200  H -1.77194400 -1.39104100 1.70240600  H -0.10031400 -0.98778000 2.14831800  H 1.53284900 -2.18815000 0.10641300  H 1.23593600 -1.32569300 -1.39352000  H 1.74696300 -0.03669300 1.33005900  H -0.84143000 0.00791500 -1.51067100  C 3.29693500 -0.09139900 -0.13941600  H 3.82678700 0.77810200 0.25294500  H 3.77419500 -0.98638600 0.26331100  H 3.42093000 -0.10107400 -1.22587100  Zero-point correction= 0.303604 (Hartree/Particle)  Thermal correction to Energy= 0.317242  Thermal correction to Enthalpy= 0.318187  Thermal correction to Gibbs Free Energy= 0.265389  Sum of electronic and zero-point Energies= -522.943056  Sum of electronic and thermal Energies= -522.929417  Sum of electronic and thermal Enthalpies= -522.928473  Sum of electronic and thermal Free Energies= -522.981270  **TEMPO-NH_2_-H^+^**  [H-atom bonded to the nitrogen atom of the NO moiety at the M06-2X/6-311++G(d,p) level]  1 2  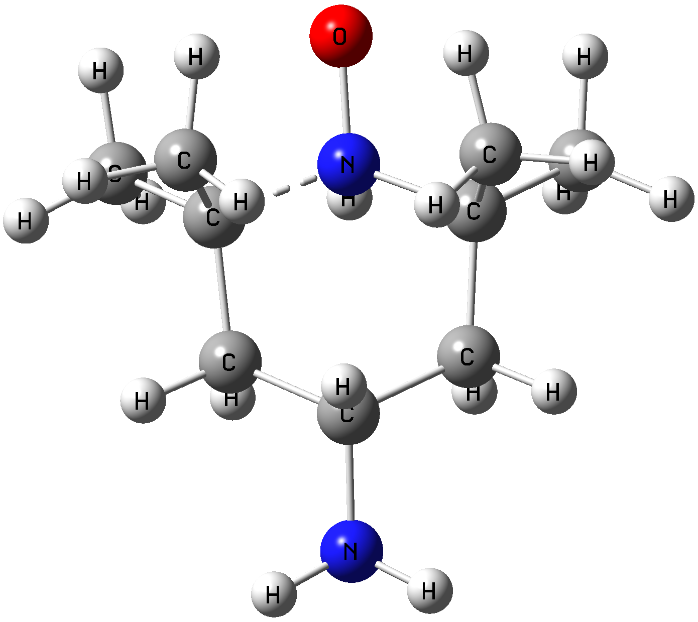 N -0.97282800 0.07525200 -0.48889100  C -0.27858300 1.37590800 0.00326500  O -2.29879400 0.09494200 -0.25744800  C -0.42207600 -1.34898500 0.00822100  C 1.20167400 1.19138100 -0.32208300  C -0.56733600 1.62846100 1.47852900  C -0.90226000 2.47774100 -0.85377500  C -1.14340400 -2.37053100 -0.86553100  C -0.75203600 -1.56331000 1.47848900  C 1.06930400 -1.29988900 -0.30822000  C 1.82404000 -0.08716200 0.23934900  H 1.72276000 2.07114500 0.06830900  H 1.35739200 1.19351900 -1.40794300  H -0.36207700 2.68312700 1.66964600  H 0.06252800 1.04902000 2.14782800  H -1.61574300 1.45009600 1.71967200  H -0.37969900 3.41392200 -0.65366600  H -1.95807400 2.61823300 -0.60952200  H -0.80510300 2.26735700 -1.92267000  H -0.67725800 -3.34240800 -0.69431200  H -1.04224900 -2.14378100 -1.93031000  H -2.20130200 -2.44866400 -0.61332100  H -0.56889000 -2.61973400 1.68620900  H -1.80230300 -1.36397900 1.69489400  H -0.12825200 -0.98396300 2.15275300  H 1.49291700 -2.22367300 0.10067900  H 1.22851300 -1.33514600 -1.39240300  H 1.75467000 -0.07874100 1.33546600  H -0.82995900 0.02696600 -1.50947300  N 3.19581100 -0.16761000 -0.24025400  H 3.74247300 0.63082300 0.06545600  H 3.66319200 -0.99883700 0.10684200  Zero-point correction= 0.292861 (Hartree/Particle)  Thermal correction to Energy= 0.306341  Thermal correction to Enthalpy= 0.307286  Thermal correction to Gibbs Free Energy= 0.254800  Sum of electronic and zero-point Energies= -538.994084  Sum of electronic and thermal Energies= -538.980604  Sum of electronic and thermal Enthalpies= -538.979660  Sum of electronic and thermal Free Energies= -539.032146  **TEMPO-CHO-H^+^**  [H-atom bonded to the nitrogen atom of the NO moiety at the M06-2X/6-311++G(d,p) level]  1 2  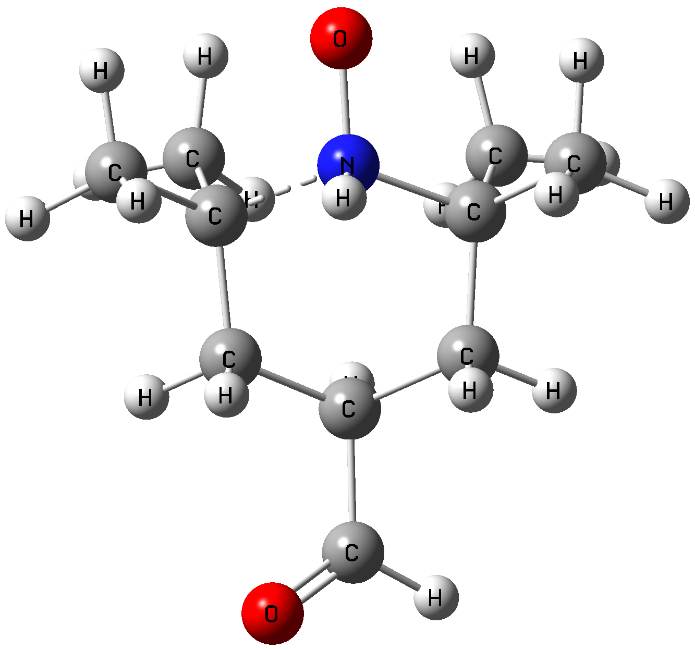 N -1.16502200 0.16511300 -0.52941400  C -0.31073300 1.40965900 0.04561200  O -2.46489600 0.48385500 -0.39355100  C -0.86432300 -1.27159400 -0.02809400  C 1.14118100 0.98886300 -0.15385800  C -0.69398800 1.68776900 1.49106500  C -0.68057500 2.58254100 -0.85530500  C -1.67503700 -2.17292800 -0.95944400  C -1.31779900 -1.45445600 1.41481600  C 0.63827400 -1.47415500 -0.23227100  C 1.49742700 -0.38030400 0.41323400  H 1.75719400 1.76415300 0.31157600  H 1.39477700 1.00974200 -1.21982600  H -0.26653600 2.66093700 1.74215100  H -0.29375400 0.96332000 2.19486100  H -1.77437500 1.76028200 1.62224400  H 0.00335700 3.40188500 -0.62620400  H -1.70000000 2.92728300 -0.68195600  H -0.55527100 2.34254800 -1.91466300  H -1.42840200 -3.21330900 -0.74375400  H -1.44129900 -1.98828900 -2.01195500  H -2.74781900 -2.03816600 -0.80167200  H -1.40727200 -2.52736200 1.59274700  H -2.29904500 -1.01085900 1.58599400  H -0.61333900 -1.06102600 2.14297400  H 0.88429100 -2.45920500 0.17529700  H 0.85924000 -1.51703200 -1.30662300  H 1.36761000 -0.40620100 1.50154300  H -0.93825500 0.19413100 -1.53572300  C 2.96719500 -0.68763500 0.15669300  H 3.32654800 -1.66081400 0.54223300  O 3.69211800 0.05809900 -0.43370300  Zero-point correction= 0.284958 (Hartree/Particle)  Thermal correction to Energy= 0.299112  Thermal correction to Enthalpy= 0.300056  Thermal correction to Gibbs Free Energy= 0.245305  Sum of electronic and zero-point Energies= -596.960030  Sum of electronic and thermal Energies= -596.945876  Sum of electronic and thermal Enthalpies= -596.944932  Sum of electronic and thermal Free Energies= -596.999682  **TEMPO-NO_2_-H^+^**  [H-atom bonded to the nitrogen atom of the NO moiety at the M06-2X/6-311++G(d,p) level]  1 2  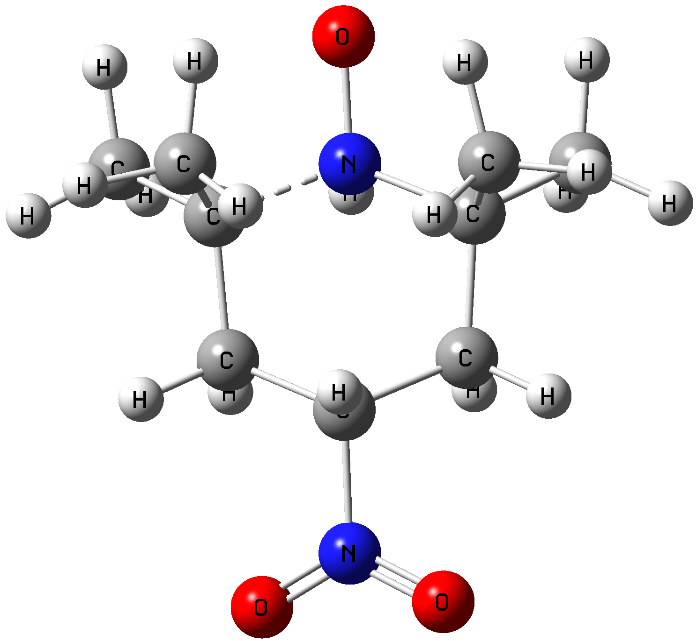  N -1.45120400 0.03614200 -0.50376900  C -0.79677500 1.37462800 -0.06052900  O -2.78122500 0.03399400 -0.29007800  C -0.88021500 -1.35520200 0.06734100  C 0.69816900 1.21766400 -0.33584700  C -1.12083500 1.69138100 1.39490200  C -1.41613900 2.42233400 -0.98500800  C -1.55155400 -2.43216600 -0.77819600  C -1.23958400 -1.51241200 1.53773300  C 0.62076900 -1.29677900 -0.21447100  C 1.28357800 -0.03372100 0.30821000  H 1.20370000 2.10696600 0.04561000  H 0.88287300 1.18194100 -1.41670700  H -0.94835200 2.75934500 1.53739300  H -0.48993000 1.16594900 2.10746600  H -2.16810400 1.49389700 1.62490300  H -0.91731400 3.37740100 -0.81569500  H -2.48016100 2.55173000 -0.77315400  H -1.28803400 2.16414300 -2.04017400  H -1.06763200 -3.38353100 -0.54977300  H -1.42582600 -2.25179800 -1.84925600  H -2.61349100 -2.52448300 -0.55038900  H -1.02069100 -2.54944200 1.80104700  H -2.30247200 -1.34552200 1.71640500  H -0.66134000 -0.87700400 2.20276100  H 1.06183400 -2.18360000 0.24736500  H 0.80672700 -1.38375100 -1.28993000  H 1.25301800 0.03011100 1.39746600  H -1.29789800 -0.05795400 -1.52011200  N 2.77403500 -0.05542200 0.00181300  O 3.38095300 0.94307900 0.29883600  O 3.22886900 -1.05129900 -0.50455200  Zero-point correction= 0.278776 (Hartree/Particle)  Thermal correction to Energy= 0.293462  Thermal correction to Enthalpy= 0.294406  Thermal correction to Gibbs Free Energy= 0.237635  Sum of electronic and zero-point Energies= -688.136515  Sum of electronic and thermal Energies= -688.121830  Sum of electronic and thermal Enthalpies= -688.120885  Sum of electronic and thermal Free Energies= -688.177656  **TEMPO-H^+^**  [H-atom bonded to the oxygen atom of the NO moiety at the M06-2X/6-311++G(d,p) level]  1 2  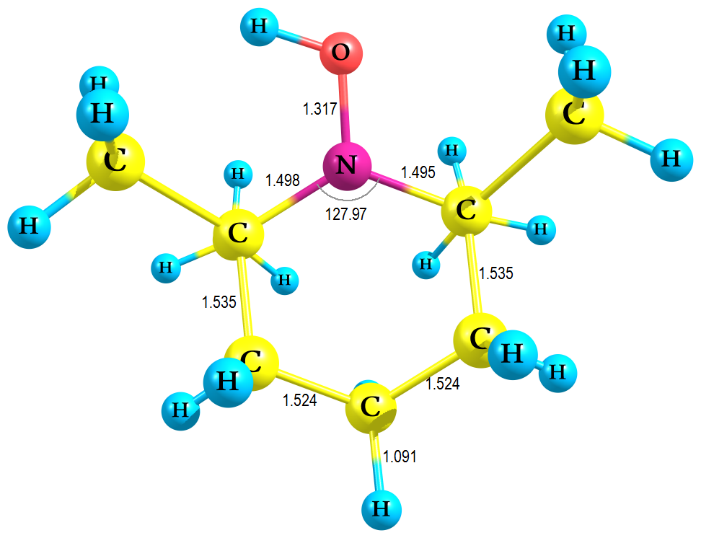 N 0.00115200 -0.67988800 -0.16847600  C -1.33909100 -0.02949500 -0.01277900  O 0.05137200 -1.99460800 -0.10447700  C 1.35005100 -0.05408500 -0.01714400  C -1.22911800 1.40720100 -0.54098700  C -1.71273900 -0.07208800 1.48003700  C -2.36030400 -0.81984600 -0.83928900  C 2.34758300 -0.86391000 -0.84862300  C 1.72519200 -0.11620900 1.47482300  C 1.26102400 1.38775800 -0.53498500  C 0.02078300 2.13548800 -0.06005600  H -2.13720200 1.92743000 -0.22800100  H -1.23392100 1.38063900 -1.63550800  H -2.75285800 0.24650000 1.57062200  H -1.09688300 0.59730500 2.07765900  H -1.63071100 -1.08263100 1.88665600  H -3.27594600 -0.22882200 -0.88402400  H -2.64783200 -1.77427600 -0.38384400  H -2.01560800 -0.98173700 -1.86272600  H 3.29102300 -0.31596900 -0.85269100  H 2.00789600 -0.96677800 -1.88067300  H 2.52489700 -1.85231900 -0.42722400  H 2.76861700 0.19110300 1.56315800  H 1.63583100 -1.13466300 1.85582300  H 1.12081300 0.55357300 2.08503700  H 2.17416500 1.89315700 -0.21354400  H 1.27446400 1.36857900 -1.62946100  H 0.01843800 2.24882800 1.02724100  H 0.02722900 3.14538200 -0.47222100  H -0.85172800 -2.34810100 -0.18711200  Zero-point correction= 0.276462 (Hartree/Particle)  Thermal correction to Energy= 0.288573  Thermal correction to Enthalpy= 0.289517  Thermal correction to Gibbs Free Energy= 0.239860  Sum of electronic and zero-point Energies= -483.702241  Sum of electronic and thermal Energies= -483.690130  Sum of electronic and thermal Enthalpies= -483.689186  Sum of electronic and thermal Free Energies= -483.738843  **TEMPO-CH_3_-H^+^**  [H-atom bonded to the oxygen atom of the NO moiety at the M06-2X/6-311++G(d,p) level]  1 2  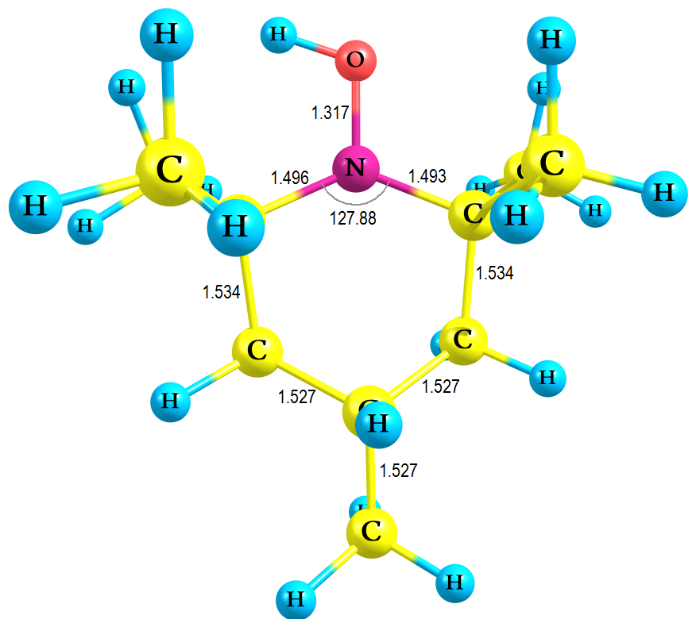  N -0.97735800 0.03808400 -0.21699100  C -0.31642500 1.35934100 -0.00113100  O -2.28839100 0.14106800 -0.29529800  C -0.40072900 -1.32489600 0.00478500  C 1.16899700 1.20565200 -0.35077400  C -0.53393600 1.75187100 1.47199500  C -0.98303700 2.38237500 -0.92449100  C -1.13059400 -2.31410800 -0.91202400  C -0.62935800 -1.69866500 1.48085300  C 1.08890900 -1.27017100 -0.35601600  C 1.82896000 -0.05577700 0.20091400  H 1.67371700 2.10022500 0.02332200  H 1.27845000 1.21179000 -1.44146300  H -0.18556100 2.77910600 1.59190100  H 0.02746800 1.12003600 2.15884500  H -1.59339400 1.71808500 1.73045300  H -0.39966400 3.30261900 -0.86888700  H -2.00531600 2.60313500 -0.62068100  H -0.98004400 2.03813200 -1.96014200  H -0.57496400 -3.25247900 -0.89299600  H -1.16022700 -1.96251200 -1.94539000  H -2.14190500 -2.56325100 -0.57090600  H -0.35679300 -2.74858500 1.60285800  H -1.67743000 -1.58477100 1.76711400  H -0.01490900 -1.10512400 2.15503500  H 1.53585100 -2.19841600 0.01028400  H 1.19046400 -1.27486400 -1.44756500  H 1.76373100 -0.05632200 1.29434200  C 3.30571700 -0.10580700 -0.18587700  H 3.84064100 0.75953000 0.20883900  H 3.78301500 -1.00533500 0.20668700  H 3.42099200 -0.10751200 -1.27325200  H -2.66651300 -0.74750700 -0.41648200  Zero-point correction= 0.304300 (Hartree/Particle)  Thermal correction to Energy= 0.317937  Thermal correction to Enthalpy= 0.318881  Thermal correction to Gibbs Free Energy= 0.266021  Sum of electronic and zero-point Energies= -522.981313  Sum of electronic and thermal Energies= -522.967676  Sum of electronic and thermal Enthalpies= -522.966731  Sum of electronic and thermal Free Energies= -523.019591  **TEMPO-NH_2_-H^+^**  [H-atom bonded to the oxygen atom of the NO moiety at the M06-2X/6-311++G(d,p) level]  1 2  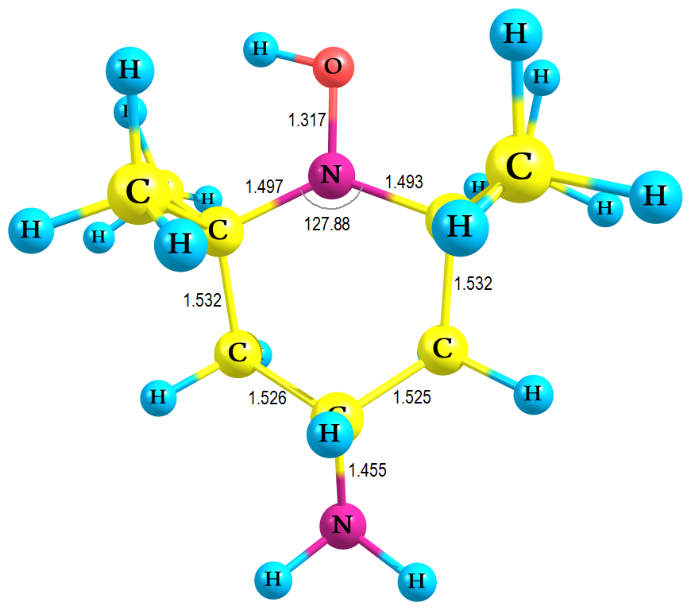 N -0.96588600 0.05767900 -0.21117600  C -0.27523100 1.36487900 -0.00063900  O -2.27442800 0.18825000 -0.28052900  C -0.41584900 -1.31755200 0.00444900  C 1.20322400 1.17827000 -0.35675300  C -0.48583800 1.76923600 1.47058800  C -0.91933900 2.39819700 -0.92908800  C -1.16509200 -2.28718200 -0.91792800  C -0.65402300 -1.69420200 1.47866800  C 1.07105100 -1.29088800 -0.36254100  C 1.83472200 -0.09422700 0.19854300  H 1.73057000 2.06172400 0.01396800  H 1.32326100 1.16423200 -1.44458600  H -0.10364300 2.78454600 1.58903800  H 0.04968200 1.12123300 2.16305500  H -1.54726400 1.77255400 1.72297400  H -0.31791500 3.30684200 -0.87621000  H -1.93727100 2.63984900 -0.62664300  H -0.92125100 2.04979300 -1.96321900  H -0.63028600 -3.23758600 -0.90263600  H -1.18509500 -1.92995400 -1.94945300  H -2.18195100 -2.51506000 -0.57846400  H -0.39033500 -2.74667100 1.59817800  H -1.70281200 -1.57390800 1.75963400  H -0.03898000 -1.10796200 2.15872800  H 1.50369000 -2.22824100 -0.00109800  H 1.18579300 -1.27969400 -1.45100800  H 1.76627100 -0.09258400 1.29623100  N 3.20550000 -0.17111600 -0.28240400  H 3.74950700 0.63103000 0.01772100  H 3.67651700 -0.99973800 0.06543500  H -2.67211500 -0.69152800 -0.40414500  Zero-point correction= 0.293526 (Hartree/Particle)  Thermal correction to Energy= 0.307017  Thermal correction to Enthalpy= 0.307961  Thermal correction to Gibbs Free Energy= 0.255398  Sum of electronic and zero-point Energies= -539.032195  Sum of electronic and thermal Energies= -539.018704  Sum of electronic and thermal Enthalpies= -539.017760  Sum of electronic and thermal Free Energies= -539.070322  **TEMPO-CHO-H^+^**  [H-atom bonded to the oxygen atom of the NO moiety at the M06-2X/6-311++G(d,p) level]  1 2  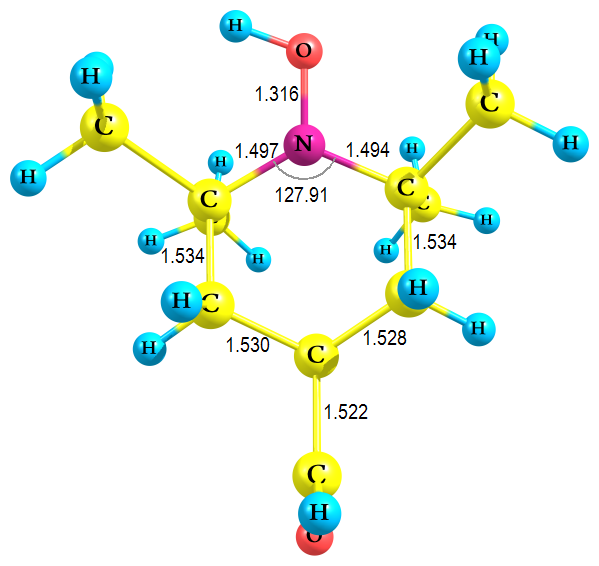  N -1.24194400 0.02243400 -0.18798100  C -0.56686700 1.34535400 -0.00203300  O -2.55799400 0.00465100 -0.19316600  C -0.65944900 -1.34001100 0.00223900  C 0.89212700 1.19744000 -0.45215700  C -0.67763100 1.73069200 1.48441900  C -1.28162900 2.38300800 -0.87605400  C -1.44432100 -2.32178600 -0.87157300  C -0.81090600 -1.70973300 1.48921200  C 0.80964200 -1.29100500 -0.43636100  C 1.56948400 -0.06863200 0.07677500  H 1.42101500 2.09092000 -0.11070100  H 0.92866200 1.19582400 -1.54778000  H -0.33225200 2.76116200 1.58649900  H -0.06123600 1.09960000 2.12177100  H -1.71119700 1.68274900 1.83440900  H -0.66501500 3.28269000 -0.89187200  H -2.25087300 2.69812500 -0.47362000  H -1.39771600 2.03690500 -1.90523200  H -0.92115900 -3.27873900 -0.84154800  H -1.48185600 -1.98412900 -1.90874100  H -2.45841800 -2.47257800 -0.50454500  H -0.52962700 -2.75874100 1.59643500  H -1.84738900 -1.59954900 1.81127900  H -0.16470400 -1.11718800 2.13528700  H 1.27403100 -2.21473300 -0.08232900  H 0.85321300 -1.30756100 -1.53142200  H 1.63159500 -0.05802800 1.16735500  C 3.00511000 -0.09996200 -0.42882400  H 3.12278400 -0.22564300 -1.52577500  O 3.95259100 0.01336700 0.28913100  H -2.88638500 0.91552400 -0.29520200  Zero-point correction= 0.285198 (Hartree/Particle)  Thermal correction to Energy= 0.299594  Thermal correction to Enthalpy= 0.300538  Thermal correction to Gibbs Free Energy= 0.244347  Sum of electronic and zero-point Energies= -596.995063  Sum of electronic and thermal Energies= -596.980668  Sum of electronic and thermal Enthalpies= -596.979724  Sum of electronic and thermal Free Energies= -597.035915  **TEMPO-NO_2_-H^+^**  [H-atom bonded to the oxygen atom of the NO moiety at the M06-2X/6-311++G(d,p) level]  1 2  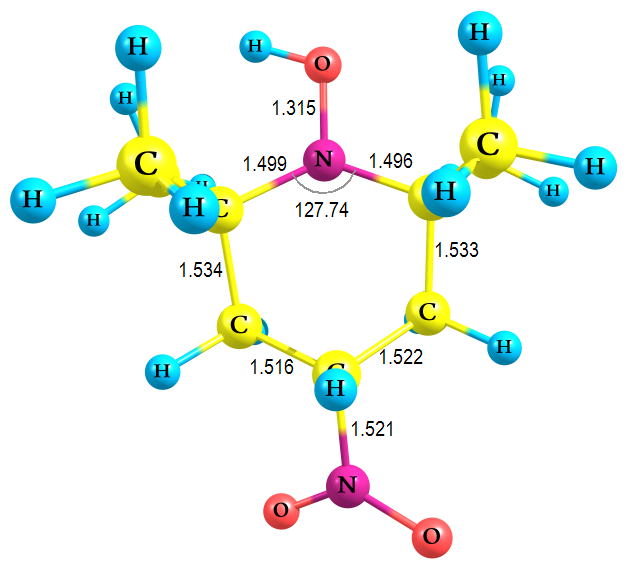  N -1.44488800 0.02896200 -0.24439200  C -0.79488600 1.36287400 -0.05715100  O -2.74854400 0.12627400 -0.38422700  C -0.87665200 -1.32131700 0.07323500  C 0.70152900 1.20459000 -0.34920600  C -1.06385000 1.81952300 1.38851900  C -1.42054600 2.34573400 -1.04994900  C -1.56772700 -2.36034600 -0.81769300  C -1.15868000 -1.61939500 1.55744700  C 0.62418900 -1.29215200 -0.24351000  C 1.29580800 -0.04160500 0.29017600  H 1.20959300 2.09665900 0.01930000  H 0.85965900 1.15384600 -1.43145400  H -0.72461700 2.85319900 1.47364200  H -0.52651300 1.22571300 2.12718500  H -2.13172900 1.79038600 1.60945000  H -0.83928800 3.26815100 -1.01005100  H -2.45311300 2.57733600 -0.79332400  H -1.37860600 1.95753100 -2.06898400  H -1.01367000 -3.29562700 -0.72747500  H -1.55762800 -2.06487200 -1.86884000  H -2.59041900 -2.59160200 -0.49971700  H -0.88439800 -2.65930000 1.74407300  H -2.21816900 -1.50004100 1.79414900  H -0.57918100 -0.98836700 2.22906700  H 1.07080300 -2.19069000 0.18480300  H 0.77456400 -1.34292900 -1.32525900  H 1.27069700 0.01752300 1.38056100  N 2.78325800 -0.06020500 -0.02528200  O 3.23193400 -1.04299800 -0.56218100  O 3.39579300 0.92805000 0.29456300  H -3.12514700 -0.76625700 -0.48586000  Zero-point correction= 0.279385 (Hartree/Particle)  Thermal correction to Energy= 0.294097  Thermal correction to Enthalpy= 0.295041  Thermal correction to Gibbs Free Energy= 0.238222  Sum of electronic and zero-point Energies= -688.175811  Sum of electronic and thermal Energies= -688.161099  Sum of electronic and thermal Enthalpies= -688.160155  Sum of electronic and thermal Free Energies= -688.216974  **TEMPO-H_2_O**  [H-atom bonded to the O-atom of the NO moiety in planar mode at the B3LYP/6-311++G(d,p) level]  0 2  **^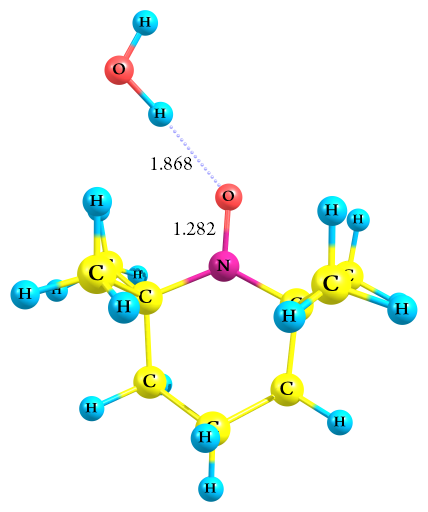^** N 0.10223900 -0.34143500 -0.34287100  C -1.24390200 -0.93880900 -0.02226300  O 1.06096100 -1.18579500 -0.45057100  C 0.48875200 1.09751900 -0.11056000  C -2.35304900 0.11165000 -0.22439100  C -1.22287700 -1.46443300 1.42814000  C -1.46862300 -2.11308600 -0.98692700  C 1.56157600 1.46869500 -1.14687200  C 1.07095400 1.24509400 1.31075600  C -0.74147800 2.00508400 -0.31122400  C -2.01241600 1.48328000 0.35493900  H -3.27385100 -0.28507500 0.21405200  H -2.54041400 0.22342200 -1.29868600  H -2.13344700 -2.03746400 1.62299100  H -1.16968500 -0.65782700 2.16120900  H -0.36336000 -2.12073600 1.57396000  H -2.46517700 -2.53010400 -0.81888700  H -0.72755200 -2.89615300 -0.83224900  H -1.40473900 -1.77928700 -2.02533500  H 1.77501100 2.53806400 -1.06620900  H 1.20603400 1.26533700 -2.15994300  H 2.49021700 0.92314400 -0.98637500  H 1.45997400 2.25914800 1.43705700  H 1.89506300 0.54649200 1.46194800  H 0.32105500 1.07441400 2.08543300  H -0.48286600 3.00122000 0.05995900  H -0.92892400 2.11036300 -1.38622500  H -1.89147000 1.43014400 1.44162100  H -2.83836300 2.17738600 0.17244900  H 2.87368800 -1.09564400 -0.00738700  O 3.78922400 -0.97578000 0.30136100  H 4.20803100 -1.83415600 0.19479900    Zero-point correction= 0.284843 (Hartree/Particle)  Thermal correction to Energy= 0.300427  Thermal correction to Enthalpy= 0.301371  Thermal correction to Gibbs Free Energy= 0.242604  Sum of electronic and zero-point Energies= -560.039944  Sum of electronic and thermal Energies= -560.024360  Sum of electronic and thermal Enthalpies= -560.023416  Sum of electronic and thermal Free Energies= -560.082184  **Description**: In the planar mode, the oxygen atom of the water molecule is positioned between the two geminal methyl groups on one-half of the puckered ring of the TEMPO molecule. This arrangement optimizes dispersively dominated C−H...O interactions, meaning that the interaction between the hydrogen atoms of the methyl groups and the oxygen atom of the water molecule is primarily driven by dispersion forces. The water molecule lies close to the plane of the ring.  **TEMPO-CH_3_-H_2_O**  [H-atom bonded to the O-atom of the NO moiety in planar mode at the B3LYP/6-311++G(d,p) level]  0 2  **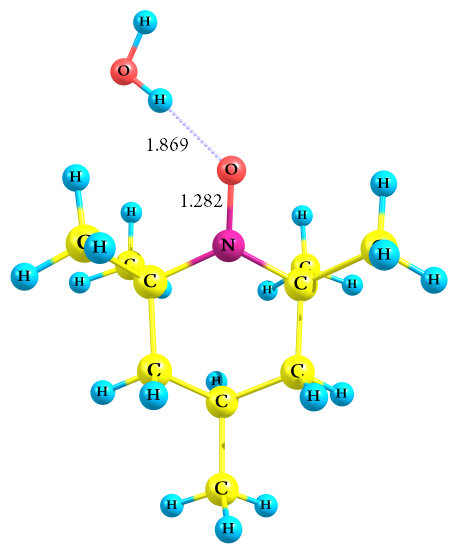** N 0.52240500 0.39790700 -0.33757300  C 0.40668800 -1.08899400 -0.12366100  O 1.70707700 0.88068800 -0.42336000  C -0.55786800 1.39865100 -0.02492300  C -1.05179700 -1.53466600 -0.34573700  C 0.89261300 -1.43904000 1.29830100  C 1.30704100 -1.78317800 -1.15886600  C -0.37450000 2.58888000 -0.97951200  C -0.38426100 1.88071300 1.43053200  C -1.94558000 0.77043000 -0.25043400  C -2.10393300 -0.64211000 0.31681300  H -1.13994600 -2.56577900 0.01176000  H -1.25426100 -1.56046900 -1.42378900  H 0.92094600 -2.52619800 1.41196400  H 0.23329000 -1.03826000 2.07023200  H 1.90033400 -1.05614200 1.46501800  H 1.15318400 -2.86370300 -1.09316500  H 2.36210400 -1.57796800 -0.98380100  H 1.05048900 -1.46102700 -2.17103000  H -1.17927300 3.31024300 -0.81442100  H -0.41387900 2.26019700 -2.02079600  H 0.58172800 3.08273000 -0.81144200  H -1.06414000 2.71484400 1.62384900  H 0.63931600 2.22438200 1.58855500  H -0.60039000 1.09497700 2.15601900  H -2.69149000 1.44660300 0.18024000  H -2.14596000 0.73111700 -1.32840200  H -1.92691800 -0.61848100 1.39866300  C -3.52041100 -1.18162900 0.08889100  H -3.63135100 -2.18867800 0.50135700  H -4.26932400 -0.54123000 0.56401800  H -3.75424800 -1.23191700 -0.97988200  H 3.38304600 0.18420400 0.02202300  O 4.20503500 -0.23753300 0.32933000  H 4.88506200 0.43562300 0.23985700  Zero-point correction= 0.312524 (Hartree/Particle)  Thermal correction to Energy= 0.329605  Thermal correction to Enthalpy= 0.330549  Thermal correction to Gibbs Free Energy= 0.268741  Sum of electronic and zero-point Energies= -599.337947  Sum of electronic and thermal Energies= -599.320866  Sum of electronic and thermal Enthalpies= -599.319922  Sum of electronic and thermal Free Energies= -599.381730  **TEMPO-NH_2_-H_2_O**  [H-atom bonded to the O-atom of the NO moiety in planar mode at the B3LYP/6-311++G(d,p) level]  0 2  **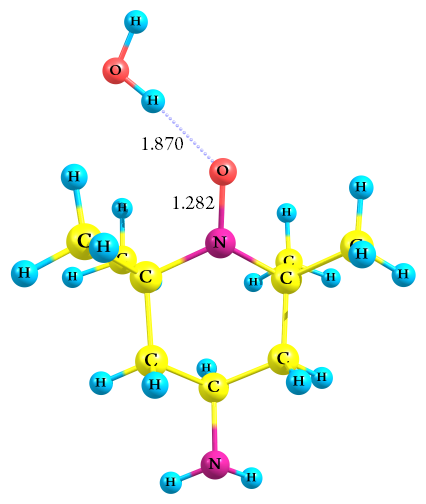** N 0.50727500 0.40373800 -0.33209600  C 0.41358000 -1.08614700 -0.12653900  O 1.68434800 0.90534000 -0.40906800  C -0.59089200 1.38733600 -0.02584900  C -1.03707500 -1.55101000 -0.35549300  C 0.90729900 -1.43648600 1.29303900  C 1.32305100 -1.76121000 -1.16650100  C -0.42603100 2.57565800 -0.98651400  C -0.42612500 1.88024000 1.42725600  C -1.96598200 0.73610900 -0.25606800  C -2.09347400 -0.67486600 0.31326500  H -1.11467800 -2.58427200 0.00027600  H -1.25429100 -1.56971000 -1.42861300  H 0.94283100 -2.52370200 1.40422800  H 0.24937100 -1.04096100 2.06892700  H 1.91355000 -1.04812300 1.45594400  H 1.18760400 -2.84439300 -1.10522400  H 2.37454700 -1.53873900 -0.99109300  H 1.05907300 -1.43923200 -2.17659800  H -1.24130300 3.28588600 -0.82504800  H -0.46090300 2.24100800 -2.02586600  H 0.52286400 3.08392800 -0.81992600  H -1.12664300 2.69757300 1.61869500  H 0.58870500 2.25106200 1.58007800  H -0.61929000 1.09253800 2.15711300  H -2.72607400 1.39591300 0.17684000  H -2.17282600 0.67825800 -1.32967800  H -1.91042800 -0.64829600 1.39780900  N -3.42906000 -1.20270600 -0.00034700  H -3.53689300 -2.14762300 0.35606200  H -4.15239400 -0.63681400 0.43337100  H 3.37256400 0.23021400 0.02995600  O 4.19768800 -0.18638600 0.33543500  H 4.87471300 0.48888500 0.23934300  Zero-point correction= 0.301837 (Hartree/Particle)  Thermal correction to Energy= 0.318806  Thermal correction to Enthalpy= 0.319750  Thermal correction to Gibbs Free Energy= 0.258165  Sum of electronic and zero-point Energies= -615.390398  Sum of electronic and thermal Energies= -615.373429  Sum of electronic and thermal Enthalpies= -615.372485  Sum of electronic and thermal Free Energies= -615.434071  **TEMPO-CHO-H_2_O**  [H-atom bonded to the O-atom of the NO moiety in planar mode at the B3LYP/6-311++G(d,p) level]  0 2  **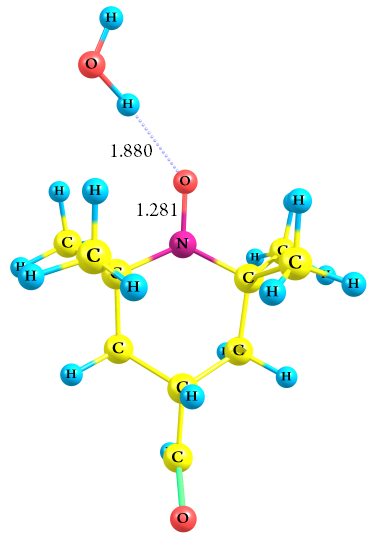** N -0.82872000 0.40003300 -0.34301200  C 0.13367100 1.51237500 -0.02573600  O -2.06429100 0.73816200 -0.36622900  C -0.52923400 -1.06683300 -0.18398100  C 1.57599900 1.07533400 -0.34465700  C -0.02198100 1.90649800 1.45734400  C -0.23428400 2.71126200 -0.91395000  C -1.39043100 -1.83597200 -1.19942500  C -0.89245600 -1.51734400 1.24619200  C 0.95803900 -1.33143200 -0.49590300  C 1.92269100 -0.33326900 0.16108000  H 2.25370800 1.81851300 0.08574900  H 1.72236100 1.10252700 -1.43098300  H 0.56147700 2.80835900 1.66053000  H 0.32185300 1.12368400 2.13486800  H -1.07028700 2.11618300 1.67548000  H 0.48849200 3.51501000 -0.75144100  H -1.23092800 3.08057900 -0.67713000  H -0.21232500 2.43370300 -1.97041700  H -1.09958100 -2.88972500 -1.18435300  H -1.23495900 -1.44992500 -2.20971400  H -2.45073300 -1.77241000 -0.96007000  H -0.77954200 -2.60205800 1.32197900  H -1.93068500 -1.27069100 1.47190400  H -0.25148200 -1.06048100 2.00188100  H 1.18613000 -2.35342800 -0.17995300  H 1.10367800 -1.29495400 -1.58210600  H 1.86277400 -0.38582100 1.25066000  C 3.34915600 -0.67258100 -0.21502800  H 3.55639500 -0.67121000 -1.30972200  O 4.22799800 -0.92935100 0.56930300  H -3.62731900 -0.17158300 0.14772200  O -4.34612200 -0.73135500 0.48873000  H -5.13569100 -0.18437400 0.45615300  Zero-point correction= 0.293714 (Hartree/Particle)  Thermal correction to Energy= 0.311478  Thermal correction to Enthalpy= 0.312422  Thermal correction to Gibbs Free Energy= 0.248081  Sum of electronic and zero-point Energies= -673.379753  Sum of electronic and thermal Energies= -673.361990  Sum of electronic and thermal Enthalpies= -673.361046  Sum of electronic and thermal Free Energies= -673.425386  **TEMPO-NO_2_-H_2_O**  [H-atom bonded to the O-atom of the NO moiety in planar mode at the B3LYP/6-311++G(d,p) level]  0 2  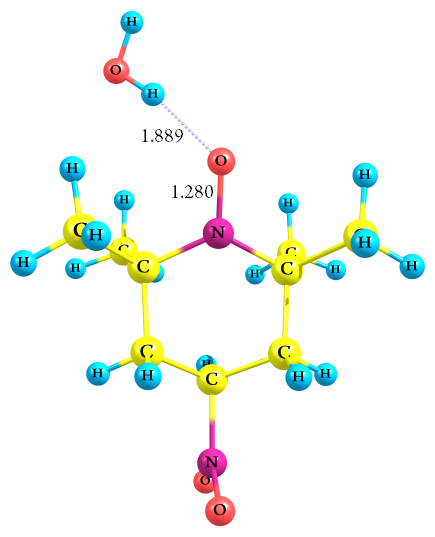 N 1.06313900 0.39782400 -0.34862600  C 0.71365700 -1.05315300 -0.14096700  O 2.30750200 0.68354200 -0.44537200  C 0.16606000 1.55668000 -0.00218700  C -0.79775700 -1.26042800 -0.37401800  C 1.13448500 -1.49271600 1.27678400  C 1.48274400 -1.87801000 -1.18589400  C 0.52801700 2.71988100 -0.93855100  C 0.42307300 1.97582900 1.45987700  C -1.30917900 1.17526700 -0.23555300  C -1.64650500 -0.20256200 0.32142000  H -1.06111400 -2.26057200 -0.01998400  H -1.01658800 -1.22191100 -1.44399700  H 0.97036400 -2.56815800 1.38359900  H 0.56466300 -0.98815000 2.05920300  H 2.19533700 -1.29685800 1.43697100  H 1.15073500 -2.91802300 -1.13165200  H 2.55592800 -1.85340700 -1.00446600  H 1.28635900 -1.50565600 -2.19385100  H -0.14904300 3.55741100 -0.75248000  H 0.43089900 2.42125300 -1.98462300  H 1.55172400 3.04958300 -0.76820500  H -0.11407700 2.90292600 1.67599600  H 1.48940200 2.15083200 1.61004100  H 0.09558200 1.22229000 2.17804500  H -1.93759700 1.94331800 0.22361900  H -1.52588600 1.17051100 -1.30651600  H -1.55894100 -0.24661400 1.40478100  N -3.12279700 -0.49817600 0.07392900  H 3.85816600 -0.28647500 0.02745000  O 4.53661300 -0.89280200 0.37010100  H 5.36966600 -0.41936600 0.29522500  O -3.51139300 -0.50073100 -1.08431600  O -3.82091300 -0.71440700 1.05312000  Zero-point correction= 0.287750 (Hartree/Particle)  Thermal correction to Energy= 0.305985  Thermal correction to Enthalpy= 0.306930  Thermal correction to Gibbs Free Energy= 0.241114  Sum of electronic and zero-point Energies= -764.600829  Sum of electronic and thermal Energies= -764.582594  Sum of electronic and thermal Enthalpies= -764.581650  Sum of electronic and thermal Free Energies= -764.647466  **TEMPO-H_2_O**  [H-atom bonded to the O-atom of the NO moiety in top mode at the B3LYP/6-311++G(d,p) level]  0 2  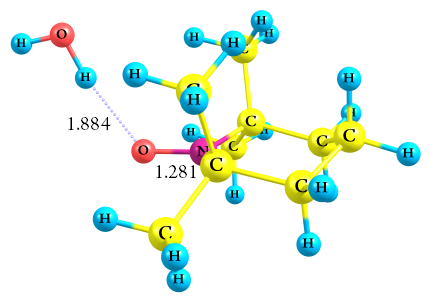 N 0.17704500 0.00008400 0.55869700  C -0.36142600 1.33503300 0.11246600  O 1.36641600 0.00038000 1.03443700  C -0.36075300 -1.33514500 0.11250200  C -1.88927300 1.24591600 -0.07093800  C 0.34112300 1.75459400 -1.19618600  C -0.03688500 2.35445500 1.21446200  C -0.03565500 -2.35437500 1.21451200  C 0.34197900 -1.75436900 -1.19616100  C -1.88864600 -1.24679900 -0.07086100  C -2.34782900 -0.00058000 -0.82580300  H -2.21906900 2.15674900 -0.57985500  H -2.36330700 1.25004700 0.91765600  H 0.06889100 2.78582700 -1.43768000  H 0.05566000 1.12629600 -2.04133900  H 1.42522300 1.70159100 -1.08415800  H -0.47768800 3.31923700 0.94965800  H 1.03868200 2.47967500 1.33102700  H -0.45228500 2.03581600 2.17349200  H -0.47596600 -3.31939000 0.94974000  H -0.45119300 -2.03593200 2.17354800  H 1.03998000 -2.47903800 1.33105100  H 0.07027200 -2.78574800 -1.43761800  H 1.42605400 -1.70079600 -1.08416700  H 0.05616200 -1.12624300 -2.04132300  H -2.21800700 -2.15782700 -0.57970900  H -2.36265200 -1.25109500 0.91774700  H -1.96174100 -0.00051700 -1.84992500  H -3.43879200 -0.00085800 -0.91025600  H 2.93839900 0.00058300 -0.00442000  O 3.63649200 0.00061600 -0.68163000  H 4.46300500 0.00068000 -0.19075500  Zero-point correction= 0.284667 (Hartree/Particle)  Thermal correction to Energy= 0.300322  Thermal correction to Enthalpy= 0.301266  Thermal correction to Gibbs Free Energy= 0.241550  Sum of electronic and zero-point Energies= -560.039435  Sum of electronic and thermal Energies= -560.023779  Sum of electronic and thermal Enthalpies= -560.022835  Sum of electronic and thermal Free Energies= -560.082552  **Description**: In the top mode, the oxygen atom of the water molecule remains in or near the symmetry plane of the TEMPO molecule. It aligns with the two axial methyl groups on one face of the ring. This arrangement forms hydrogen bonds between the hydrogen atoms of the methyl groups and the oxygen atom of the water molecule, with the water molecule positioned above the ring.  **TEMPO-CH_3_-H_2_O**  [H-atom bonded to the O-atom of the NO moiety in top mode at the B3LYP/6-311++G(d,p) level]  0 2  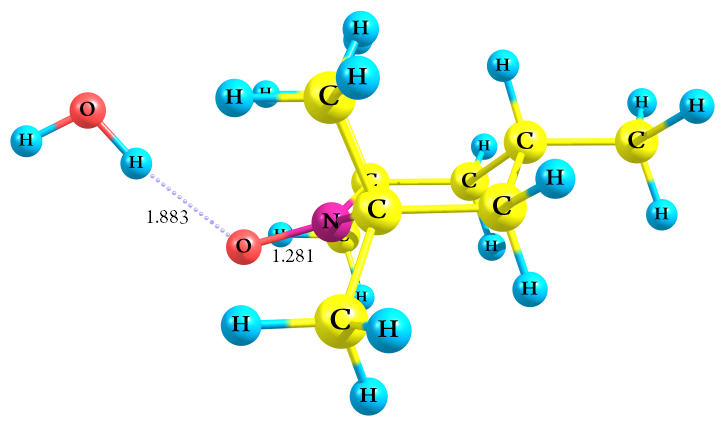 N 0.55360600 0.00004900 0.57843800  C -0.03068900 1.33198800 0.18985600  O 1.78537200 0.00011000 0.93007300  C -0.03057900 -1.33195100 0.18989200  C -1.56855700 1.23958400 0.17011700  C 0.53189200 1.75339100 -1.18432000  C 0.40542200 2.35413800 1.25058800  C 0.40561600 -2.35403400 1.25065200  C 0.53203300 -1.75334900 -1.18427300  C -1.56845400 -1.23967200 0.17015300  C -2.12532500 -0.00007700 -0.53525900  H -1.95152800 2.15281700 -0.29707300  H -1.93538900 1.23964100 1.20421300  H 0.22893900 2.78205800 -1.39847400  H 0.16736500 1.12119600 -1.99517300  H 1.62220000 1.70879400 -1.18138600  H -0.06193200 3.31790900 1.03189400  H 1.48722700 2.48066800 1.25298600  H 0.09356200 2.03668300 2.24842700  H -0.06166000 -3.31784900 1.03198700  H 0.09373400 -2.03657600 2.24848300  H 1.48743200 -2.48047900 1.25305100  H 0.22915100 -2.78204200 -1.39840100  H 1.62233800 -1.70867700 -1.18134400  H 0.16746000 -1.12120000 -1.99514200  H -1.95135200 -2.15295000 -0.29700700  H -1.93528300 -1.23972700 1.20425000  H -1.79128400 -0.00007800 -1.57916300  C -3.65818300 -0.00014000 -0.53866200  H -4.05256000 0.88373200 -1.04844100  H -4.05248700 -0.88405700 -1.04842000  H -4.05329300 -0.00014500 0.48283000  H 3.25154600 -0.00002000 -0.25219100  O 3.89046200 -0.00002400 -0.98540200  H 4.75484900 -0.00000100 -0.56480000  Zero-point correction= 0.311866 (Hartree/Particle)  Thermal correction to Energy= 0.328254  Thermal correction to Enthalpy= 0.329198  Thermal correction to Gibbs Free Energy= 0.269524  Sum of electronic and zero-point Energies= -599.337841  Sum of electronic and thermal Energies= -599.321454  Sum of electronic and thermal Enthalpies= -599.320510  Sum of electronic and thermal Free Energies= -599.380183  **TEMPO-NH_2_-H_2_O**  [H-atom bonded to the O-atom of the NO moiety in top mode at the B3LYP/6-311++G(d,p) level]  0 2  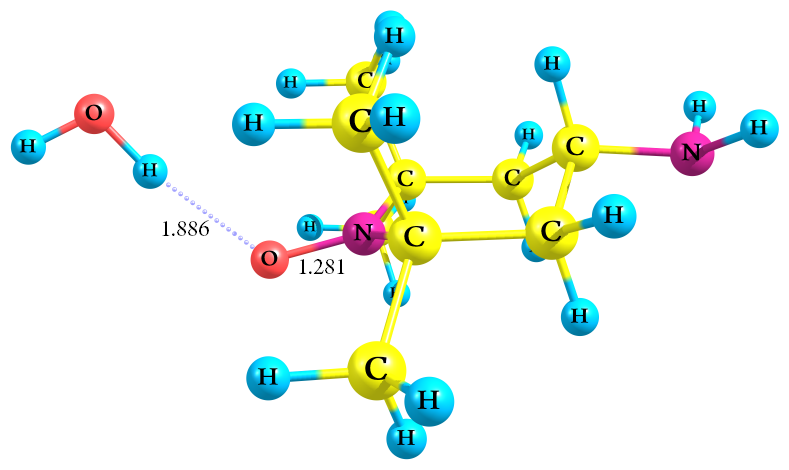 N -0.54276500 -0.00005000 0.57816100  C 0.04298100 -1.33312400 0.19395500  O -1.77510600 -0.00011000 0.92647000  C 0.04286400 1.33308800 0.19399100  C 1.57969400 -1.23744200 0.16881200  C -0.52921700 -1.76423100 -1.17353900  C -0.38359800 -2.34937800 1.26431700  C -0.38380900 2.34928200 1.26437300  C -0.52936600 1.76417500 -1.17349600  C 1.57958400 1.23754000 0.16885300  C 2.12029500 0.00008400 -0.54438100  H 1.96452100 -2.14904700 -0.30161400  H 1.9651 6500 -1.22266200 1.19367500  H -0.21672300 -2.78980200 -1.38881900  H -0.18197700 -1.12933100 -1.98994200  H -1.61983400 -1.73242300 -1.15834700  H 0.08008800 -3.31506300 1.04656200  H -1.46560600 -2.47361300 1.27664800  H -0.06142600 -2.02696100 2.25708700  H 0.07979700 3.31501100 1.04664000  H -0.06161700 2.02687300 2.25713900  H -1.46582700 2.47342400 1.27669600  H -0.21695800 2.78977800 -1.38875300  H -1.61998000 1.73226700 -1.15831300  H -0.18206000 1.12932500 -1.98991100  H 1.96433600 2.14919400 -0.30154100  H 1.96505200 1.22275800 1.19371700  H 1.77033600 0.00008600 -1.58681000  N 3.58844800 0.00014700 -0.46471300  H 3.97452200 -0.81619200 -0.92967900  H 3.97445400 0.81651700 -0.92968300  H -3.24138800 -0.00007500 -0.26028900  O -3.87713400 0.00000800 -0.99607000  H -4.74330700 0.00010400 -0.57909600  Zero-point correction= 0.301285 (Hartree/Particle)  Thermal correction to Energy= 0.317521  Thermal correction to Enthalpy= 0.318466  Thermal correction to Gibbs Free Energy= 0.259130  Sum of electronic and zero-point Energies= -615.390221  Sum of electronic and thermal Energies= -615.373985  Sum of electronic and thermal Enthalpies= -615.373041  Sum of electronic and thermal Free Energies= -615.432376  **TEMPO-CHO-H_2_O**  [H-atom bonded to the O-atom of the NO moiety in top mode at the B3LYP/6-311++G(d,p) level]  0 2  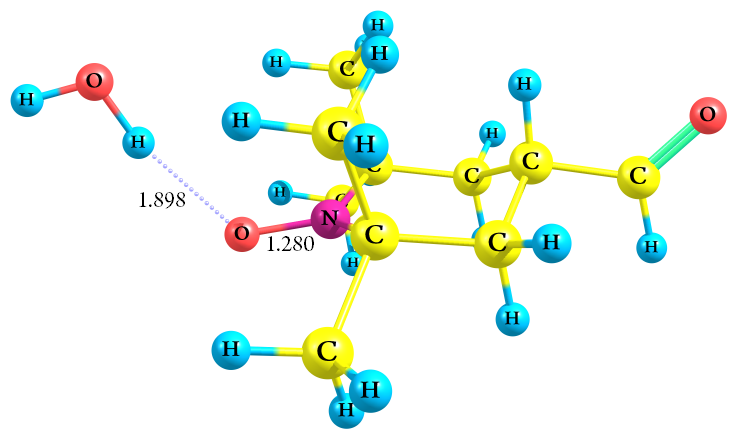 N 0.85224400 0.00005600 -0.59895800  C 0.24103100 -1.33162200 -0.25456800  O 2.10732700 0.00010300 -0.85075500  C 0.24094600 1.33168000 -0.25451600  C -1.29288900 -1.24678900 -0.37980000  C 0.67006400 -1.74489700 1.16893400  C 0.77140500 -2.35943100 -1.26570900  C 0.77124700 2.35956600 -1.26561600  C 0.66996500 1.74492100 1.16900200  C -1.29297000 1.24675700 -0.37974500  C -1.90077800 -0.00005100 0.28099500  H -1.71567300 -2.15777200 0.05417600  H -1.56141700 -1.24570500 -1.44302700  H 0.36300200 -2.77807900 1.35227300  H 0.21709200 -1.12042100 1.94007400  H 1.75422400 -1.68249500 1.27438900  H 0.28311300 -3.32114100 -1.08862700  H 1.84777200 -2.48846500 -1.16306100  H 0.55884100 -2.04495400 -2.29016900  H 0.28288700 3.32123400 -1.08849300  H 0.55870300 2.04511600 -2.29008900  H 1.84760500 2.48866700 -1.16296100  H 0.36286500 2.77808500 1.35238300  H 1.75412800 1.68254900 1.27444700  H 0.21701800 1.12039800 1.94012000  H -1.71580900 2.15769300 0.05427700  H -1.56150200 1.24570600 -1.44297100  H -1.72121700 -0.00006800 1.35852900  C -3.40214900 -0.00009100 0.08694600  H -3.73520500 -0.00010300 -0.97637300  O -4.21457400 -0.00010700 0.97768900  H 3.45360800 -0.00011000 0.48747500  O 3.95806200 -0.00008800 1.31814100  H 4.88250600 -0.00004200 1.05419800  Zero-point correction= 0.293274 (Hartree/Particle)  Thermal correction to Energy= 0.311207  Thermal correction to Enthalpy= 0.312151  Thermal correction to Gibbs Free Energy= 0.246185  Sum of electronic and zero-point Energies= -673.379470  Sum of electronic and thermal Energies= -673.361537  Sum of electronic and thermal Enthalpies= -673.360593  Sum of electronic and thermal Free Energies= -673.426559  **TEMPO-NO_2_-H_2_O**  [H-atom bonded to the O-atom of the NO moiety in top mode at the B3LYP/6-311++G(d,p) level]  0 2  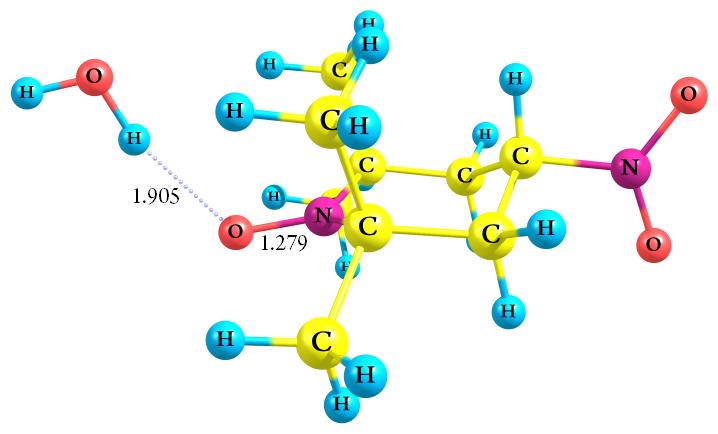 N 1.07049900 0.00904900 -0.60626900  C 0.48050200 -1.32711200 -0.23849700  O 2.31242000 0.01509400 -0.91302800  C 0.47092100 1.33793900 -0.22805100  C -1.05842700 -1.24809800 -0.30053300  C 0.97562600 -1.74550600 1.16204800  C 0.96483300 -2.34950600 -1.27746000  C 0.94730500 2.37199000 -1.25912800  C 0.96429900 1.74847300 1.17539900  C -1.06749600 1.24865000 -0.28974100  C -1.60327300 -0.00461 700 0.39328200  H -1.46637900 -2.15417300 0.15561000  H -1.38542200 -1.22658500 -1.34300400  H 0.67238500 -2.77731600 1.35828700  H 0.56777100 -1.12076300 1.95817500  H 2.06378100 -1.68846200 1.21403600  H 0.48934900 -3.31372300 -1.08114000  H 2.04529000 -2.47374200 -1.22421300  H 0.70312300 -2.03208200 -2.28914800  H 0.46497800 3.33118700 -1.05498600  H 0.68727900 2.06069000 -2.27314900  H 2.02688200 2.50349800 -1.20551800  H 0.65452100 2.77662200 1.38037000  H 2.05289500 1.69816400 1.22532000  H 0.56167200 1.11440300 1.96677600  H -1.48142300 2.14784700 0.17456800  H -1.39497000 1.23407100 -1.33216500  H -1.40943400 -0.00860900 1.46365100  N -3.12702400 -0.00987900 0.30988000  H 3.71558800 -0.00637100 0.37507100  O 4.21803500 -0.02060000 1.20633200  H 5.14362000 -0.01831000 0.94639500  O -3.63127300 -0.00552800 -0.80299000  O -3.74437700 -0.01819300 1.36417100  Zero-point correction= 0.287311 (Hartree/Particle)  Thermal correction to Energy= 0.304808  Thermal correction to Enthalpy= 0.305752  Thermal correction to Gibbs Free Energy= 0.242138  Sum of electronic and zero-point Energies= -764.600735  Sum of electronic and thermal Energies= -764.583238  Sum of electronic and thermal Enthalpies= -764.582294  Sum of electronic and thermal Free Energies= -764.645908  **TEMPO-H_2_O**  [H-atom bonded to the O-atom of the NO moiety in orthogonal mode at the B3LYP/6-311++G(d,p) level]  0 2  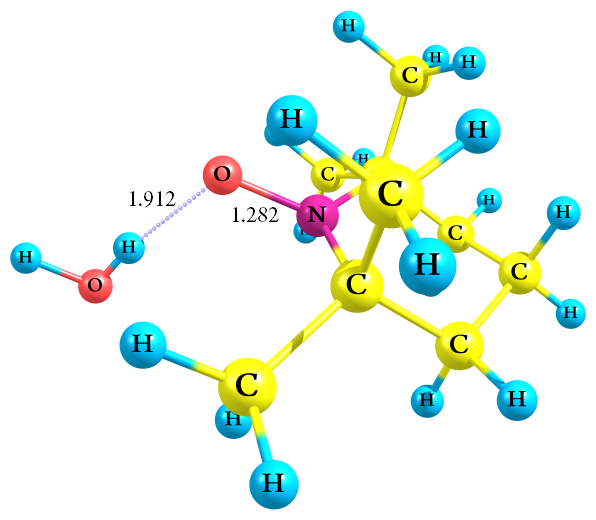 N -0.31723600 0.00018400 0.23934200  C 0.36126800 1.33553300 0.07720900  O -1.39989700 0.00059100 0.92561300  C 0.36031300 -1.33566600 0.07731900  C 1.44457500 1.24494900 -1.01488600  C 0.95850500 1.76071600 1.43470700  C -0.71033100 2.35063500 -0.34778500  C -0.71202000 -2.35001600 -0.34760400  C 0.95721900 -1.76114800 1.43487000  C 1.44371300 -1.24594200 -1.01476700  C 2.32286800 -0.00079800 -0.91070100  H 2.04718900 2.15705900 -0.96550800  H 0.95416400 1.24577800 -1.99502800  H 1.31340500 2.79284000 1.36992000  H 1.80012600 1.13465800 1.73582200  H 0.19223400 1.70730100 2.21002800  H -0.23055700 3.31142700 -0.55310500  H -1.45382300 2.49163400 0.43581200  H -1.22606200 2.01945600 -1.25163800  H -0.23296000 -3.31121400 -0.55269000  H -1.22739200 -2.01862000 -1.25158200  H -1.45572500 -2.49028700 0.43592000  H 1.31136900 -2.79353500 1.37019100  H 0.19097200 -1.70710400 2.21017200  H 1.79929200 -1.13567100 1.73593800  H 2.04568700 -2.15846700 -0.96527900  H 0.95331800 -1.24653900 -1.99491700  H 2.89249200 -0.00095100 0.02432100  H 3.06083900 -0.00109300 -1.71842700  H -3.03208700 0.00063400 -0.07101100  O -3.78427600 0.00031000 -0.68665500  H -4.56510200 0.00461000 -0.12617200    Zero-point correction= 0.284272 (Hartree/Particle)  Thermal correction to Energy= 0.299282  Thermal correction to Enthalpy= 0.300226  Thermal correction to Gibbs Free Energy= 0.242213  Sum of electronic and zero-point Energies= -560.039755  Sum of electronic and thermal Energies= -560.024745  Sum of electronic and thermal Enthalpies= -560.023801  Sum of electronic and thermal Free Energies= -560.081813  **Description**: In the orthogonal mode, the oxygen atom of the water molecule points towards the opposite, open side of the ring, framed by the two equatorial methyl groups of the TEMPO molecule. The water molecule lies perpendicular to the plane of the ring. Hydrogen bonding occurs between the hydrogen atoms of the equatorial methyl groups and the oxygen atom of the water molecule.  **TEMPO-CH_3_-H_2_O**  [H-atom bonded to the O-atom of the NO moiety in orthogonal mode at the B3LYP/6-311++G(d,p) level]  0 2  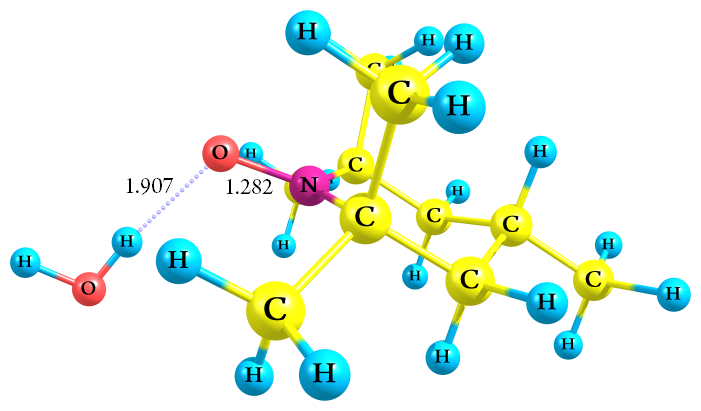 N -0.65883600 -0.00000800 0.26733200  C 0.03841200 1.33379600 0.24717100  O -1.86165200 -0.00001300 0.71060600  C 0.03843400 -1.33380200 0.24716400  C 1.33552100 1.23803300 -0.57831600  C 0.31972700 1.77160600 1.69976400  C -0.91307800 2 .34375500 -0.41248900  C -0.91303100 -2.34377900 -0.41250500  C 0.31975700 -1.77161700 1.69975500  C 1.33554400 -1.23801400 -0.57831900  C 2.18854400 0.00001700 -0.28972500  H 1.91032400 2.15302300 -0.40110400  H 1.07615700 1.23445900 -1.64424900  H 0.68615200 2.80169600 1.70603900  H 1.06893600 1.14509300 2.18651800  H -0.60077900 1.72791600 2.28433600  H -0.40363000 3.30656700 -0.50565200  H -1.81529600 2.48088100 0.18240500  H -1.20908700 2.00929500 -1.40905400  H -0.40355700 -3.30657500 -0.50568700  H -1.20905700 -2.00931100 -1.40906200  H -1.81524000 -2.48094000 0.18239500  H 0.68620700 -2.80169900 1.70602300  H -0.60075200 -1.72795500 2.28432400  H 1.06894900 -1.14508900 2.18651700  H 1.91036300 -2.15299400 -0.40110800  H 1.07618100 -1.23444300 -1.64425200  H 2.47789300 0.00001900 0.76799300  C 3.47432700 0.00003000 -1.12396400  H 4.08286200 0.88408800 -0.91263500  H 4.08287800 -0.88401700 -0.91263700  H 3.24699400 0.00002900 -2.19528600  H -3.26158700 -0.00002100 -0.58454700  O -3.89186100 -0.00000500 -1.32460200  H -4.75989600 0.00002400 -0.91184700  Zero-point correction= 0.311988 (Hartree/Particle)  Thermal correction to Energy= 0.329335  Thermal correction to Enthalpy= 0.330279  Thermal correction to Gibbs Free Energy= 0.266450  Sum of electronic and zero-point Energies= -599.337499  Sum of electronic and thermal Energies= -599.320152  Sum of electronic and thermal Enthalpies= -599.319208  Sum of electronic and thermal Free Energies= -599.383037  **TEMPO-NH_2_-H_2_O**  [H-atom bonded to the O-atom of the NO moiety in orthogonal mode at the B3LYP/6-311++G(d,p) level]  0 2  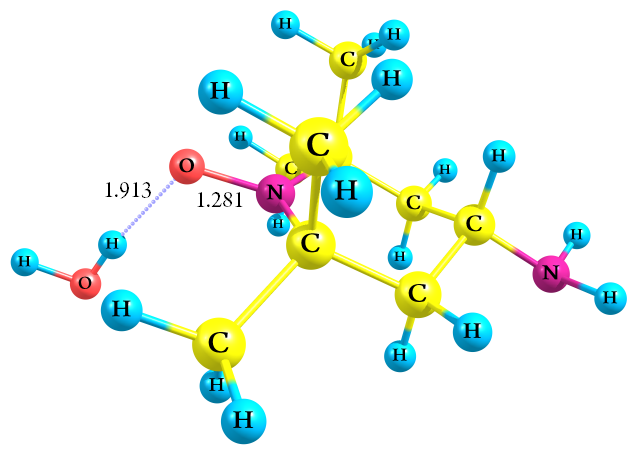 N -0.64411300 -0.00026100 0.26564500  C 0.05240600 1.33443900 0.23695100  O -1.84138900 -0.00058900 0.72205400  C 0.05299500 -1.33463700 0.23667900  C 1.33956800 1.23675100 -0.60170300  C 0.34238000 1.77661300 1.68682800  C -0.90529700 2.34129900 -0.41878300  C -0.90428200 -2.34177300 -0.41925400  C 0.34316900 -1.77698100 1.68646400  C 1.34010900 -1.23621600 -0.60196300  C 2.18687300 0.00042100 -0.30897600  H 1.92093400 2.14958200 -0.43042700  H 1.08527800 1.21940200 -1.66646200  H 0.71462100 2.80466600 1.68775100  H 1.08930200 1.14758100 2.17416600  H -0.57652100 1.74164000 2.27462600  H -0.39817 500 3.30448300 -0.52021900  H -1.80213300 2.47927300 0.18398100  H -1.20899500 2.00241400 -1.41139100  H -0.39675000 -3.30471900 -0.52089900  H -1.20813300 -2.00280200 -1.41178600  H -1.80104900 -2.48025500 0.18350000  H 0.71583300 -2.80488200 1.68717700  H -0.57573700 -1.74250000 2.27428200  H 1.08984000 -1.14774000 2.17391500  H 1.92187100 -2.14883100 -0.43087900  H 1.08580600 -1.21875400 -1.66671900  H 2.47394700 0.00037000 0.75328600  N 3.36260700 0.00077100 -1.19035500  H 3.94174600 0.81772400 -1.02136200  H 3.94201600 -0.81605100 -1.02165000  H -3.27253600 -0.00006700 -0.54703800  O -3.91868400 0.00031900 -1.27292900  H -4.77756700 -0.00143200 -0.84133400  Zero-point correction= 0.301180 (Hartree/Particle)  Thermal correction to Energy= 0.317580  Thermal correction to Enthalpy= 0.318524  Thermal correction to Gibbs Free Energy= 0.257737  Sum of electronic and zero-point Energies= -615.389961  Sum of electronic and thermal Energies= -615.373561  Sum of electronic and thermal Enthalpies= -615.372617  Sum of electronic and thermal Free Energies= -615.433404  **TEMPO-CHO-H_2_O**  [H-atom bonded to the O-atom of the NO moiety in orthogonal mode at the B3LYP/6-311++G(d,p) level]  0 2  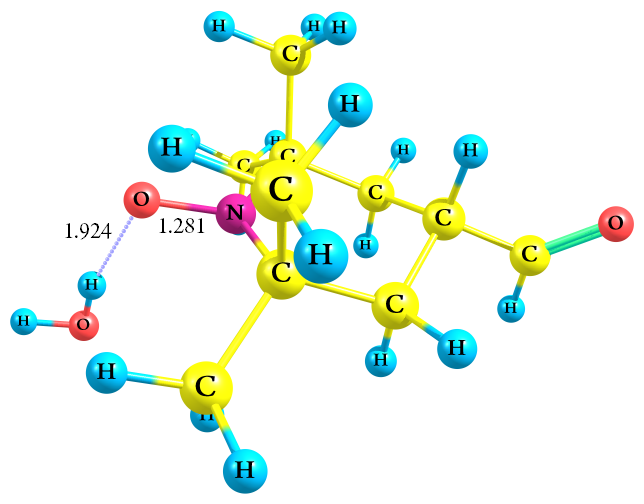 N 0.93903900 0.00002100 0.29489500  C 0.24219700 -1.33325700 0.30648100  O 2.15901800 0.00004600 0.68621300  C 0.24215900 1.33328000 0.30645300  C -1.07592200 -1.24521400 -0.48762100  C -0.00376400 -1.76241900 1.76747400  C 1.17142000 -2.34970000 -0.37487900  C 1.17135900 2.34973900 -0.37491800  C -0.00382300 1.76245600 1.76743800  C -1.07595500 1.24518400 -0.48765100  C -1.91468100 -0.00002200 -0.16382400  H -1.64646400 -2.15745000 -0.28957800  H -0.84325200 -1.24188600 -1.55897500  H -0.36572000 -2.79364 800 1.78934400  H -0.74260700 -1.13652200 2.27007100  H 0.93133100 -1.71199800 2.32754500  H 0.65338200 -3.30878300 -0.45703700  H 2.08337500 -2.49371300 0.20302200  H 1.45178700 -2.01594100 -1.37603500  H 0.65330400 3.30881200 -0.45707500  H 1.45172200 2.01598100 -1.37607600  H 2.08331800 2.49376900 0.20297400  H -0.36579800 2.79367800 1.78929000  H 0.93126700 1.71205800 2.32751900  H -0.74266000 1.13655100 2.27003500  H -1.64652000 2.15740900 -0.28963100  H -0.84328200 1.24183600 -1.55900500  H -2.23290600 -0.00001200 0.88138300  C -3.17462600 -0.00004600 -1.00319900  H -3.00277100 -0.00006200 -2.10382700  O -4.29718000 -0.00003500 -0.56316700  H 3.41721000 -0.00001100 -0.76968700  O 3.87786500 -0.00003900 -1.62496200  H 4.81419700 0.00001300 -1.40770400  Zero-point correction= 0.293220 (Hartree/Particle)  Thermal correction to Energy= 0.311253  Thermal correction to Enthalpy= 0.312197  Thermal correction to Gibbs Free Energy= 0.245690  Sum of electronic and zero-point Energies= -673.379457  Sum of electronic and thermal Energies= -673.361424  Sum of electronic and thermal Enthalpies= -673.360480  Sum of electronic and thermal Free Energies= -673.426987  **TEMPO-NO_2_-H_2_O**  [H-atom bonded to the O-atom of the NO moiety in orthogonal mode at the B3LYP/6-311++G(d,p) level]  0 2  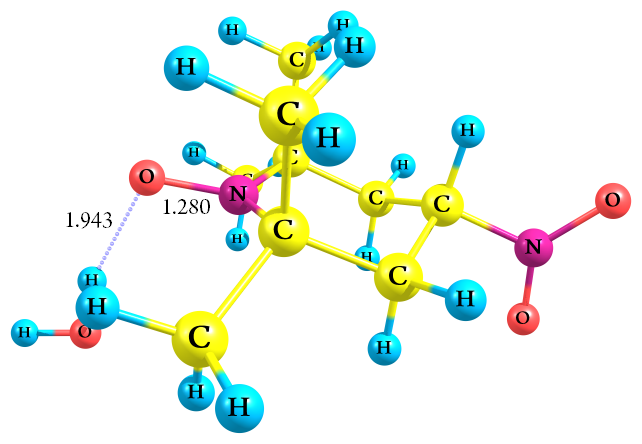 N 1.18833800 0.00004300 -0.32374100  C 0.49494600 1.33438600 -0.39739400  O 2.43856000 0.00003300 -0.59963200  C 0.49505800 -1.33433100 -0.39744200  C -0.88318300 1.24698700 0.28833700  C 0.37321800 1.76193600 -1.87464900  C 1.36057500 2.35260900 0.36056400  C 1.36084200 -2.35240500 0.36052500  C 0.37344200 -1.76187600 -1.8747 1000  C -0.88308600 -1.24706300 0.28828500  C -1.65638800 -0.00005800 -0.12350100  H -1.44641600 2.15069100 0.03988200  H -0.75465900 1.22628100 1.37312700  H 0.00905100 2.79104300 -1.92909200  H -0.31395100 1.13230000 -2.44275500  H 1.35387300 1.71745700 -2.35103700  H 0.83233300 3.30845500 0.40587000  H 2.31313900 2.50374200 -0.14547900  H 1.56360000 2.01430600 1.37840900  H 0.83285600 -3.30840000 0.40560700  H 1.56358900 -2.01417700 1.37845000  H 2.31354700 -2.50320600 -0.14536400  H 0.00940300 -2.79102700 -1.92917400  H 1.35410900 -1.71727700 -2.35106500  H -0.31378000 -1.13232100 -2.44283800  H -1.44623900 -2.15080300 0.03978300  H -0.75457600 -1.22639700 1.37307900  H -1.91142800 -0.00004300 -1.18102200  N -3.01790300 -0.00011500 0.56583400  H 3.48515300 0.00000200 1.03701000  O 3.74999700 0.00000800 1.97099500  H 4.71120600 -0.00018300 1.96417600  O -3.03250800 -0.00009900 1.78697300  O -4.00684900 -0.00007200 -0.15237500  Zero-point correction= 0.287446 (Hartree/Particle)  Thermal correction to Energy= 0.305869  Thermal correction to Enthalpy= 0.306813  Thermal correction to Gibbs Free Energy= 0.239692  Sum of electronic and zero-point Energies= -764.600099  Sum of electronic and thermal Energies= -764.581676  Sum of electronic and thermal Enthalpies= -764.580731  Sum of electronic and thermal Free Energies= -764.647853 |
| --- |

**Table S2.** Calculated Mulliken atomic charges of TEMPO derivatives and their protonated forms at the B3LYP/6-311++G(d,p) and M06-2X/6-311++G(d,p) (in parenthesis) levels of theory.

| species  atom charge | TEMPO-CH_3_ | TEMPO | TEMPO-NH_2_ | TEMPO-CHO | TEMPO-NO_2_ |
| --- | --- | --- | --- | --- | --- |
| N_1_ | 0.2014  (−0.0034) | 0.2495  (0.0560) | 0.2426  (0.0642) | 0.1970  (−0.0140) | 0.2310  (0.0189) |
| C_2_ | 0.1788  (−0.0558) | −0.0566  (−0.2428) | 0.0514  (−0.1554) | 0.1797  (−0.0508) | −0.0760  (−0.1961) |
| O_3_ | −0.0502  (0.0351) | −0.0758  (0.0049) | −0.0734  (0.0041) | −0.0433  (0.0451) | −0.0437  (0.0454) |
| C_4_ | 0.1788  (−0.0558) | −0.0566  (−0.2428) | 0.0514  (−0.1554) | 0.1797  (−0.0508) | −0.0759  (−0.1961) |
| C_5_ | −0.8737  (−0.6858) | −0.3280  (-0.3368) | −0.5341  (−0.4041) | −0.7984  (−0.5968) | −0.5604  (−0.4749) |
| C_10_ | −0.8737  (−0.6858) | −0.3280  (−0.3368) | −0.5341  (−0.4041) | −0.7984  (−0.5968) | −0.5604  (−0.4749) |
| C_11_ | 0.7763  (0.5048) | −0.3484  (−0.3074) | −0.2749  (−0.4530) | 0.3576  (0.0231) | −0.0785  (−0.2683) |
|  | O-protonation | | | | |
| N_1_ | 0.1613  (−0.0005) | 0.2012  (0.0438) | 0.1960  (0.0481) | 0.1552  (−0.0097) | 0.1693  (0.0824) |
| C_2_ | 0.0459  (−0.0995) | −0.4089  (−0.5271) | −0.3063  (−0.2088) | 0.0362  (−0.3434) | −0.2126  (−0.2717) |
| O_3_ | 0.2579  (0.2994) | 0.2369  (0.2786) | 0.2367  (0.2765) | 0.2643  (0.3069) | 0.2706  (0.2869) |
| C_4_ | −0.2006  (−0.3603) | −0.2041  (−0.3026) | −0.0896  (−0.4347) | −0.1966  (−0.0999) | −0.3585  (−0.4345) |
| C_5_ | −0.8340  (−0.6686) | −0.2373  (−0.2720) | −0.4513  (−0.3786) | −0.7510  (−0.5303) | −0.4528  (−0.4406) |
| C_10_ | −0.7528  (−0.6048) | −0.3048  (−0.3291) | −0.5077  (−0.3413) | −0.6698  (−0.5741) | −0.5068  (−0.4830) |
| C_11_ | 0.6726  (0.4212) | −0.3841  (−0.3480) | −0.3053  (−0.5294) | 0.2349  (−0.0550) | −0.2209  (−0.2546) |
|  | N-protonation | | | | |
| N_1_ | 0.0680  (−0.2503) | 0.0757  (−0.2442) | 0.0744  (−0.2099) | 0.0736  (−0.2487) | 0.0569  (−0.1847) |
| C_2_ | −0.0572  (0.0041) | −0.1665  (−0.1429) | −0.1589  (−0.1327) | −0.0638  (−0.1500) | −0.1919  (−0.1801) |
| O_3_ | 0.0191  (0.2640) | −0.0034  (0.2448) | 0.0107  (0.2436) | 0.0114  (0.2618) | 0.0128  (0.2628) |
| C_4_ | 0.5379  (−0.1271) | 0.3288  (−0.2737) | 0.3513  (−0.2612) | 0.6475  (0.0365) | 0.3809  (−0.2961) |
| C_5_ | −0.8123  (−0.9076) | −0.3726  (−0.5349) | −0.4574  (−0.5557) | −1.0191  (−1.0046) | −0.6918  (−0.6949) |
| C_10_ | −1.2838  (−0.8544) | −0.7128  (−0.4835) | −0.8704  (−0.5158) | −1.3697  (−0.8824) | −1.1089  (−0.6090) |
| C_11_ | 0.7926  (0.4937) | −0.1998  (−0.3139) | −0.1921  (−0.4974) | 1.1198  (0.6583) | 0.3575  (−0.1385) |

**Table S3.** Electrostatic potentials at the nuclei of atoms for the TEMPO derivatives were optimized at the B3LYP/6-311++G(d,p) level of theory.

| TEMPO | |  | TEMPO-CH_3_ | |  | TEMPO-NH_2_ | |  | TEMPO-CHO | |  | TEMPO-NO_2_ | |
| --- | --- | --- | --- | --- | --- | --- | --- | --- | --- | --- | --- | --- | --- |
| N1 | -18.3211 |  | N1 | -18.3210 |  | N1 | -18.3194 |  | N1 | -18.3091 |  | N1 | -18.3010 |
| C2 | -14.7205 |  | C2 | -14.7204 |  | C2 | -14.7179 |  | C2 | -14.7067 |  | C2 | -14.6968 |
| O3 | -22.3915 |  | O3 | -22.3914 |  | O3 | -22.3899 |  | O3 | -22.3802 |  | O3 | -22.3725 |
| C4 | -14.7205 |  | C4 | -14.7204 |  | C4 | -14.7179 |  | C4 | -14.7067 |  | C4 | -14.6968 |
| C5 | -14.7692 |  | C5 | -14.7712 |  | C5 | -14.7709 |  | C5 | -14.7495 |  | C5 | -14.7402 |
| C6 | -14.7828 |  | C6 | -14.7828 |  | C6 | -14.7811 |  | C6 | -14.7733 |  | C6 | -14.7652 |
| C7 | -14.7886 |  | C7 | -14.7886 |  | C7 | -14.7876 |  | C7 | -14.7783 |  | C7 | -14.7721 |
| C8 | -14.7886 |  | C8 | -14.7886 |  | C8 | -14.7876 |  | C8 | -14.7783 |  | C8 | -14.7721 |
| C9 | -14.7828 |  | C9 | -14.7828 |  | C9 | -14.7811 |  | C9 | -14.7733 |  | C9 | -14.7652 |
| C10 | -14.7692 |  | C10 | -14.7712 |  | C10 | -14.7709 |  | C10 | -14.7495 |  | C10 | -14.7402 |
| C11 | -14.7678 |  | C11 | -14.7599 |  | C11 | -14.7333 |  | C11 | -14.7431 |  | C11 | -14.6921 |
| H12 | -1.1167 |  | H12 | -1.1168 |  | H12 | -1.1132 |  | H12 | -1.0994 |  | H12 | -1.0873 |
| H13 | -1.1209 |  | H13 | -1.1205 |  | H13 | -1.1214 |  | H13 | -1.1009 |  | H13 | -1.0934 |
| H14 | -1.1183 |  | H14 | -1.1183 |  | H14 | -1.1165 |  | H14 | -1.1091 |  | H14 | -1.1014 |
| H15 | -1.1201 |  | H15 | -1.1201 |  | H15 | -1.1183 |  | H15 | -1.1107 |  | H15 | -1.1019 |
| H16 | -1.1243 |  | H16 | -1.1243 |  | H16 | -1.1228 |  | H16 | -1.1147 |  | H16 | -1.1070 |
| H17 | -1.1223 |  | H17 | -1.1223 |  | H17 | -1.1214 |  | H17 | -1.1123 |  | H17 | -1.1063 |
| H18 | -1.1317 |  | H18 | -1.1316 |  | H18 | -1.1307 |  | H18 | -1.1215 |  | H18 | -1.1153 |
| H19 | -1.1269 |  | H19 | -1.1269 |  | H19 | -1.1262 |  | H19 | -1.1167 |  | H19 | -1.1111 |
| H20 | -1.1223 |  | H20 | -1.1223 |  | H20 | -1.1214 |  | H20 | -1.1123 |  | H20 | -1.1063 |
| H21 | -1.1269 |  | H21 | -1.1269 |  | H21 | -1.1262 |  | H21 | -1.1167 |  | H21 | -1.1111 |
| H22 | -1.1317 |  | H22 | -1.1316 |  | H22 | -1.1307 |  | H22 | -1.1215 |  | H22 | -1.1153 |
| H23 | -1.1183 |  | H23 | -1.1183 |  | H23 | -1.1165 |  | H23 | -1.1091 |  | H23 | -1.1014 |
| H24 | -1.1243 |  | H24 | -1.1243 |  | H24 | -1.1228 |  | H24 | -1.1147 |  | H24 | -1.1070 |
| H25 | -1.1201 |  | H25 | -1.1201 |  | H25 | -1.1183 |  | H25 | -1.1107 |  | H25 | -1.1019 |
| H26 | -1.1167 |  | H26 | -1.1168 |  | H26 | -1.1132 |  | H26 | -1.0994 |  | H26 | -1.0873 |
| H27 | -1.1209 |  | H27 | -1.1205 |  | H27 | -1.1214 |  | H27 | -1.1009 |  | H27 | -1.0934 |
| H28 | -1.1198 |  | H28 | -1.1235 |  | H28 | -1.1246 |  | H28 | -1.1000 |  | H28 | -1.0740 |
| H29 | -1.1189 |  | C29 | -14.7782 |  | N29 | -18.4092 |  | C29 | -14.6805 |  | N29 | -18.1555 |
|  |  |  | H30 | -1.1189 |  | H30 | -1.0573 |  | H30 | -1.0842 |  | O30 | -22.3286 |
|  |  |  | H31 | -1.1189 |  | H31 | -1.0573 |  | O31 | -22.3618 |  | O31 | -22.3288 |
|  |  |  | H32 | -1.1191 |  |  |  |  |  |  |  |  |  |

**Table S4.** Electrostatic potentials at the nuclei of atoms for the protonation of the oxygen atom of the NO moiety (**O-protonation**) for the TEMPO derivatives were optimized at the B3LYP/6-311++G(d,p) level of theory.

| TEMPO | |  | TEMPO-CH_3_ | |  | TEMPO-NH_2_ | |  | TEMPO-CHO | |  | TEMPO-NO_2_ | |
| --- | --- | --- | --- | --- | --- | --- | --- | --- | --- | --- | --- | --- | --- |
| N1 | -18.0877 |  | N1 | -18.0892 |  | N1 | -18.0873 |  | N1 | -18.0873 |  | N1 | -18.0704 |
| C2 | -14.5306 |  | C2 | -14.5456 |  | C2 | -14.5428 |  | C2 | -14.5428 |  | C2 | -14.5233 |
| O3 | -22.0942 |  | O3 | -22.0957 |  | O3 | -22.0939 |  | O3 | -22.0939 |  | O3 | -22.0778 |
| C4 | -14.5438 |  | C4 | -14.5325 |  | C4 | -14.5300 |  | C4 | -14.5300 |  | C4 | -14.5110 |
| C5 | -14.6150 |  | C5 | -14.6266 |  | C5 | -14.6259 |  | C5 | -14.6259 |  | C5 | -14.5968 |
| C6 | -14.6168 |  | C6 | -14.6301 |  | C6 | -14.6283 |  | C6 | -14.6283 |  | C6 | -14.6137 |
| C7 | -14.6076 |  | C7 | -14.6369 |  | C7 | -14.6358 |  | C7 | -14.6358 |  | C7 | -14.6214 |
| C8 | -14.6356 |  | C8 | -14.6091 |  | C8 | -14.6082 |  | C8 | -14.6082 |  | C8 | -14.5945 |
| C9 | -14.6286 |  | C9 | -14.6182 |  | C9 | -14.6164 |  | C9 | -14.6164 |  | C9 | -14.6017 |
| C10 | -14.6217 |  | C10 | -14.6200 |  | C10 | -14.6200 |  | C10 | -14.6200 |  | C10 | -14.5941 |
| C11 | -14.6269 |  | C11 | -14.6226 |  | C11 | -14.5946 |  | C11 | -14.5946 |  | C11 | -14.5555 |
| H12 | -0.9653 |  | H12 | -0.9745 |  | H12 | -0.97058 |  | H12 | -0.9706 |  | H12 | -0.9492 |
| H13 | -0.9695 |  | H13 | -0.9785 |  | H13 | -0.97893 |  | H13 | -0.9789 |  | H13 | -0.9500 |
| H14 | -0.9559 |  | H14 | -0.9690 |  | H14 | -0.96713 |  | H14 | -0.9671 |  | H14 | -0.9534 |
| H15 | -0.9601 |  | H15 | -0.9718 |  | H15 | -0.9699 |  | H15 | -0.9699 |  | H15 | -0.9547 |
| H16 | -0.9568 |  | H16 | -0.9728 |  | H16 | -0.97118 |  | H16 | -0.9712 |  | H16 | -0.9566 |
| H17 | -0.9454 |  | H17 | -0.9743 |  | H17 | -0.97316 |  | H17 | -0.9732 |  | H17 | -0.9591 |
| H18 | -0.9404 |  | H18 | -0.9808 |  | H18 | -0.97966 |  | H18 | -0.9797 |  | H18 | -0.9655 |
| H19 | -0.9495 |  | H19 | -0.9771 |  | H19 | -0.97625 |  | H19 | -0.9763 |  | H19 | -0.9619 |
| H20 | -0.9729 |  | H20 | -0.9471 |  | H20 | -0.9463 |  | H20 | -0.9463 |  | H20 | -0.9328 |
| H21 | -0.9757 |  | H21 | -0.9512 |  | H21 | -0.95068 |  | H21 | -0.9507 |  | H21 | -0.9369 |
| H22 | -0.9795 |  | H22 | -0.9420 |  | H22 | -0.94113 |  | H22 | -0.9411 |  | H22 | -0.9276 |
| H23 | -0.9676 |  | H23 | -0.9573 |  | H23 | -0.95546 |  | H23 | -0.9555 |  | H23 | -0.9415 |
| H24 | -0.9712 |  | H24 | -0.9583 |  | H24 | -0.95662 |  | H24 | -0.9566 |  | H24 | -0.9423 |
| H25 | -0.9702 |  | H25 | -0.9616 |  | H25 | -0.95974 |  | H25 | -0.9597 |  | H25 | -0.9443 |
| H26 | -0.9718 |  | H26 | -0.9682 |  | H26 | -0.96476 |  | H26 | -0.9648 |  | H26 | -0.9450 |
| H27 | -0.9763 |  | H27 | -0.9719 |  | H27 | -0.97292 |  | H27 | -0.9729 |  | H27 | -0.9483 |
| H28 | -0.9804 |  | H28 | -0.9883 |  | H28 | -0.98937 |  | H28 | -0.9894 |  | H28 | -0.9324 |
| H29 | -0.9809 |  | C29 | -14.6628 |  | N29 | -18.2904 |  | C29 | -18.2904 |  | N29 | -18.0361 |
| H30 | -0.7533 |  | H30 | -1.0053 |  | H30 | -0.93920 |  | H30 | -0.9392 |  | O30 | -22.2134 |
|  |  |  | H31 | -1.0051 |  | H31 | -0.93916 |  | O31 | -0.9392 |  | O31 | -22.2161 |
|  |  |  | H32 | -1.0046 |  | H32 | -0.7531 |  | H32 | -0.7531 |  | H32 | -0.7376 |
|  |  |  | H33 | -0.7548 |  |  |  |  |  |  |  |  |  |

**Table S5.** Electrostatic potentials at the nuclei of atoms for the protonation of the nitrogen atom of the NO moiety (**N-protonation**) for the TEMPO derivatives were optimized at the B3LYP/6-311++G(d,p) level of theory.

| TEMPO | |  | TEMPO-CH_3_ | |  | TEMPO-NH_2_ | |  | TEMPO-CHO | |  | TEMPO-NO_2_ | |
| --- | --- | --- | --- | --- | --- | --- | --- | --- | --- | --- | --- | --- | --- |
| N1 | -18.1170 |  | N1 | -18.1168 |  | N1 | -18.1157 |  | N1 | -18.1101 |  | N1 | -18.1062 |
| C2 | -14.5647 |  | C2 | -14.5653 |  | C2 | -14.5624 |  | C2 | -14.5587 |  | C2 | -14.5473 |
| O3 | -22.1845 |  | O3 | -22.1844 |  | O3 | -22.1834 |  | O3 | -22.1779 |  | O3 | -22.1737 |
| C4 | -14.5130 |  | C4 | -14.5153 |  | C4 | -14.5138 |  | C4 | -14.5062 |  | C4 | -14.4964 |
| C5 | -14.6187 |  | C5 | -14.6230 |  | C5 | -14.6210 |  | C5 | -14.6151 |  | C5 | -14.5986 |
| C6 | -14.6450 |  | C6 | -14.6458 |  | C6 | -14.6438 |  | C6 | -14.6398 |  | C6 | -14.6318 |
| C7 | -14.6421 |  | C7 | -14.6426 |  | C7 | -14.6413 |  | C7 | -14.6385 |  | C7 | -14.6305 |
| C8 | -14.5998 |  | C8 | -14.6023 |  | C8 | -14.6019 |  | C8 | -14.5937 |  | C8 | -14.5877 |
| C9 | -14.6046 |  | C9 | -14.6069 |  | C9 | -14.6054 |  | C9 | -14.5979 |  | C9 | -14.5907 |
| C10 | -14.5901 |  | C10 | -14.5957 |  | C10 | -14.5966 |  | C10 | -14.5768 |  | C10 | -14.5714 |
| C11 | -14.6100 |  | C11 | -14.6055 |  | C11 | -14.5765 |  | C11 | -14.5887 |  | C11 | -14.5395 |
| H12 | -0.9691 |  | H12 | -0.9714 |  | H12 | -0.9660 |  | H12 | -0.9666 |  | H12 | -0.9495 |
| H13 | -0.9688 |  | H13 | -0.9706 |  | H13 | -0.9699 |  | H13 | -0.9633 |  | H13 | -0.9469 |
| H14 | -0.9815 |  | H14 | -0.9822 |  | H14 | -0.9801 |  | H14 | -0.9768 |  | H14 | -0.9692 |
| H15 | -0.9821 |  | H15 | -0.9830 |  | H15 | -0.9810 |  | H15 | -0.9763 |  | H15 | -0.9680 |
| H16 | -0.9882 |  | H16 | -0.9890 |  | H16 | -0.9873 |  | H16 | -0.9834 |  | H16 | -0.9757 |
| H17 | -0.9790 |  | H17 | -0.9796 |  | H17 | -0.9782 |  | H17 | -0.9758 |  | H17 | -0.9681 |
| H18 | -0.9847 |  | H18 | -0.9852 |  | H18 | -0.9839 |  | H18 | -0.9812 |  | H18 | -0.9734 |
| H19 | -0.9801 |  | H19 | -0.9806 |  | H19 | -0.9795 |  | H19 | -0.9767 |  | H19 | -0.9688 |
| H20 | -0.9119 |  | H20 | -0.9157 |  | H20 | -0.9151 |  | H20 | -0.9062 |  | H20 | -0.8991 |
| H21 | -0.9358 |  | H21 | -0.9384 |  | H21 | -0.9382 |  | H21 | -0.9298 |  | H21 | -0.9241 |
| H22 | -0.9381 |  | H22 | -0.9409 |  | H22 | -0.9404 |  | H22 | -0.9321 |  | H22 | -0.9251 |
| H23 | -0.9200 |  | H23 | -0.9225 |  | H23 | -0.9203 |  | H23 | -0.9142 |  | H23 | -0.9084 |
| H24 | -0.9321 |  | H24 | -0.9363 |  | H24 | -0.9358 |  | H24 | -0.9251 |  | H24 | -0.9151 |
| H25 | -0.9456 |  | H25 | -0.9478 |  | H25 | -0.9460 |  | H25 | -0.9383 |  | H25 | -0.9310 |
| H26 | -0.9160 |  | H26 | -0.9195 |  | H26 | -0.9173 |  | H26 | -0.9048 |  | H26 | -0.8945 |
| H27 | -0.9453 |  | H27 | -0.9482 |  | H27 | -0.9504 |  | H27 | -0.9336 |  | H27 | -0.9236 |
| H28 | -0.9671 |  | H28 | -0.9757 |  | H28 | -0.9768 |  | H28 | -0.9386 |  | H28 | -0.9169 |
| H29 | -0.9695 |  | H29 | -0.8416 |  | H29 | -0.8405 |  | H29 | -0.8352 |  | H29 | -0.8298 |
| H30 | -0.8414 |  | C30 | -14.6521 |  | N30 | -18.2784 |  | C30 | -14.5501 |  | N30 | -18.0227 |
|  |  |  | H31 | -0.9949 |  | H31 | -0.9270 |  | H31 | -0.9531 |  | O31 | -22.2006 |
|  |  |  | H32 | -0.9940 |  | H32 | -0.9269 |  | O32 | -22.2339 |  | O32 | -22.1995 |
|  |  |  | H33 | -0.9937 |  |  |  |  |  |  |  |  |  |

**Table S6.** Electrostatic potentials at the nuclei of atoms for the TEMPO derivatives were optimized at the M062X/6-311++G(d,p) level of theory.

| TEMPO | |  | TEMPO-CH_3_ | |  | TEMPO-NH_2_ | |  | TEMPO-CHO | |  | TEMPO-NO_2_ | |
| --- | --- | --- | --- | --- | --- | --- | --- | --- | --- | --- | --- | --- | --- |
| N1 | -18.3115 |  | N1 | -18.3114 |  | N1 | -18.3098 |  | N1 | -18.2996 |  | N1 | -18.2906 |
| C2 | -14.7169 |  | C2 | -14.7168 |  | C2 | -14.7142 |  | C2 | -14.7031 |  | C2 | -14.6923 |
| O3 | -22.3810 |  | O3 | -22.3809 |  | O3 | -22.3794 |  | O3 | -22.3697 |  | O3 | -22.3615 |
| C4 | -14.7169 |  | C4 | -14.7168 |  | C4 | -14.7142 |  | C4 | -14.7031 |  | C4 | -14.6923 |
| C5 | -14.7641 |  | C5 | -14.7659 |  | C5 | -14.7658 |  | C5 | -14.7442 |  | C5 | -14.7337 |
| C6 | -14.7776 |  | C6 | -14.7776 |  | C6 | -14.7759 |  | C6 | -14.7680 |  | C6 | -14.7591 |
| C7 | -14.7839 |  | C7 | -14.7838 |  | C7 | -14.7829 |  | C7 | -14.7735 |  | C7 | -14.7668 |
| C8 | -14.7839 |  | C8 | -14.7838 |  | C8 | -14.7829 |  | C8 | -14.7735 |  | C8 | -14.7668 |
| C9 | -14.7776 |  | C9 | -14.7776 |  | C9 | -14.7759 |  | C9 | -14.7680 |  | C9 | -14.7591 |
| C10 | -14.7641 |  | C10 | -14.7659 |  | C10 | -14.7658 |  | C10 | -14.7442 |  | C10 | -14.7337 |
| C11 | -14.7626 |  | C11 | -14.7554 |  | C11 | -14.7280 |  | C11 | -14.7388 |  | C11 | -14.6848 |
| H12 | -1.1127 |  | H12 | -1.1125 |  | H12 | -1.1090 |  | H12 | -1.0950 |  | H12 | -1.0817 |
| H13 | -1.1167 |  | H13 | -1.1160 |  | H13 | -1.1171 |  | H13 | -1.0960 |  | H13 | -1.0879 |
| H14 | -1.1144 |  | H14 | -1.1144 |  | H14 | -1.1125 |  | H14 | -1.1052 |  | H14 | -1.0967 |
| H15 | -1.1159 |  | H15 | -1.1159 |  | H15 | -1.1141 |  | H15 | -1.1065 |  | H15 | -1.0969 |
| H16 | -1.1197 |  | H16 | -1.1198 |  | H16 | -1.1184 |  | H16 | -1.1102 |  | H16 | -1.1018 |
| H17 | -1.1190 |  | H17 | -1.1190 |  | H17 | -1.1181 |  | H17 | -1.1090 |  | H17 | -1.1024 |
| H18 | -1.1284 |  | H18 | -1.1283 |  | H18 | -1.1273 |  | H18 | -1.1182 |  | H18 | -1.1115 |
| H19 | -1.1236 |  | H19 | -1.1236 |  | H19 | -1.1230 |  | H19 | -1.1134 |  | H19 | -1.1073 |
| H20 | -1.1190 |  | H20 | -1.1190 |  | H20 | -1.1181 |  | H20 | -1.1090 |  | H20 | -1.1024 |
| H21 | -1.1236 |  | H21 | -1.1236 |  | H21 | -1.1230 |  | H21 | -1.1134 |  | H21 | -1.1073 |
| H22 | -1.1284 |  | H22 | -1.1283 |  | H22 | -1.1273 |  | H22 | -1.1182 |  | H22 | -1.1115 |
| H23 | -1.1144 |  | H23 | -1.1144 |  | H23 | -1.1125 |  | H23 | -1.1052 |  | H23 | -1.0967 |
| H24 | -1.1197 |  | H24 | -1.1198 |  | H24 | -1.1184 |  | H24 | -1.1102 |  | H24 | -1.1018 |
| H25 | -1.1159 |  | H25 | -1.1159 |  | H25 | -1.1141 |  | H25 | -1.1065 |  | H25 | -1.0969 |
| H26 | -1.1127 |  | H26 | -1.1125 |  | H26 | -1.1090 |  | H26 | -1.0950 |  | H26 | -1.0817 |
| H27 | -1.1167 |  | H27 | -1.1160 |  | H27 | -1.1171 |  | H27 | -1.0960 |  | H27 | -1.0879 |
| H28 | -1.1154 |  | H28 | -1.1190 |  | H28 | -1.1205 |  | H28 | -1.0947 |  | H28 | -1.0672 |
| H29 | -1.1153 |  | C29 | -14.7736 |  | N29 | -18.4031 |  | C29 | -14.6748 |  | N29 | -18.1356 |
|  |  |  | H30 | -1.1158 |  | H30 | -1.0538 |  | H30 | -1.0806 |  | O30 | -22.3188 |
|  |  |  | H31 | -1.1158 |  | H31 | -1.0538 |  | O31 | -22.3518 |  | O31 | -22.3197 |
|  |  |  | H32 | -1.1157 |  |  |  |  |  |  |  |  |  |

**Table S7.** Electrostatic potentials at the nuclei of atoms for the protonation of the oxygen atom of the NO moiety (**O-protonation**) for the TEMPO derivatives were optimized at the M062X/6-311++G(d,p) level of theory.

| TEMPO | |  | TEMPO-CH_3_ | |  | TEMPO-NH_2_ | |  | TEMPO-CHO | |  | TEMPO-NO_2_ | |
| --- | --- | --- | --- | --- | --- | --- | --- | --- | --- | --- | --- | --- | --- |
| N1 | -18.0744 |  | N1 | -18.0761 |  | N1 | -18.0742 |  | N1 | -18.0649 |  | N1 | -18.0565 |
| C2 | -14.5260 |  | C2 | -14.5416 |  | C2 | -14.5388 |  | C2 | -14.5151 |  | C2 | -14.5184 |
| O3 | -22.0797 |  | O3 | -22.0812 |  | O3 | -22.0794 |  | O3 | -22.0707 |  | O3 | -22.0626 |
| C4 | -14.5398 |  | C4 | -14.5279 |  | C4 | -14.5253 |  | C4 | -14.5286 |  | C4 | -14.5054 |
| C5 | -14.6088 |  | C5 | -14.6204 |  | C5 | -14.6199 |  | C5 | -14.5932 |  | C5 | -14.5898 |
| C6 | -14.6104 |  | C6 | -14.6240 |  | C6 | -14.6221 |  | C6 | -14.6033 |  | C6 | -14.6067 |
| C7 | -14.6018 |  | C7 | -14.6313 |  | C7 | -14.6301 |  | C7 | -14.5940 |  | C7 | -14.6152 |
| C8 | -14.6300 |  | C8 | -14.6034 |  | C8 | -14.6024 |  | C8 | -14.6218 |  | C8 | -14.5880 |
| C9 | -14.6225 |  | C9 | -14.6119 |  | C9 | -14.6099 |  | C9 | -14.6152 |  | C9 | -14.5945 |
| C10 | -14.6157 |  | C10 | -14.6137 |  | C10 | -14.6139 |  | C10 | -14.6002 |  | C10 | -14.5864 |
| C11 | -14.6209 |  | C11 | -14.6173 |  | C11 | -14.5885 |  | C11 | -14.6025 |  | C11 | -14.5468 |
| H12 | -0.9606 |  | H12 | -0.9695 |  | H12 | -0.9657 |  | H12 | -0.9469 |  | H12 | -0.9434 |
| H13 | -0.9644 |  | H13 | -0.9732 |  | H13 | -0.9738 |  | H13 | -0.9478 |  | H13 | -0.9436 |
| H14 | -0.9512 |  | H14 | -0.9645 |  | H14 | -0.9625 |  | H14 | -0.9444 |  | H14 | -0.9481 |
| H15 | -0.9552 |  | H15 | -0.9670 |  | H15 | -0.9649 |  | H15 | -0.9483 |  | H15 | -0.9489 |
| H16 | -0.9512 |  | H16 | -0.9674 |  | H16 | -0.9657 |  | H16 | -0.9442 |  | H16 | -0.9503 |
| H17 | -0.9410 |  | H17 | -0.9702 |  | H17 | -0.9691 |  | H17 | -0.9333 |  | H17 | -0.9545 |
| H18 | -0.9368 |  | H18 | -0.9767 |  | H18 | -0.9755 |  | H18 | -0.9292 |  | H18 | -0.9607 |
| H19 | -0.9438 |  | H19 | -0.9726 |  | H19 | -0.9718 |  | H19 | -0.9361 |  | H19 | -0.9569 |
| H20 | -0.9689 |  | H20 | -0.9427 |  | H20 | -0.9418 |  | H20 | -0.9608 |  | H20 | -0.9276 |
| H21 | -0.9712 |  | H21 | -0.9455 |  | H21 | -0.9450 |  | H21 | -0.9631 |  | H21 | -0.9304 |
| H22 | -0.9753 |  | H22 | -0.9384 |  | H22 | -0.9374 |  | H22 | -0.9673 |  | H22 | -0.9232 |
| H23 | -0.9631 |  | H23 | -0.9526 |  | H23 | -0.9507 |  | H23 | -0.9559 |  | H23 | -0.9360 |
| H24 | -0.9657 |  | H24 | -0.9527 |  | H24 | -0.9510 |  | H24 | -0.9585 |  | H24 | -0.9360 |
| H25 | -0.9654 |  | H25 | -0.9567 |  | H25 | -0.9547 |  | H25 | -0.9582 |  | H25 | -0.9385 |
| H26 | -0.9672 |  | H26 | -0.9631 |  | H26 | -0.9598 |  | H26 | -0.9528 |  | H26 | -0.9389 |
| H27 | -0.9712 |  | H27 | -0.9665 |  | H27 | -0.9677 |  | H27 | -0.9543 |  | H27 | -0.9415 |
| H28 | -0.9751 |  | H28 | -0.9830 |  | H28 | -0.9844 |  | H28 | -0.9604 |  | H28 | -0.9240 |
| H29 | -0.9767 |  | C29 | -14.6574 |  | N29 | -18.2838 |  | C29 | -14.5578 |  | N29 | -18.0154 |
| H30 | -0.7427 |  | H30 | -1.0015 |  | H30 | -0.9350 |  | H30 | -0.9629 |  | O30 | -22.2033 |
|  |  |  | H31 | -1.0013 |  | H31 | -0.9350 |  | O31 | -22.2381 |  | O31 | -22.2064 |
|  |  |  | H32 | -1.0004 |  | H32 | -0.7425 |  | H32 | -0.7340 |  | H32 | -0.7262 |
|  |  |  | H33 | -0.7442 |  |  |  |  |  |  |  |  |  |

**Table S8.** Electrostatic potentials at the nuclei of atoms for the protonation of the nitrogen atom of the NO moiety (**N-protonation**) for the TEMPO derivatives were optimized at the M062X/6-311++G(d,p) level of theory.

| TEMPO | |  | TEMPO-CH_3_ | |  | TEMPO-NH_2_ | |  | TEMPO-CHO | |  | TEMPO-NO_2_ | |
| --- | --- | --- | --- | --- | --- | --- | --- | --- | --- | --- | --- | --- | --- |
| N1 | -18.0570 |  | N1 | -18.0588 |  | N1 | -18.0572 |  | N1 | -18.0507 |  | N1 | -18.0393 |
| C2 | -14.5326 |  | C2 | -14.5343 |  | C2 | -14.5319 |  | C2 | -14.5136 |  | C2 | -14.5120 |
| O3 | -22.0984 |  | O3 | -22.1000 |  | O3 | -22.0984 |  | O3 | -22.0928 |  | O3 | -22.0819 |
| C4 | -14.5198 |  | C4 | -14.5219 |  | C4 | -14.5192 |  | C4 | -14.5235 |  | C4 | -14.4993 |
| C5 | -14.6048 |  | C5 | -14.6097 |  | C5 | -14.6094 |  | C5 | -14.5971 |  | C5 | -14.5797 |
| C6 | -14.6218 |  | C6 | -14.6233 |  | C6 | -14.6214 |  | C6 | -14.6100 |  | C6 | -14.6058 |
| C7 | -14.6168 |  | C7 | -14.6182 |  | C7 | -14.6172 |  | C7 | -14.6102 |  | C7 | -14.6024 |
| C8 | -14.6132 |  | C8 | -14.6148 |  | C8 | -14.6138 |  | C8 | -14.6101 |  | C8 | -14.5995 |
| C9 | -14.6154 |  | C9 | -14.6171 |  | C9 | -14.6151 |  | C9 | -14.6141 |  | C9 | -14.5991 |
| C10 | -14.6003 |  | C10 | -14.6054 |  | C10 | -14.6055 |  | C10 | -14.5893 |  | C10 | -14.5783 |
| C11 | -14.6125 |  | C11 | -14.6091 |  | C11 | -14.5803 |  | C11 | -14.5930 |  | C11 | -14.5389 |
| H12 | -0.9537 |  | H12 | -0.9560 |  | H12 | -0.9524 |  | H12 | -0.9438 |  | H12 | -0.9309 |
| H13 | -0.9564 |  | H13 | -0.9585 |  | H13 | -0.9592 |  | H13 | -0.9499 |  | H13 | -0.9296 |
| H14 | -0.9599 |  | H14 | -0.9613 |  | H14 | -0.9594 |  | H14 | -0.9471 |  | H14 | -0.9450 |
| H15 | -0.9636 |  | H15 | -0.9653 |  | H15 | -0.9632 |  | H15 | -0.9520 |  | H15 | -0.9469 |
| H16 | -0.9611 |  | H16 | -0.9627 |  | H16 | -0.9610 |  | H16 | -0.9497 |  | H16 | -0.9458 |
| H17 | -0.9555 |  | H17 | -0.9570 |  | H17 | -0.9560 |  | H17 | -0.9474 |  | H17 | -0.9417 |
| H18 | -0.9580 |  | H18 | -0.9595 |  | H18 | -0.9585 |  | H18 | -0.9544 |  | H18 | -0.9439 |
| H19 | -0.9545 |  | H19 | -0.9559 |  | H19 | -0.9552 |  | H19 | -0.9472 |  | H19 | -0.9404 |
| H20 | -0.9505 |  | H20 | -0.9522 |  | H20 | -0.9512 |  | H20 | -0.9489 |  | H20 | -0.9371 |
| H21 | -0.9502 |  | H21 | -0.9519 |  | H21 | -0.9511 |  | H21 | -0.9480 |  | H21 | -0.9368 |
| H22 | -0.9573 |  | H22 | -0.9588 |  | H22 | -0.9578 |  | H22 | -0.9515 |  | H22 | -0.9439 |
| H23 | -0.9522 |  | H23 | -0.9540 |  | H23 | -0.9519 |  | H23 | -0.9525 |  | H23 | -0.9367 |
| H24 | -0.9550 |  | H24 | -0.9568 |  | H24 | -0.9549 |  | H24 | -0.9538 |  | H24 | -0.9394 |
| H25 | -0.9579 |  | H25 | -0.9598 |  | H25 | -0.9576 |  | H25 | -0.9554 |  | H25 | -0.9409 |
| H26 | -0.9475 |  | H26 | -0.9501 |  | H26 | -0.9468 |  | H26 | -0.9381 |  | H26 | -0.9264 |
| H27 | -0.9531 |  | H27 | -0.9553 |  | H27 | -0.9563 |  | H27 | -0.9426 |  | H27 | -0.9305 |
| H28 | -0.9683 |  | H28 | -0.9764 |  | H28 | -0.9778 |  | H28 | -0.9420 |  | H28 | -0.9172 |
| H29 | -0.9694 |  | H29 | -0.7979 |  | H29 | -0.7960 |  | H29 | -0.7876 |  | H29 | -0.7770 |
| H30 | -0.7957 |  | C30 | -14.6511 |  | N30 | -18.2774 |  | C30 | -14.5500 |  | N30 | -18.0090 |
|  |  |  | H31 |  |  | H31 | -0.9285 |  | H31 | -0.9569 |  | O31 | -22.2002 |
|  |  |  | H32 |  |  | H32 | -0.9286 |  | O32 | -22.2298 |  | O32 | -22.1971 |
|  |  |  | H33 |  |  |  |  |  |  |  |  |  |  |

**Table S9.** Summary of important second-order delocalization energies (*E*_2_) for the orbital interactions of TEMPO derivatives, determined using NBO analysis at the B3LYP/6-311++G(d,p) level of theory.

| Donor  NBO(*i*) | ED(*i*) (a.u.), X=H | Acceptor  NBO(*j*) | ED(*j*) (a.u.), X=H | Interaction type | *E*_2_ (kcal/mol) | | | | |
| --- | --- | --- | --- | --- | --- | --- | --- | --- | --- |
|  |  |  |  |  | −H | −CH_3_ | −NH_2_ | −CHO | −NO_2_ |
| *σ*_N1−C2_ | 0.99034 | *σ*^*^_N1−C4_ | 0.03911 | *σ* _N1−C2_ → *σ*^*^_N1−C4_ | 0.26 | 0.26 | 0.26 | 0.27 | 0.27 |
|  |  | *σ*^*^_C4−C8_ | 0.00885 | *σ* _N1−C2_ → *σ*^*^_C4−C8_ | 0.34 | 0.35 | 0.40 | 0.35 | 0.35 |
|  |  | *σ*^*^_C5−H12_ | 0.00616 | *σ* _N1−C2_ → *σ*^*^_C5−H12_ | 0.66 | 0.68 | 0.63 | 0.66 | 0.61 |
|  |  | *σ*^*^_C6−H14_ | 0.00296 | *σ* _N1−C2_ → *σ*^*^_C6−H14_ | 0.60 | 0.68 | 0.64 | 0.67 | 0.66 |
| *σ*_N1−O3_ | 0.99616 | *σ*^*^_N1−C2_ | 0.03911 | *σ*_N1−O3_ → *σ*^*^_N1−C2_ | 0.30 | 0.30 | 0.30 | 0.31 | 0.31 |
|  |  | *σ*^*^_C2−C5_ | 0.01529 | *σ*_N1−O3_ → *σ*^*^_C2−C5_ | 0.42 | 0.43 | 0.44 | 0.43 | 0.43 |
| π_N1−O3_ | 0.98799 | *σ*^*^_C2−C5_ | 0.01529 | π_N1−O3_ → *σ*^*^_C2−C5_ | 0.28 | 0.28 | 0.27 | 0.32 | 0.32 |
|  |  | *σ*^*^_C2−C6_ | 0.01155 | π_N1−O3_ → *σ*^*^_C2−C6_ | 1.20 | 1.21 | 1.19 | 1.24 | 1.23 |
|  |  | *σ*^*^_C2−C7_ | 0.00885 | π_N1−O3_ → *σ*^*^_C2−C7_ | 0.30 | 0.31 | 0.32 | 0.30 | 0.31 |
|  |  | *σ*^*^_C6−H14_ | 0.00296 | π_N1−O3_ → *σ*^*^_C6−H14_ | 0.27 | 0.27 | 0.27 | 0.26 | 0.26 |
| *σ*_C2−C5_ | 0.98525 | *σ*^*^_N1−O3_ | 0.00772 | *σ*_C2−C5_ → *σ*^*^_N1−O3_ | 1.47 | 1.47 | 1.55 | 1.44 | 1.42 |
|  |  | *σ*^*^_C2−C6_ | 0.01155 | *σ*_C2−C5_ → *σ*^*^_C2−C6_ | 0.40 | 0.42 | 0.38 | 0.39 | 0.37 |
|  |  | *σ*^*^_C5−C11_ | 0.00682 | *σ*_C2−C5_ → *σ*^*^_C5−C11_ | 0.48 | 0.55 | 0.48 | 0.57 | 0.56 |
|  |  | *σ*^*^_C6−H16_ | 0.00298 | *σ*_C2−C5_ → *σ*^*^_C6−H16_ | 0.69 | 0.68 | 0.66 | 0.67 | 0.66 |
| *σ*_C2−C6_ | 0.98018 | π^*^_N1−O3_ | 0.00772 | *σ*_C2−C6_ → π^*^_N1−O3_ | 1.65 | 1.67 | 1.65 | 1.66 | 1.62 |
|  |  | *σ*^*^_C2−C5_ | 0.01529 | *σ*_C2−C6_ → *σ*^*^_C2−C5_ | 0.43 | 0.46 | 0.42 | 0.45 | 0.41 |
|  |  | *σ*^*^_C2−C7_ | 0.00885 | *σ*_C2−C6_ → *σ*^*^_C2−C7_ | 0.41 | 0.41 | 0.36 | 0.43 | 0.45 |
|  |  | *σ*^*^_C5−H13_ | 0.00803 | *σ*_C2−C6_ → *σ*^*^_C5−H13_ | 0.90 | 0.95 | 0.93 | 0.95 | 0.92 |
|  |  | *σ*^*^_C7−H19_ | 0.00283 | *σ*_C2−C6_ → *σ*^*^_C7−H19_ | 0.87 | 0.87 | 0.88 | 0.86 | 0.85 |
| *σ*_C2−C7_ | 0.98456 | π^*^_N1−O3_ | 0.00772 | *σ*_C2−C7_ → π^*^_N1−O3_ | 0.34 | 0.35 | 0.25 | 0.33 | 0.34 |
|  |  | *σ*^*^_N1−C4_ | 0.03911 | *σ*_C2−C7_ → *σ*^*^_N1−C4_ | 1.45 | 1.45 | 1.52 | 1.47 | 1.46 |
|  |  | *σ*^*^_C2−C5_ | 0.01529 | *σ*_C2−C7_ → *σ*^*^_C2−C5_ | 0.52 | 0.56 | 0.57 | 0.55 | 0.56 |
|  |  | *σ*^*^_C2−C6_ | 0.01155 | *σ*_C2−C7_ → *σ*^*^_C2−C6_ | 0.46 | 0.46 | 0.39 | 0.47 | 0.49 |
|  |  | *σ*^*^_C5−C11_ | 0.00682 | *σ*_C2−C7_ → *σ*^*^_C5−C11_ | 1.11 | 1.10 | 1.05 | 1.14 | 1.19 |
|  |  | *σ*^*^_C6−H15_ | 0.00346 | *σ*_C2−C7_ → *σ*^*^_C6−H15_ | 0.93 | 0.93 | 0.92 | 0.92 | 0.94 |
| *σ*_C5−C11_ | 0.99057 | *σ*^*^_C2−C5_ | 0.01529 | *σ*_C5−C11_ → *σ*^*^_C2−C5_ | 0.47 | 0.50 | 0.48 | 0.41 | 0.48 |
|  |  | *σ*^*^_C2−C7_ | 0.00885 | *σ*_C5−C11_ → *σ*^*^_C2−C7_ | 1.09 | 1.00 | 1.01 | 1.00 | 0.99 |
|  |  | *σ*^*^_C10−H26_ | 0.00616 | *σ*_C5−C11_ → *σ*^*^_C10−H26_ | 0.99 | 1.06 | 1.04 | 0.98 | 0.89 |
| *σ*_C5−H12_ | 0.98848 | *σ*^*^_N1−C2_ | 0.03911 | *σ*_C5−C12_ → *σ*^*^_N1−C2_ | 2.19 | 2.20 | 2.30 | 2.15 | 2.14 |
|  |  | *σ*^*^_C10−C11_ | 0.00682 | *σ*_C5−C12_ → *σ*^*^_C10−C11_ | 1.34 | 1.41 | 1.35 | 1.35 | 1.50 |
| *σ*_C6−H14_ | 0.99082 | *σ*^*^_N1−C2_ | 0.03911 | *σ*_C6−C14_ → *σ*^*^_N1−C2_ | 2.31 | 2.30 | 2.22 | 2.34 | 2.35 |
| *σ*_C6−H15_ | 0.99382 | *σ*^*^_N1−C2_ | 0.03911 | *σ*_C6−C15_ → *σ*^*^_N1−C2_ | 0.29 | 0.29 | 0.34 | 0.29 | 0.29 |
|  |  | *σ*^*^_C2−C5_ | 0.01529 | *σ*_C6−C15_→ *σ*^*^_C2−C5_ | 1.82 | 1.85 | 1.87 | 1.89 | 1.90 |
|  |  | *σ*^*^_C2−C7_ | 0.00885 | *σ*_C6−C15_ → *σ*^*^_C2−C7_ | 1.62 | 1.61 | 1.62 | 1.62 | 1.60 |
| *σ*_C11−H28_ | 0.98935 | *σ*^*^_C5−H13_ | 0.00803 | *σ*_C11−H28_ → *σ*^*^_C5−H13_ | 1.34 | 1.52 | 1.39 | 1.34 | 1.31 |
| LP(1)_O3_ | 0.99118 | *σ*^*^_N1−C2_ | 0.03911 | LP(1)_O3_ → *σ*^*^_N1−C2_ | 1.63 | 1.63 | 1.64 | 1.64 | 1.66 |
| LP(2)_O3_ | 0.96106 | *σ*^*^_N1−C2_ | 0.03911 | LP(2)_O3_ → *σ*^*^_N1−C2_ | 4.94 | 4.93 | 4.95 | 4.96 | 4.99 |
|  |  | *σ*^*^_C4−C10_ | 0.01529 | LP(2)_O3_ → *σ*^*^_C4−C10_ | 0.57 | 0.57 | 0.57 | 0.58 | 0.59 |

**Table S10.** Summary of important second-order delocalization energies (*E*_2_) for the orbital interactions of TEMPO derivatives, determined using NBO analysis at the M06-2X/6-311++G(d,p) level of theory.

| Donor  NBO(*i*) | ED(*i*) (a.u.), X=H | Acceptor  NBO(*j*) | ED(*j*) (a.u.), X=H | Interaction type | *E*_2_ (kcal/mol) | | | | |
| --- | --- | --- | --- | --- | --- | --- | --- | --- | --- |
|  |  |  |  |  | −H | −CH_3_ | −NH_2_ | −CHO | −NO_2_ |
| *σ*_N1−C2_ | 0.99027 | *σ*^*^_N1−C4_ | 0.03742 | *σ* _N1−C2_ → *σ*^*^_N1−C4_ | 0.35 | 0.35 | 0.35 | 0.36 | 0.35 |
|  |  | *σ*^*^_C4−C8_ | 0.00900 | *σ* _N1−C2_ → *σ*^*^_C4−C8_ | 0.41 | 0.41 | 0.40 | 0.42 | 0.41 |
|  |  | *σ*^*^_C5−H12_ | 0.00603 | *σ* _N1−C2_ → *σ*^*^_C5−H12_ | 0.75 | 0.79 | 0.80 | 0.77 | 0.76 |
|  |  | *σ*^*^_C6−H14_ | 0.00294 | *σ* _N1−C2_ → *σ*^*^_C6−H14_ | 0.65 | 0.65 | 0.65 | 0.63 | 0.63 |
| *σ*_N1−O3_ | 0.99613 | *σ*^*^_N1−C2_ | 0.03742 | *σ*_N1−O3_ → *σ*^*^_N1−C2_ | 0.38 | 0.38 | 0.38 | 0.38 | 0.39 |
|  |  | *σ*^*^_C2−C5_ | 0.01507 | *σ*_N1−O3_ → *σ*^*^_C2−C5_ | 0.46 | 0.48 | 0.47 | 0.48 | 0.47 |
| π_N1−O3_ | 0.98861 | *σ*^*^_C2−C5_ | 0.01507 | π_N1−O3_ → *σ*^*^_C2−C5_ | 0.41 | 0.40 | 0.39 | 0.44 | 0.45 |
|  |  | *σ*^*^_C2−C6_ | 0.01166 | π_N1−O3_ → *σ*^*^_C2−C6_ | 1.44 | 1.44 | 1.43 | 1.47 | 1.47 |
|  |  | *σ*^*^_C2−C7_ | 0.00900 | π_N1−O3_ → *σ*^*^_C2−C7_ | 0.32 | 0.32 | 0.34 | 0.31 | 0.33 |
|  |  | *σ*^*^_C6−H14_ | 0.00294 | π_N1−O3_ → *σ*^*^_C6−H14_ | 0.28 | 0.29 | 0.29 | 0.27 | 0.28 |
| *σ*_C2−C5_ | 0.98518 | *σ*^*^_N1−O3_ | 0.00748 | *σ*_C2−C5_ → *σ*^*^_N1−O3_ | 1.69 | 1.68 | 1.69 | 1.65 | 1.62 |
|  |  | *σ*^*^_C2−C6_ | 0.01166 | *σ*_C2−C5_ → *σ*^*^_C2−C6_ | 0.51 | 0.54 | 0.53 | 0.51 | 0.47 |
|  |  | *σ*^*^_C5−C11_ | 0.00691 | *σ*_C2−C5_ → *σ*^*^_C5−C11_ | 0.61 | 0.70 | 0.61 | 0.73 | 0.68 |
|  |  | *σ*^*^_C6−H16_ | 0.00287 | *σ*_C2−C5_ → *σ*^*^_C6−H16_ | 0.78 | 0.77 | 0.76 | 0.75 | 0.73 |
| *σ*_C2−C6_ | 0.98048 | π^*^_N1−O3_ | 0.02771 | *σ*_C2−C6_ → π^*^_N1−O3_ | 2.19 | 2.20 | 2.18 | 2.18 | 2.13 |
|  |  | *σ*^*^_C2−C5_ | 0.01507 | *σ*_C2−C6_ → *σ*^*^_C2−C5_ | 0.56 | 0.60 | 0.58 | 0.58 | 0.53 |
|  |  | *σ*^*^_C2−C7_ | 0.00900 | *σ*_C2−C6_ → *σ*^*^_C2−C7_ | 0.54 | 0.54 | 0.54 | 0.56 | 0.59 |
|  |  | *σ*^*^_C5−H13_ | 0.00781 | *σ*_C2−C6_ → *σ*^*^_C5−H13_ | 1.05 | 1.12 | 1.09 | 1.12 | 1.05 |
|  |  | *σ*^*^_C7−H19_ | 0.00276 | *σ*_C2−C6_ → *σ*^*^_C7−H19_ | 0.97 | 0.97 | 0.96 | 0.95 | 0.93 |
| *σ*_C2−C7_ | 0.98479 | π^*^_N1−O3_ | 0.02771 | *σ*_C2−C7_ → π^*^_N1−O3_ | 0.38 | 0.39 | 0.41 | 0.37 | 0.38 |
|  |  | *σ*^*^_N1−C4_ | 0.03742 | *σ*_C2−C7_ → *σ*^*^_N1−C4_ | 1.74 | 1.73 | 1.73 | 1.75 | 1.74 |
|  |  | *σ*^*^_C2−C5_ | 0.01507 | *σ*_C2−C7_ → *σ*^*^_C2−C5_ | 0.66 | 0.71 | 0.72 | 0.70 | 0.70 |
|  |  | *σ*^*^_C2−C6_ | 0.01166 | *σ*_C2−C7_ → *σ*^*^_C2−C6_ | 0.60 | 0.60 | 0.60 | 0.62 | 0.63 |
|  |  | *σ*^*^_C5−C11_ | 0.00691 | *σ*_C2−C7_ → *σ*^*^_C5−C11_ | 1.25 | 1.24 | 1.20 | 1.28 | 1.34 |
|  |  | *σ*^*^_C6−H15_ | 0.00347 | *σ*_C2−C7_ → *σ*^*^_C6−H15_ | 1.06 | 1.06 | 1.07 | 1.04 | 1.06 |
| *σ*_C5−C11_ | 0.99058 | *σ*^*^_C2−C7_ | 0.00900 | *σ*_C5−C11_ → *σ*^*^_C2−C7_ | 1.28 | 1.18 | 1.19 | 1.19 | 1.18 |
|  |  | *σ*^*^_C10−H26_ | 0.00603 | *σ*_C5−C11_ → *σ*^*^_C10−H26_ | 1.14 | 1.22 | 1.19 | 1.14 | 1.06 |
| *σ*_C5−H12_ | 0.98884 | *σ*^*^_N1−C2_ | 0.03742 | *σ*_C5−C12_ → *σ*^*^_N1−C2_ | 2.51 | 2.50 | 2.49 | 2.45 | 2.40 |
|  |  | *σ*^*^_C10−C11_ | 0.00691 | *σ*_C5−C12_ → *σ*^*^_C10−C11_ | 1.49 | 1.60 | 1.52 | 1.53 | 1.62 |
| *σ*_C6−H14_ | 0.99117 | *σ*^*^_N1−C2_ | 0.03742 | *σ*_C6−C14_ → *σ*^*^_N1−C2_ | 2.65 | 2.64 | 2.65 | 2.69 | 2.72 |
| *σ*_C6−H15_ | 0.99403 | *σ*^*^_N1−C2_ | 0.03742 | *σ*_C6−C15_ → *σ*^*^_N1−C2_ | 0.42 | 0.42 | 0.41 | 0.42 | 0.41 |
|  |  | *σ*^*^_C2−C5_ | 0.01507 | *σ*_C6−C15_→ *σ*^*^_C2−C5_ | 2.09 | 2.13 | 2.15 | 2.17 | 2.21 |
|  |  | *σ*^*^_C2−C7_ | 0.00900 | *σ*_C6−C15_ → *σ*^*^_C2−C7_ | 1.81 | 1.81 | 1.81 | 1.82 | 1.81 |
| *σ*_C11−H28_ | 0.98970 | *σ*^*^_C5−H13_ | 0.00781 | *σ*_C11−H28_ → *σ*^*^_C5−H13_ | 1.54 | 1.72 | 1.57 | 1.52 | 1.33 |
| LP(1)_O3_ | 0.99128 | *σ*^*^_N1−C2_ | 0.03742 | LP(1)_O3_ → *σ*^*^_N1−C2_ | 1.88 | 1.88 | 1.89 | 1.89 | 1.91 |
| LP(2)_O3_ | 0.96445 | *σ*^*^_N1−C2_ | 0.03742 | LP(2)_O3_ → *σ*^*^_N1−C2_ | 5.84 | 5.84 | 5.86 | 5.87 | 5.92 |
|  |  | *σ*^*^_C4−C10_ | 0.01507 | LP(2)_O3_ → *σ*^*^_C4−C10_ | 0.65 | 0.65 | 0.65 | 0.66 | 0.68 |

**Table S11.** Summary of important second-order delocalization energies (*E*_2_) for the orbital interactions of protonated TEMPO derivatives (**O-protonation**), determined using NBO analysis at the B3LYP/6-311++G(d,p) level of theory.

| Donor  NBO (*i*) | ED(*i*) (a.u.), X=H | Acceptor  NBO(*j*) | ED(*j*) (a.u.), X=H | Interaction type | *E*_2_ (kcal/mol) | | | | |
| --- | --- | --- | --- | --- | --- | --- | --- | --- | --- |
|  |  |  |  |  | −H | −CH_3_ | −NH_2_ | −CHO | −NO_2_ |
| *σ*_N1−C2_ | 0.99147 | *σ*^*^_N1−C4_ | 0.03421 | *σ* _N1−C2_ → *σ*^*^_N1−C4_ | 0.41 | 0.41 | 0.42 | 0.41 | 0.39 |
|  |  | *σ*^*^_C4−C8_ | 0.00847 | *σ* _N1−C2_ → *σ*^*^_C4−C8_ | 0.26 | 0.30 | 0.26 | 0.30 | 0.30 |
|  |  | *σ*^*^_C5−H12_ | 0.00547 | *σ* _N1−C2_ → *σ*^*^_C5−H12_ | 0.55 | 0.57 | 0.58 | 0.55 | 0.52 |
|  |  | *σ*^*^_C6−H14_ | 0.00236 | *σ* _N1−C2_ → *σ*^*^_C6−H14_ | 0.50 | 0.49 | 0.49 | 0.49 | 0.48 |
| *σ*_N1−O3_ | 0.99688 | *σ*^*^_C2−C5_ | 0.01468 | *σ*_N1−O3_ → *σ*^*^_C2−C5_ | 0.34 | 0.35 | 0.35 | 0.35 | 0.34 |
| π_N1−O3_ | 0.99498 | *σ*^*^_C2−C6_ | 0.00996 | π_N1−O3_ → *σ*^*^_C2−C6_ | 0.43 | 0.42 | 0.42 | 0.42 | 0.42 |
| *σ*_C2−C5_ | 0.97992 | *σ*^*^_N1−O3_ | 0.01509 | *σ*_C2−C5_ → *σ*^*^_N1−O3_ | 1.94 | 1.79 | 1.96 | 1.74 | 1.69 |
|  |  | π^*^_N1−O3_ | 0.10008 | *σ*_C2−C5_ → π^*^_N1−O3_ | 0.28 | 0.35 | <0.25 | 0.38 | 0.38 |
|  |  | *σ*^*^_C2−C6_ | 0.00996 | *σ*_C2−C5_ → *σ*^*^_C2−C6_ | 0.53 | 0.53 | 0.57 | 0.50 | 0.49 |
|  |  | *σ*^*^_C5−C11_ | 0.00616 | *σ*_C2−C5_ → *σ*^*^_C5−C11_ | 0.41 | 0.48 | 0.40 | 0.51 | 0.51 |
|  |  | *σ*^*^_C6−H16_ | 0.00254 | *σ*_C2−C5_ → *σ*^*^_C6−H16_ | 0.69 | 0.66 | 0.68 | 0.64 | 0.62 |
|  |  | *σ*^*^_C7−H18_ | 0.00325 | *σ*_C2−C5_ → *σ*^*^_C7−H18_ | 0.84 | 0.70 | 0.82 | 0.69 | 0.69 |
| *σ*_C2−C6_ | 0.96225 | π^*^_N1−O3_ | 0.10008 | *σ*_C2−C6_ → π^*^_N1−O3_ | 3.50 | 3.39 | 3.53 | 3.36 | 3.27 |
|  |  | *σ*^*^_N1−C4_ | 0.03421 | *σ*_C2−C6_ → *σ*^*^_N1−C4_ | 0.51 | 0.58 | 0.52 | 0.57 | 0.57 |
|  |  | *σ*^*^_C2−C5_ | 0.01468 | *σ*_C2−C6_ → *σ*^*^_C2−C5_ | 0.55 | 0.53 | 0.57 | 0.51 | 0.47 |
|  |  | *σ*^*^_C2−C7_ | 0.00934 | *σ*_C2−C6_ →*σ*^*^_C2−C7_ | 0.37 | 0.42 | 0.38 | 0.44 | 0.46 |
|  |  | *σ*^*^_C5−H13_ | 0.00724 | *σ*_C2−C6_ →*σ*^*^_C5−H13_ | 0.77 | 0.82 | 0.81 | 0.83 | 0.79 |
|  |  | *σ*^*^_C7−H19_ | 0.00262 | *σ*_C2−C6_ →*σ*^*^_C7−H19_ | 0.81 | 0.79 | 0.80 | 0.78 | 0.77 |
| *σ*_C2−C7_ | 0.98134 | π^*^_N1−O3_ | 0.10008 | *σ*_C2−C7_ → π^*^_N1−O3_ | 0.53 | 0.85 | 0.58 | 0.80 | 0.80 |
|  |  | *σ*^*^_N1−C4_ | 0.03421 | *σ*_C2−C7_ → *σ*^*^_N1−C4_ | 1.54 | 1.64 | 1.54 | 1.65 | 1.64 |
|  |  | *σ*^*^_C2−C5_ | 0.01468 | *σ*_C2−C7_ → *σ*^*^_C2−C5_ | 0.59 | 0.63 | 0.65 | 0.62 | 0.64 |
|  |  | *σ*^*^_C2−C6_ | 0.00996 | *σ*_C2−C7_ → *σ*^*^_C2−C6_ | 0.48 | 0.52 | 0.48 | 0.53 | 0.54 |
|  |  | *σ*^*^_C5−C11_ | 0.00616 | *σ*_C2−C7_ → *σ*^*^_C5−C11_ | 0.91 | 0.97 | 0.91 | 0.99 | 1.07 |
|  |  | *σ*^*^_C6−H15_ | 0.00306 | *σ*_C2−C7_ → *σ*^*^_C6−H15_ | 0.79 | 0.82 | 0.79 | 0.81 | 0.83 |
| *σ*_C5−C11_ | 0.99033 | *σ*^*^_C2−C5_ | 0.01468 | *σ*_C5−C11_ → *σ*^*^_C2−C5_ | 0.40 | 0.41 | 0.39 | 0.34 | 0.40 |
|  |  | *σ*^*^_C2−C7_ | 0.00934 | *σ*_C5−C11_ → *σ*^*^_C2−C7_ | 1.23 | 1.03 | 1.14 | 1.03 | 1.01 |
|  |  | *σ*^*^_C10−C11_ | 0.00616 | *σ*_C5−C11_ → *σ*^*^_C10−C11_ | 0.32 | 0.37 | 0.46 | 0.42 | 0.53 |
|  |  | *σ*^*^_C10−H26_ | 0.00533 | *σ*_C5−C11_ → *σ*^*^_C10−H26_ | 0.90 | 1.01 | 0.97 | 0.93 | 0.81 |
| *σ*_C5−H12_ | 0.98609 | *σ*^*^_N1−C2_ | 0.03710 | *σ*_C5−C12_ → *σ*^*^_N1−C2_ | 2.45 | 2.44 | 2.48 | 2.41 | 2.41 |
|  |  | *σ*^*^_C10−C11_ | 0.00616 | *σ*_C5−C12_ → *σ*^*^_C10−C11_ | 1.33 | 1.45 | 1.30 | 1.41 | 1.54 |
| *σ*_C5−C13_ | 0.98846 | *σ*^*^_C2−C6_ | 0.00996 | *σ*_C5−C13_ → *σ*^*^_C2−C6_ | 1.97 | 1.95 | 2.01 | 1.90 | 1.90 |
| *σ*_C6−H14_ | 0.98353 | *σ*^*^_N1−C2_ | 0.03710 | *σ*_C6−C14_ → *σ*^*^_N1−C2_ | 2.72 | 2.66 | 2.71 | 2.70 | 2.72 |
|  |  | π^*^_N1−O3_ | 0.10008 | *σ*_C6−C14_ → π^*^_N1−O3_ | 0.49 | 0.50 | 0.48 | 0.51 | 0.51 |
| *σ*_C6−H15_ | 0.99329 | *σ*^*^_C2−C5_ | 0.01468 | *σ*_C6−C15_ → *σ*^*^_C2−C5_ | 1.74 | 1.81 | 1.76 | 1.84 | 1.86 |
|  |  | *σ*^*^_C2−C7_ | 0.00934 | *σ*_C6−C15_ → *σ*^*^_C2−C7_ | 1.73 | 1.65 | 1.72 | 1.66 | 1.64 |
| *σ*_C11−H28_ | 0.98931 | *σ*^*^_C5−H13_ | 0.00724 | *σ*_C11−H28_ → *σ*^*^_C5−H13_ | 1.32 | 1.48 | 1.33 | 1.34 | 1.24 |
| LP(1)_O3_ | 0.99033 | *σ*^*^_N1−C2_ | 0.03710 | LP(1)_O3_ → *σ*^*^_N1−C2_ | 2.99 | 2.98 | 3.00 | 3.01 | 3.05 |
|  |  | *σ*^*^_C4−C10_ | 0.01431 | LP(1)_O3_ → *σ*^*^_C4−C10_ | 0.28 | 0.29 | 0.29 | 0.29 | 0.29 |

**Table S12.** Summary of important second-order delocalization energies (*E*_2_) for the orbital interactions of protonated TEMPO derivatives (**O-protonation**), determined using NBO analysis at the M06-2X/6-311++G(d,p) level of theory.

| Donor  NBO (*i*) | ED(*i*) (a.u.), X=H | Acceptor  NBO(*j*) | ED(*j*) (a.u.), X=H | Interaction type | *E*_2_ (kcal/mol) | | | | |
| --- | --- | --- | --- | --- | --- | --- | --- | --- | --- |
|  |  |  |  |  | −H | −CH_3_ | −NH_2_ | −CHO | −NO_2_ |
| *σ*_N1−C2_ | 0.99138 | *σ*^*^_N1−C4_ | 0.03341 | *σ* _N1−C2_ → *σ*^*^_N1−C4_ | 0.50 | 0.48 | 0.47 | 0.50 | 0.34 |
|  |  | *σ*^*^_C4−C8_ | 0.00868 | *σ* _N1−C2_ → *σ*^*^_C4−C8_ | 0.30 | 0.35 | 0.35 | 0.31 | 0.33 |
|  |  | *σ*^*^_C5−H12_ | 0.00546 | *σ* _N1−C2_ → *σ*^*^_C5−H12_ | 0.62 | 0.65 | 0.66 | 0.63 | 0.57 |
|  |  | *σ*^*^_C6−H14_ | 0.00244 | *σ* _N1−C2_ → *σ*^*^_C6−H14_ | 0.55 | 0.56 | 0.56 | 0.54 | 0.53 |
| *σ*_N1−O3_ | 0.99690 | *σ*^*^_C2−C5_ | 0.01454 | *σ*_N1−O3_ → *σ*^*^_C2−C5_ | 0.28 | 0.37 | 0.36 | 0.29 | 0.36 |
| π_N1−O3_ | 0.99528 | *σ*^*^_C2−C6_ | 0.01000 | π_N1−O3_ → *σ*^*^_C2−C6_ | 0.46 | 0.45 | 0.45 | 0.47 | 0.44 |
| *σ*_C2−C5_ | 0.98023 | *σ*^*^_N1−O3_ | 0.01391 | *σ*_C2−C5_ → *σ*^*^_N1−O3_ | 2.28 | 2.05 | 2.06 | 2.22 | 1.95 |
|  |  | π^*^_N1−O3_ | 0.09012 | *σ*_C2−C5_ → π^*^_N1−O3_ | 0.50 | 0.59 | 0.55 | 0.51 | 0.54 |
|  |  | *σ*^*^_C2−C6_ | 0.01000 | *σ*_C2−C5_ → *σ*^*^_C2−C6_ | 0.69 | 0.69 | 0.70 | 0.70 | 0.57 |
|  |  | *σ*^*^_C5−C11_ | 0.00626 | *σ*_C2−C5_ → *σ*^*^_C5−C11_ | 0.52 | 0.61 | 0.52 | 0.63 | 0.60 |
|  |  | *σ*^*^_C6−H16_ | 0.00249 | *σ*_C2−C5_ → *σ*^*^_C6−H16_ | 0.79 | 0.75 | 0.73 | 0.77 | 0.70 |
|  |  | *σ*^*^_C7−H18_ | 0.00315 | *σ*_C2−C5_ → *σ*^*^_C7−H18_ | 0.93 | 0.79 | 0.79 | 0.90 | 0.76 |
| *σ*_C2−C6_ | 0.96331 | π^*^_N1−O3_ | 0.09012 | *σ*_C2−C6_ → π^*^_N1−O3_ | 4.88 | 4.89 | 4.83 | 4.84 | 4.45 |
|  |  | *σ*^*^_N1−C4_ | 0.03341 | *σ*_C2−C6_ → *σ*^*^_N1−C4_ | 0.57 | 0.64 | 0.66 | 0.57 | 0.65 |
|  |  | *σ*^*^_C2−C5_ | 0.01454 | *σ*_C2−C6_ → *σ*^*^_C2−C5_ | 0.72 | 0.69 | 0.67 | 0.74 | 0.54 |
|  |  | *σ*^*^_C2−C7_ | 0.00949 | *σ*_C2−C6_ →*σ*^*^_C2−C7_ | 0.50 | 0.56 | 0.57 | 0.52 | 0.54 |
|  |  | *σ*^*^_C5−H13_ | 0.00710 | *σ*_C2−C6_ →*σ*^*^_C5−H13_ | 0.90 | 0.98 | 0.96 | 0.97 | 0.89 |
|  |  | *σ*^*^_C7−H19_ | 0.00257 | *σ*_C2−C6_ →*σ*^*^_C7−H19_ | 0.90 | 0.88 | 0.87 | 0.89 | 0.85 |
| *σ*_C2−C7_ | 0.98198 | π^*^_N1−O3_ | 0.09012 | *σ*_C2−C7_ → π^*^_N1−O3_ | 0.66 | 1.00 | 1.04 | 0.65 | 1.10 |
|  |  | *σ*^*^_N1−C4_ | 0.03341 | *σ*_C2−C7_ → *σ*^*^_N1−C4_ | 1.83 | 1.95 | 1.95 | 1.83 | 1.85 |
|  |  | *σ*^*^_C2−C5_ | 0.01454 | *σ*_C2−C7_ → *σ*^*^_C2−C5_ | 0.74 | 0.80 | 0.82 | 0.78 | 0.73 |
|  |  | *σ*^*^_C2−C6_ | 0.01000 | *σ*_C2−C7_ → *σ*^*^_C2−C6_ | 0.64 | 0.69 | 0.69 | 0.65 | 0.64 |
|  |  | *σ*^*^_C5−C11_ | 0.00626 | *σ*_C2−C7_ → *σ*^*^_C5−C11_ | 1.02 | 1.08 | 1.07 | 1.05 | 1.18 |
|  |  | *σ*^*^_C6−H15_ | 0.00313 | *σ*_C2−C7_ → *σ*^*^_C6−H15_ | 0.91 | 0.94 | 0.95 | 0.89 | 0.93 |
| *σ*_C5−C11_ | 0.99040 | *σ*^*^_C2−C5_ | 0.01454 | *σ*_C5−C11_ → *σ*^*^_C2−C5_ | 0.52 | 0.55 | 0.51 | 0.48 | 0.48 |
|  |  | *σ*^*^_C2−C7_ | 0.00949 | *σ*_C5−C11_ → *σ*^*^_C2−C7_ | 1.44 | 1.23 | 1.23 | 1.33 | 1.11 |
|  |  | *σ*^*^_C10−C11_ | 0.00624 | *σ*_C5−C11_ → *σ*^*^_C10−C11_ | 0.42 | 0.49 | 0.59 | 0.54 | 0.61 |
|  |  | *σ*^*^_C10−H26_ | 0.00534 | *σ*_C5−C11_ → *σ*^*^_C10−H26_ | 1.04 | 1.16 | 1.13 | 1.07 | 0.90 |
| *σ*_C5−H12_ | 0.98660 | *σ*^*^_N1−C2_ | 0.03587 | *σ*_C5−C12_ → *σ*^*^_N1−C2_ | 2.89 | 2.88 | 2.88 | 2.85 | 2.80 |
|  |  | *σ*^*^_C10−C11_ | 0.00624 | *σ*_C5−C12_ → *σ*^*^_C10−C11_ | 1.47 | 1.64 | 1.53 | 1.54 | 1.73 |
| *σ*_C5−C13_ | 0.98877 | *σ*^*^_C2−C6_ | 0.01000 | *σ*_C5−C13_ → *σ*^*^_C2−C6_ | 2.28 | 2.25 | 2.28 | 2.21 | 2.11 |
| *σ*_C6−H14_ | 0.98486 | *σ*^*^_N1−C2_ | 0.03587 | *σ*_C6−C14_ → *σ*^*^_N1−C2_ | 3.15 | 3.10 | 3.11 | 3.19 | 3.18 |
|  |  | π^*^_N1−O3_ | 0.09012 | *σ*_C6−C14_ → π^*^_N1−O3_ | 0.65 | 0.66 | 0.66 | 0.65 | 0.65 |
| *σ*_C6−H15_ | 0.99349 | *σ*^*^_C2−C5_ | 0.01454 | *σ*_C6−C15_ → *σ*^*^_C2−C5_ | 1.99 | 2.08 | 2.09 | 2.05 | 2.10 |
|  |  | *σ*^*^_C2−C7_ | 0.00949 | *σ*_C6−C15_ → *σ*^*^_C2−C7_ | 1.95 | 1.87 | 1.87 | 1.96 | 1.84 |
| *σ*_C11−H28_ | 0.98965 | *σ*^*^_C5−H13_ | 0.00710 | *σ*_C11−H28_ → *σ*^*^_C5−H13_ | 1.52 | 1.69 | 1.50 | 1.36 | 1.39 |
| LP(1)_O3_ | 0.99049 | *σ*^*^_N1−C2_ | 0.03587 | LP(1)_O3_ → *σ*^*^_N1−C2_ | 3.60 | 3.59 | 3.60 | 3.62 | 3.46 |
|  |  | *σ*^*^_C4−C10_ | 0.01426 | LP(1)_O3_ → *σ*^*^_C4−C10_ | < 0.25 | 0.34 | 0.33 | 0.34 | 0.32 |

**Table S13.** Summary of important second-order delocalization energies (*E*_2_) for the orbital interactions of protonated TEMPO derivatives (**N-protonation**), determined using NBO analysis at the B3LYP/6-311++G(d,p) level of theory.

| Donor  NBO (*i*) | ED(*i*) (a.u.), X=H | Acceptor  NBO(*j*) | ED(*j*) (a.u.), X=H | Interaction type | *E*_2_ (kcal/mol) | | | | |
| --- | --- | --- | --- | --- | --- | --- | --- | --- | --- |
|  |  |  |  |  | −H | −CH_3_ | −NH_2_ | −CHO | −NO_2_ |
| *σ*_N1−C2_ | 0.99204 | *σ*^*^_N1−O3_ | 0.00507 | *σ* _N1−C2_ → *σ*^*^_N1−O3_ | 0.33 | 0.35 | 0.35 | 0.31 | 0.32 |
|  |  | *σ*^*^_C5−H12_ | 0.00493 | *σ* _N1−C2_ → *σ*^*^_C5−H12_ | 0.65 | 0.67 | 0.68 | 0.65 | 0.61 |
|  |  | *σ*^*^_C6−H14_ | 0.00233 | *σ* _N1−C2_ → *σ*^*^_C6−H14_ | 0.58 | 0.57 | 0.57 | 0.56 | 0.56 |
| *σ*_N1−O3_ |  | *σ*^*^_N1−C2_ | 0.01599 | *σ*_N1−O3_ → *σ*^*^_N1−C2_ | 0.26 | 0.26 | 0.26 | 0.27 | 0.26 |
|  |  | *σ*^*^_N1−O3_ | 0.00507 | *σ*_N1−O3_ → *σ*^*^_N1−O3_ | 0.35 | 0.36 | 0.36 | 0.33 | 0.35 |
|  |  | *σ*^*^_C2−C5_ | 0.01599 | *σ*_N1−O3_ → *σ*^*^_C2−C5_ | 0.26 | 0.28 | 0.28 | 0.27 | 0.27 |
| π_N1−O3_ | 0.96862 | *σ*^*^_C2−C6_ | 0.01131 | π_N1−O3_ → *σ*^*^_C2−C5_ | 0.48 | 0.46 | 0.46 | 0.50 | 0.57 |
|  |  | *σ*^*^_C2−C7_ | 0.00922 | π_N1−O3_ → *σ*^*^_C2−C7_ | 1.05 | 1.05 | 1.05 | 1.04 | 1.06 |
| *σ*_N1−H29_ | 0.99146 | π^*^_N1−O3_ | 0.03001 | *σ*_N1−H29_ → π^*^_N1−O3_ | 0.50 | 0.53 | 0.54 | 0.48 | 0.48 |
|  |  | *σ*^*^_C2−C6_ | 0.01131 | *σ*_N1−H29_ → *σ*^*^_C2−C6_ | 0.98 | 0.99 | 0.99 | 0.98 | 0.98 |
| *σ*_C2−C5_ | 0.98367 | *σ*^*^_N1−O3_ | 0.00507 | *σ*_C2−C5_ → *σ*^*^_N1−O3_ | 1.50 | 1.51 | 1.50 | 1.47 | 1.37 |
|  |  | *σ*^*^_C2−C6_ | 0.01131 | *σ*_C2−C5_ → *σ*^*^_C2−C6_ | 0.51 | 0.53 | 0.53 | 0.52 | 0.47 |
|  |  | *σ*^*^_C5−C11_ | 0.00696 | *σ*_C2−C5_ → *σ*^*^_C5−C11_ | 0.38 | 0.46 | 0.40 | 0.49 | 0.47 |
|  |  | *σ*^*^_C6−H16_ | 0.00272 | *σ*_C2−C5_ → *σ*^*^_C6−H16_ | 0.68 | 0.67 | 0.66 | 0.66 | 0.65 |
| *σ*_C2−C6_ | 0.98663 | *σ*^*^_N1−H30_ | 0.02648 | *σ*_C2−C6_ → *σ*^*^_N1−H30_ | 1.31 | 1.30 | 1.29 | 1.31 | 1.31 |
|  |  | *σ*^*^_C2−C5_ | 0.01599 | *σ*_C2−C6_ → *σ*^*^_C2−C5_ | 0.56 | 0.60 | 0.58 | 0.59 | 0.54 |
|  |  | *σ*^*^_C2−C7_ | 0.00922 | *σ*_C2−C6_ → *σ*^*^_C2−C7_ | 0.50 | 0.49 | 0.50 | 0.51 | 0.54 |
|  |  | *σ*^*^_C5−H13_ | 0.00725 | *σ*_C2−C6_ → *σ*^*^_C5−H13_ | 0.83 | 0.88 | 0.86 | 0.85 | 0.86 |
|  |  | *σ*^*^_C7−H19_ | 0.00278 | *σ*_C2−C6_ → *σ*^*^_C7−H19_ | 0.78 | 0.78 | 0.78 | 0.76 | 0.76 |
| *σ*_C2−C7_ | 0.97279 | π^*^_N1−O3_ | 0.03001 | *σ*_C2−C7_ → π^*^_N1−O3_ | 2.58 | 2.61 | 2.58 | 2.61 | 2.50 |
|  |  | *σ*^*^_C2−C6_ | 0.01131 | *σ*_C2−C7_ → *σ*^*^_C2−C6_ | 0.52 | 0.51 | 0.51 | 0.52 | 0.55 |
|  |  | *σ*^*^_C5−C11_ | 0.00696 | *σ*_C2−C7_ → *σ*^*^_C5−C11_ | 1.27 | 1.29 | 1.27 | 1.33 | 1.40 |
|  |  | *σ*^*^_C6−H15_ | 0.00313 | *σ*_C2−C7_ → *σ*^*^_C6−H15_ | 0.90 | 0.90 | 0.91 | 0.90 | 0.90 |
| *σ*_C5−C11_ | 0.99034 | *σ*^*^_C2−C5_ | 0.01599 | *σ*_C5−C11_ → *σ*^*^_C2−C5_ | 0.45 | 0.49 | 0.46 | 0.47 | 0.46 |
|  |  | *σ*^*^_C2−C7_ | 0.00922 | *σ*_C5−C11_ → *σ*^*^_C2−C7_ | 0.96 | 0.88 | 0.89 | 0.88 | 0.84 |
|  |  | *σ*^*^_C10−H26_ | 0.00511 | *σ*_C5−C11_ → *σ*^*^_C10−H26_ | 0.61 | 0.71 | 0.66 | 0.64 | 0.61 |
| *σ*_C5−H12_ | 0.98385 | *σ*^*^_N1−C2_ | 0.04401 | *σ*_C5−C12_ → *σ*^*^_N1−C2_ | 2.54 | 2.59 | 2.58 | 2.54 | 2.48 |
|  |  | *σ*^*^_C10−C11_ | 0.00767 | *σ*_C5−C12_ → *σ*^*^_C10−C11_ | 1.75 | 1.78 | 1.69 | 1.93 | 1.77 |
| *σ*_C5−C13_ | 0.98844 | *σ*^*^_C2−C6_ | 0.01131 | *σ*_C5−C13_ → *σ*^*^_C2−C6_ | 2.06 | 2.07 | 2.08 | 2.07 | 1.95 |
|  |  | *σ*^*^_C11−H28_ | 0.00730 | *σ*_C5−C13_ → *σ*^*^_C11−H28_ | 1.43 | 1.51 | 1.44 | 1.63 | 1.55 |
| *σ*_C6−H15_ | 0.99261 | *σ*^*^_N1−C2_ | 0.04401 | *σ*_C6−C15_ → *σ*^*^_N1−C2_ | 2.86 | 2.88 | 2.87 | 2.87 | 2.85 |
|  |  | *σ*^*^_C2−C5_ | 0.01599 | *σ*_C6−C15_→ *σ*^*^_C2−C5_ | 2.09 | 2.12 | 2.13 | 2.13 | 2.15 |
|  |  | *σ*^*^_C2−C7_ | 0.00922 | *σ*_C6−C15_ → *σ*^*^_C2−C7_ | 1.78 | 1.78 | 1.76 | 1.78 | 1.77 |
| *σ*_C11−H28_ | 0.98958 | *σ*^*^_C5−H13_ | 0.00725 | *σ*_C11−H28_ → *σ*^*^_C5−H13_ | 1.29 | 1.42 | 1.30 | 1.28 | 1.24 |
| LP(1)_O3_ | 0.99276 | *σ*^*^_N1−C2_ | 0.04401 | LP(1)_O3_ → *σ*^*^_N1−C2_ | 1.43 | 1.42 | 1.41 | 1.44 | 1.44 |
|  |  | *σ*^*^_N1−H30_ | 0.02648 | LP(1)_O3_ → *σ*^*^_N1−H30_ | 0.46 | 0.45 | 0.45 | 0.46 | 0.45 |
| LP(2)_O3_ | 0.95768 | *σ*^*^_N1−C2_ | 0.04401 | LP(2)_O3_ → *σ*^*^_N1−C2_ | 5.62 | 5.54 | 5.59 | 5.69 | 5.74 |
|  |  | *σ*^*^_N1−H30_ | 0.02648 | LP(2)_O3_ → *σ*^*^_N1−H30_ | 6.73 | 6.64 | 6.61 | 6.84 | 6.77 |
|  |  | *σ*^*^_C4−C10_ | 0.01599 | LP(2)_O3_ → *σ*^*^_C4−C10_ | 0.26 | 0.27 | 0.27 | 0.25 | 0.26 |

**Table S14.** Summary of important second-order delocalization energies (*E*_2_) for the orbital interactions of protonated TEMPO derivatives (**N-protonation**), determined using NBO analysis at the M06-2X/6-311++G(d,p) level of theory.

| Donor  NBO (*i*) | ED(*i*) (a.u.), X=H | Acceptor  NBO(*j*) | ED(*j*) (a.u.), X=H | Interaction type | *E*_2_ (kcal/mol) | | | | |
| --- | --- | --- | --- | --- | --- | --- | --- | --- | --- |
|  |  |  |  |  | −H | −CH_3_ | −NH_2_ | −CHO | −NO_2_ |
| *σ*_N1−C2_ | 0.94308 | *σ*^*^_C5−H12_ | 0.00527 | *σ* _N1−C2_ → *σ*^*^_C5−H12_ | 0.71 | 0.75 | 0.77 | 0.70 | 0.63 |
|  |  | *σ*^*^_C6−H14_ | 0.00262 | *σ* _N1−C2_ → *σ*^*^_C6−H14_ | 0.54 | 0.53 | 0.53 | 0.48 | 0.53 |
| *σ*_N1−O3_ | 0.99654 | *σ*^*^_N1−C2_ | 0.04963 | *σ*_N1−O3_ → *σ*^*^_N1−C2_ | 0.33 | 0.33 | 0.33 | 0.31 | 0.34 |
|  |  | *σ*^*^_C2−C5_ | 0.01484 | *σ*_N1−O3_ → *σ*^*^_C2−C5_ | 0.32 | 0.33 | 0.32 | 0.42 | 0.33 |
| *σ*_N1−H29_ | 0.98630 | *σ*^*^_C2−C6_ | 0.01192 | *σ*_N1−H29_ → *σ*^*^_C2−C6_ | 1.09 | 1.10 | 1.09 | 1.20 | 1.07 |
| *σ*_C2−C5_ | 0.98168 | *σ*^*^_N1−O3_ | 0.01018 | *σ*_C2−C5_ → *σ*^*^_N1−O3_ | 1.18 | 1.18 | 1.18 | 1.60 | 1.12 |
|  |  | *σ*^*^_C2−C6_ | 0.01192 | *σ*_C2−C5_ → *σ*^*^_C2−C6_ | 0.54 | 0.57 | 0.57 | 0.62 | 0.52 |
|  |  | *σ*^*^_C5−C11_ | 0.00661 | *σ*_C2−C5_ → *σ*^*^_C5−C11_ | 0.66 | 0.75 | 0.65 | 0.71 | 0.79 |
|  |  | *σ*^*^_C6−H16_ | 0.00346 | *σ*_C2−C5_ → *σ*^*^_C6−H16_ | 0.77 | 0.77 | 0.76 | 0.71 | 0.74 |
| *σ*_C2−C6_ | 0.01192 | *σ*^*^_N1−H30_ | 0.02084 | *σ*_C2−C6_ → *σ*^*^_N1−H30_ | 0.77 | 0.78 | 0.77 | 1.12 | 0.76 |
|  |  | *σ*^*^_C2−C5_ | 0.01484 | *σ*_C2−C6_ → *σ*^*^_C2−C5_ | 0.62 | 0.67 | 0.66 | 0.69 | 0.60 |
|  |  | *σ*^*^_C2−C7_ | 0.00910 | *σ*_C2−C6_ → *σ*^*^_C2−C7_ | 0.46 | 0.46 | 0.47 | 0.57 | 0.50 |
|  |  | *σ*^*^_C5−H13_ | 0.00746 | *σ*_C2−C6_ → *σ*^*^_C5−H13_ | 0.93 | 1.00 | 0.98 | 0.99 | 0.95 |
|  |  | *σ*^*^_C7−H19_ | 0.00291 | *σ*_C2−C6_ → *σ*^*^_C7−H19_ | 0.90 | 0.90 | 0.89 | 0.89 | 0.87 |
| *σ*_C2−C7_ | 0.00910 | *σ*^*^_C2−C6_ | 0.01192 | *σ*_C2−C7_ → *σ*^*^_C2−C6_ | 0.55 | 0.54 | 0.54 | 0.65 | 0.58 |
|  |  | *σ*^*^_C5−C11_ | 0.00661 | *σ*_C2−C7_ → *σ*^*^_C5−C11_ | 1.14 | 1.15 | 1.14 | 1.17 | 1.29 |
|  |  | *σ*^*^_C6−H15_ | 0.00346 | *σ*_C2−C7_ → *σ*^*^_C6−H15_ | 0.97 | 0.96 | 0.97 | 0.95 | 0.98 |
| *σ*_C5−C11_ | 0.98962 | *σ*^*^_C2−C5_ | 0.01484 | *σ*_C5−C11_ → *σ*^*^_C2−C5_ | 0.62 | 0.67 | 0.62 | 0.56 | 0.65 |
|  |  | *σ*^*^_C2−C7_ | 0.00910 | *σ*_C5−C11_ → *σ*^*^_C2−C7_ | 1.49 | 1.36 | 1.38 | 1.37 | 1.33 |
|  |  | *σ*^*^_C10−H26_ | 0.00513 | *σ*_C5−C11_ → *σ*^*^_C10−H26_ | 0.98 | 1.06 | 1.05 | 0.97 | 0.64 |
| *σ*_C5−H12_ | 0.97573 | *σ*^*^_N1−C2_ | 0.04963 | *σ*_C5−C12_ → *σ*^*^_N1−C2_ | 4.06 | 4.08 | 4.08 | 3.30 | 4.07 |
|  |  | *σ*^*^_C10−C11_ | 0.00649 | *σ*_C5−C12_ → *σ*^*^_C10−C11_ | 1.56 | 1.68 | 1.56 | 1.66 | 1.77 |
| *σ*_C5−C13_ | 0.98814 | *σ*^*^_C2−C6_ | 0.01192 | *σ*_C5−C13_ → *σ*^*^_C2−C6_ | 2.67 | 2.67 | 2.71 | 2.48 | 2.59 |
|  |  | *σ*^*^_C11−H28_ | 0.00731 | *σ*_C5−C13_ → *σ*^*^_C11−H28_ | 1.51 | 1.65 | 1.57 | 1.61 | 1.78 |
| *σ*_C6−H15_ | 0.99109 | *σ*^*^_N1−C2_ | 0.04963 | *σ*_C6−C15_ → *σ*^*^_N1−C2_ | 4.89 | 4.87 | 4.89 | 3.96 | 4.99 |
|  |  | *σ*^*^_C2−C5_ | 0.01484 | *σ*_C6−C15_→ *σ*^*^_C2−C5_ | 2.41 | 2.45 | 2.47 | 2.30 | 2.54 |
|  |  | *σ*^*^_C2−C7_ | 0.00910 | *σ*_C6−C15_ → *σ*^*^_C2−C7_ | 2.17 | 2.16 | 2.15 | 1.97 | 2.15 |
| *σ*_C11−H28_ | 0.98939 | *σ*^*^_C5−H13_ | 0.00746 | *σ*_C11−H28_ → *σ*^*^_C5−H13_ | 1.52 | 1.72 | 1.52 | 1.51 | 1.44 |
| LP(1)_O3_ | 0.99459 | *σ*^*^_N1−C2_ | 0.04963 | LP(1)_O3_ → *σ*^*^_N1−C2_ | 0.92 | 0.96 | 0.96 | 0.96 | 0.95 |
|  |  | *σ*^*^_N1−H30_ | 0.02084 | LP(1)_O3_ → *σ*^*^_N1−H30_ | < 0.25 | < 0.25 | < 0.25 | < 0.25 | < 0.25 |
| LP(2)_O3_ | 0.96082 | *σ*^*^_N1−C2_ | 0.04963 | LP(2)_O3_ → *σ*^*^_N1−C2_ | 2.46 | 2.40 | 2.49 | 2.77 | 2.67 |
|  |  | *σ*^*^_N1−H30_ | 0.02084 | LP(2)_O3_ → *σ*^*^_N1−H30_ | 4.59 | 4.65 | 4.57 | 4.28 | 4.32 |
|  |  | *σ*^*^_C4−C10_ | 0.01484 | LP(2)_O3_ → *σ*^*^_C4−C10_ | 0.36 | 0.35 | 0.36 | 0.41 | 0.40 |

| Optimized structure | X=H | X=CH_3_ | X=NH_2_ | X=CHO | X=NO_2_ |
| --- | --- | --- | --- | --- | --- |
| HOMO orbitals | **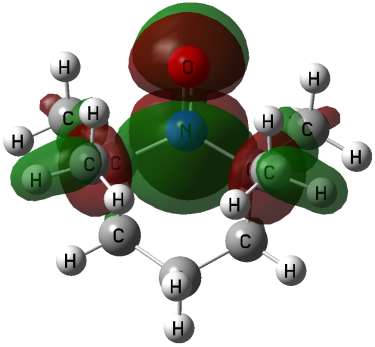**  *E* = −5.3225 (−6.9985) eV | **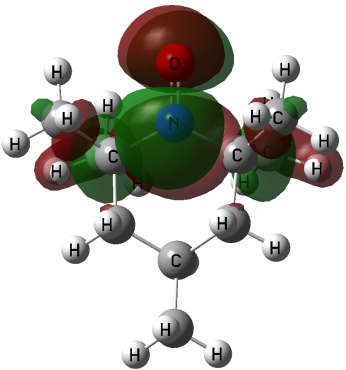**  *E* = −5.3225 (−6.9966) eV | **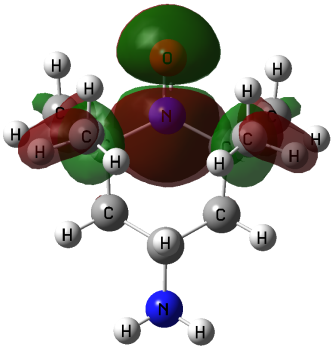**  *E* = −5.3606 (−7.0347) eV | **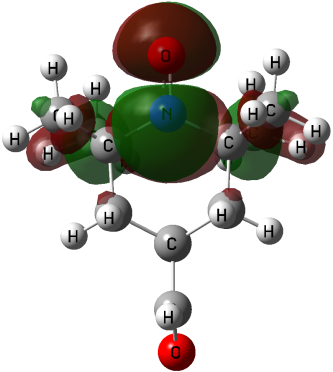**  *E* = −5.6327 (−7.3109) eV | **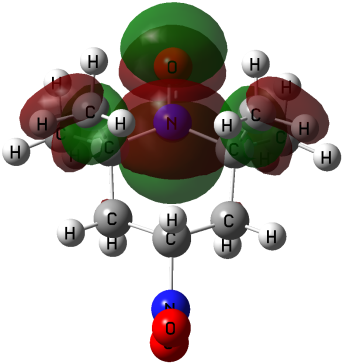**  *E* = −5.8423 (−7.5424) eV |
| LUMO orbitals | **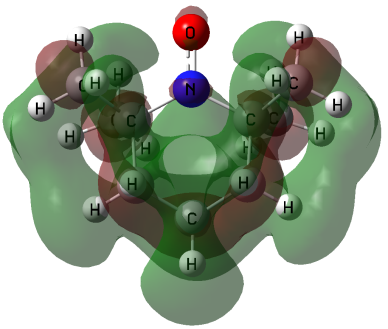**  *E* = −0.3755 (−0.1695) eV | **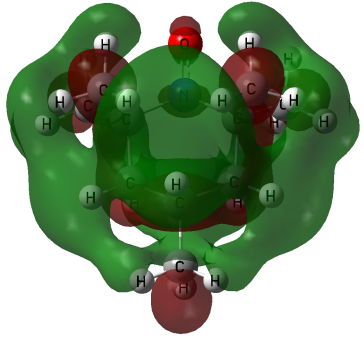**  *E* = −0.3755 (−0.1801) eV | **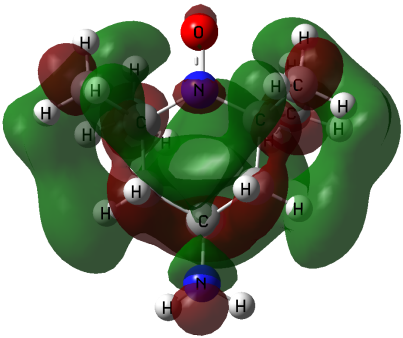**  *E* = −0.4952 (−0.2778) eV | **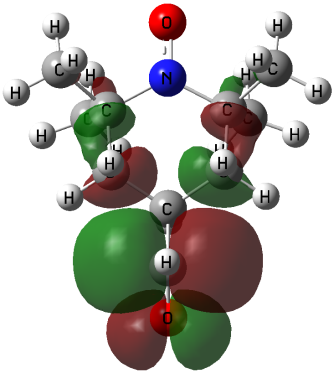**  *E* = −1.3415 (−0.1973) eV | **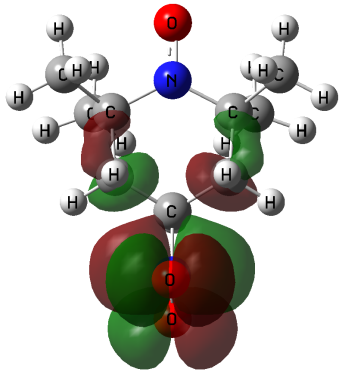**  *E* = −2.4300 (−0.8419) eV |
| MEP  maps | 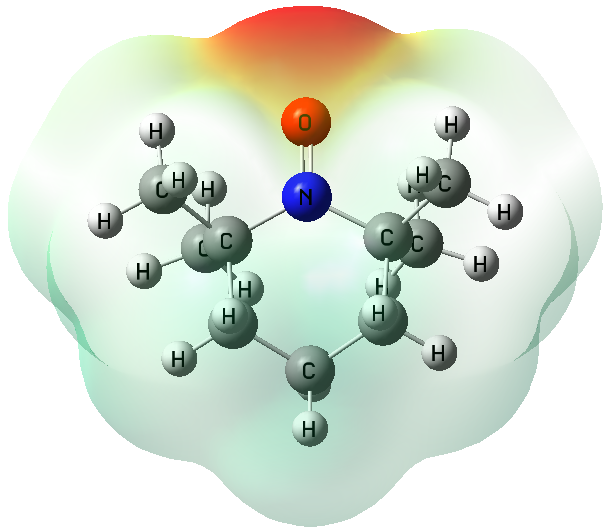 | 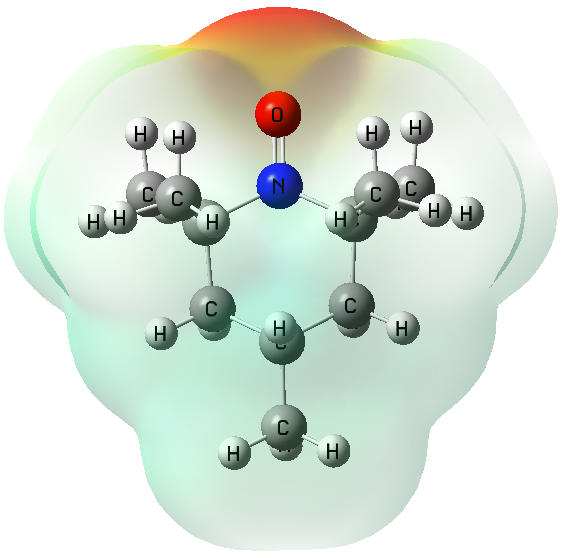 | 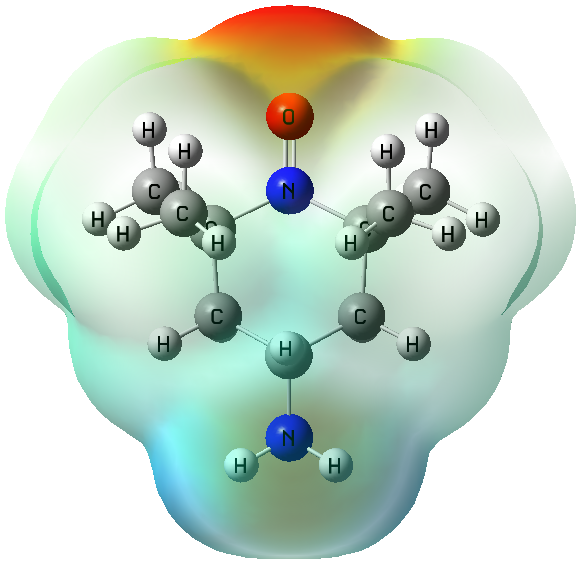 | 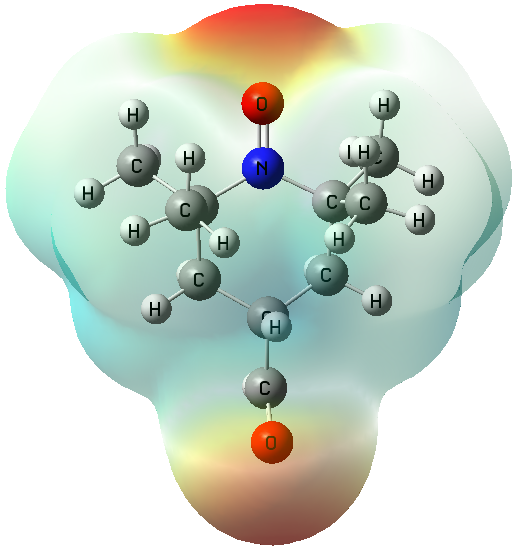 | 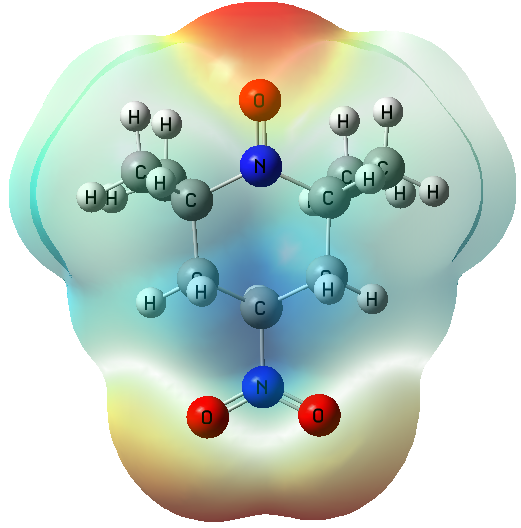 |

**Figure S1.** The representative molecular structures of the HOMO and LUMO orbitals and the total electron density mapped with the electrostatic potential surface of the considered TEMPO derivatives calculated using the B3LYP (M06-2X) theoretical method.

| Optimized structure | X=H | X=CH_3_ | X=NH_2_ | X=CHO | X=NO_2_ |
| --- | --- | --- | --- | --- | --- |
| HOMO orbitals | **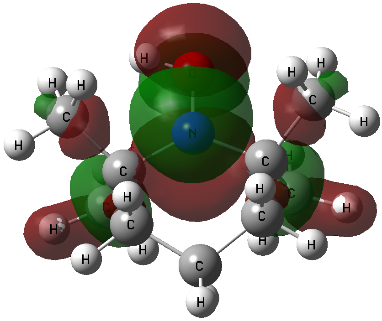**  *E* = −11.9158 (−13.5289) eV | **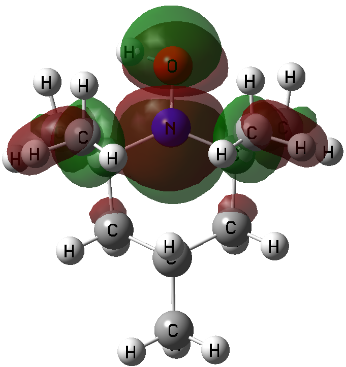**  *E* = −11.8696 (−13.4786) eV | **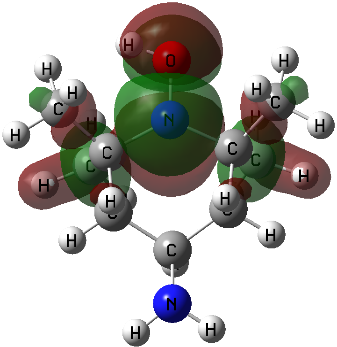**  *E* = −10.2042 (−11.9479) eV | **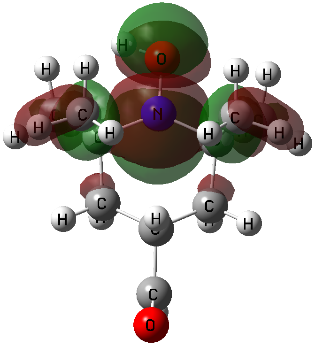**  *E* = −10.5226 (−12.3216) eV | **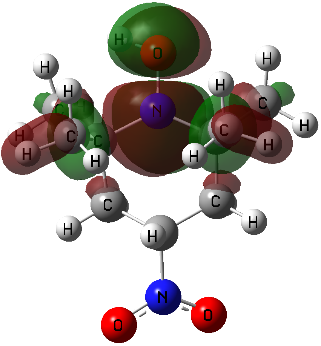**  *E* = −11.7036 (−13.6968) eV |
| LUMO orbitals | **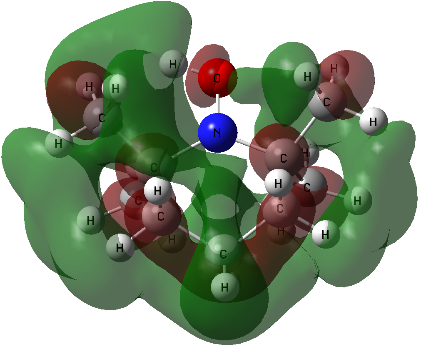**  *E* = −4.1905 (−3.5655) eV | **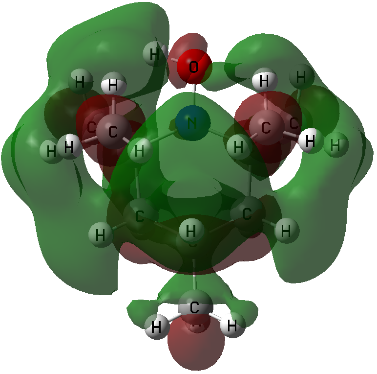**  *E* = −4.1606 (−3.5380) eV | **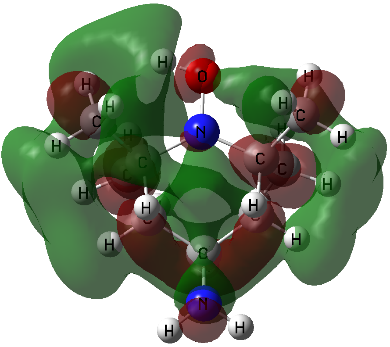**  *E* = −4.1905 (−3.5592) eV | **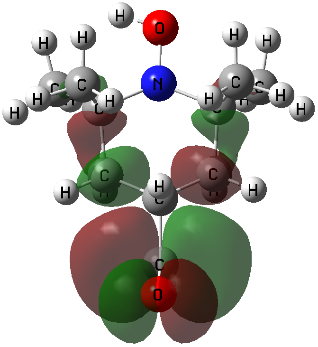**  *E* = −4.5225 (−3.7269) eV | **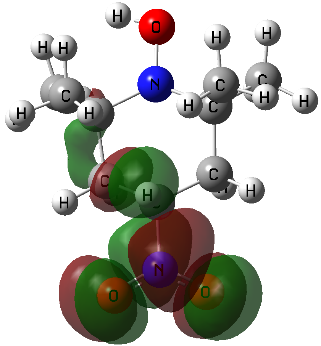**  *E* = −5.5049 (−4.0727) eV |
| MEP  maps | **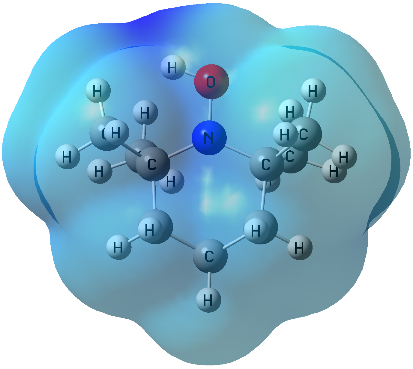** | **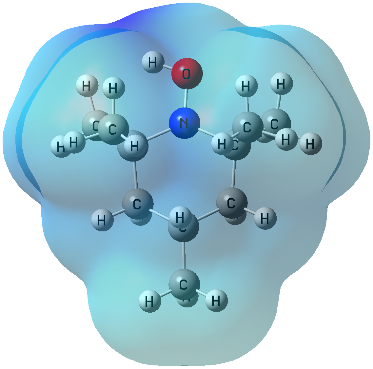** | **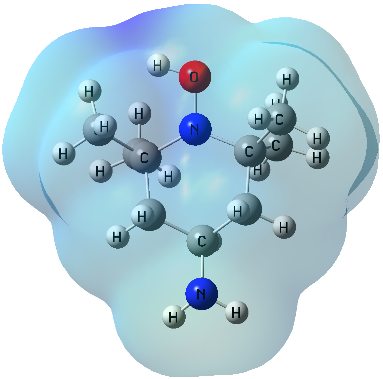** | **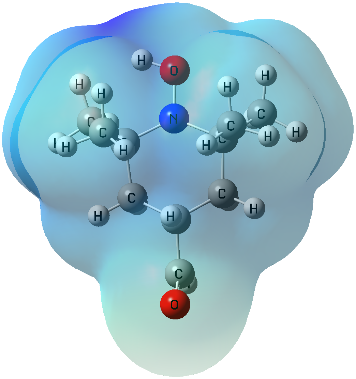** | **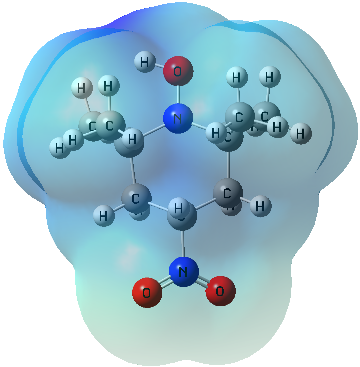** |

**Figure S2.** The shapes of the HOMO and LUMO orbitals calculated at the B3LYP (M06-2X) theoretical method for the **O-protonation** of the studied TEMPO derivatives.

| Optimized structure | X=H | X=CH_3_ | X=NH_2_ | X=CHO | X=NO_2_ |
| --- | --- | --- | --- | --- | --- |
| HOMO orbitals | **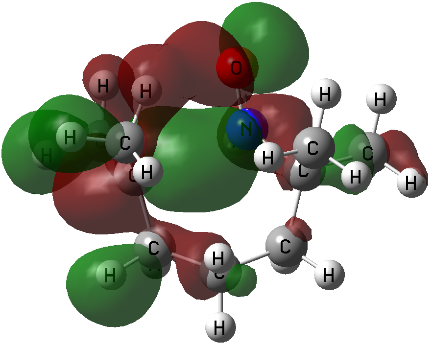**  *E* = −10.8111 (−14.6193) eV | **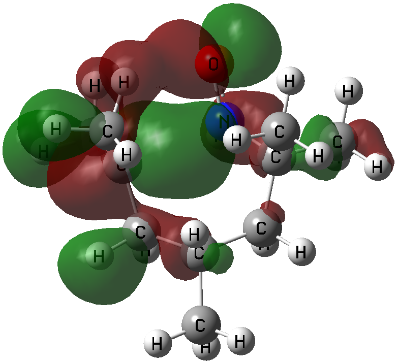**  *E* = −10.8600 (−14.2481) eV | **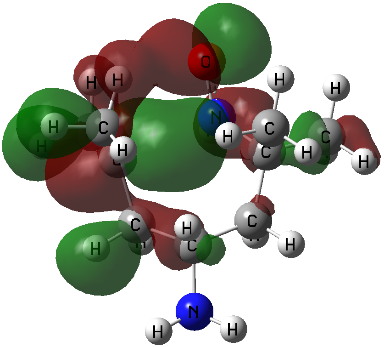**  *E* = −10.5117 (−12.1314) eV | **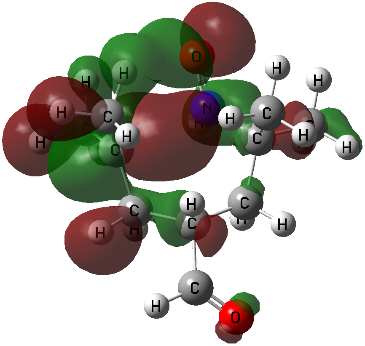**  *E* = −10.8628 (−12.610) eV | **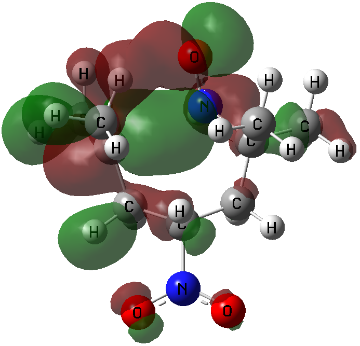**  *E* = −11.0668 (−13.902) eV |
| LUMO orbitals | **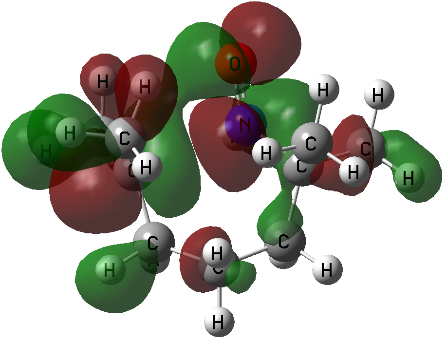**  *E* = −7.3416 (−3.6088) eV | **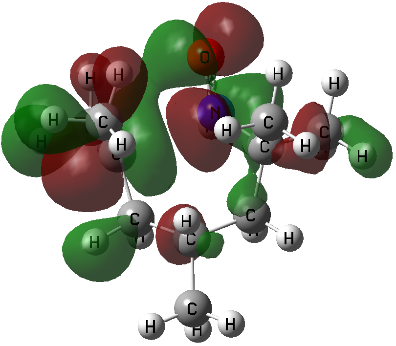**  *E* = −7.1811 (−3.5620) eV | **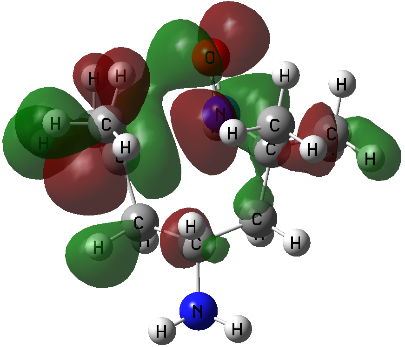**  *E* = −7.2219 (−3.5190) eV | **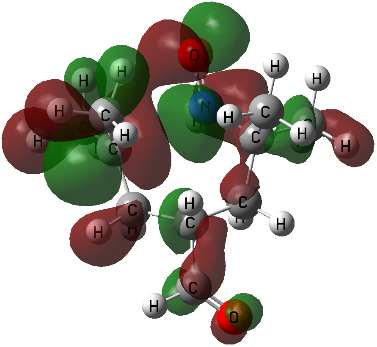**  *E* = −7.5865 (−3.6727) eV | **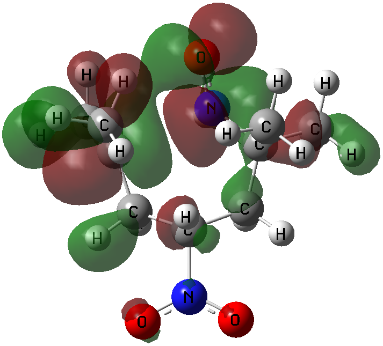**  *E* = −7.7960 (−4.1062) eV |
| MEP  maps | **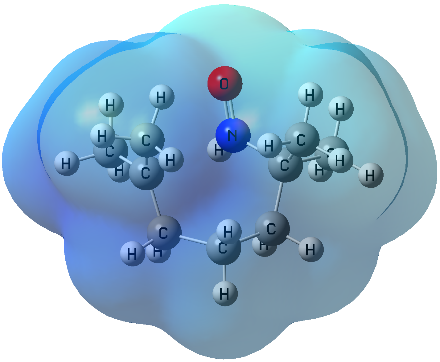** | **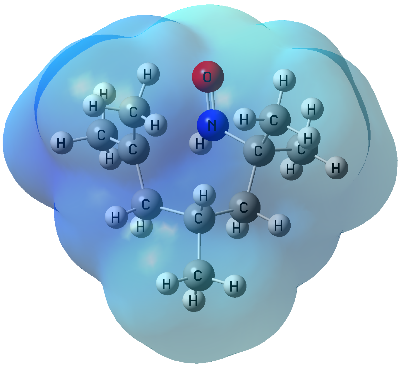** | **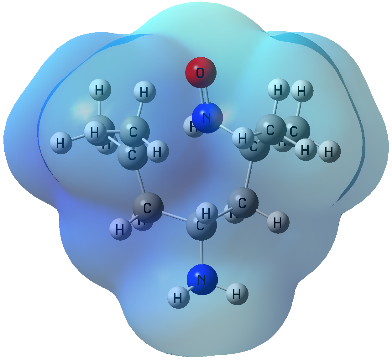** | **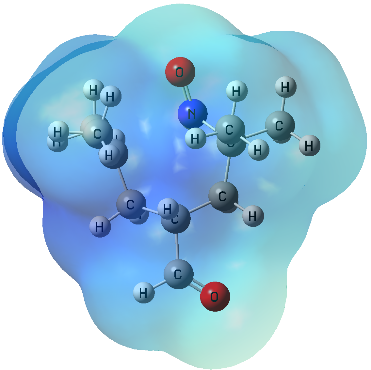** | **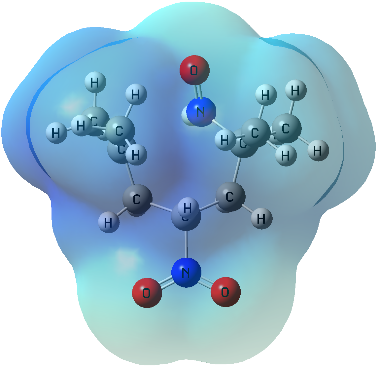** |

**Figure S3.** The shapes of the HOMO and LUMO orbitals calculated at the B3LYP (M06-2X) theoretical method for the **N-protonation** of the studied TEMPO derivatives.

| 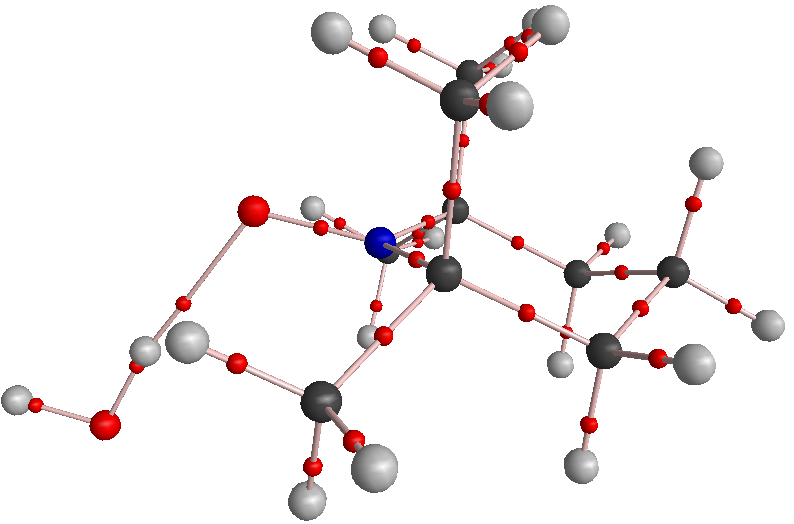  **BCP** | 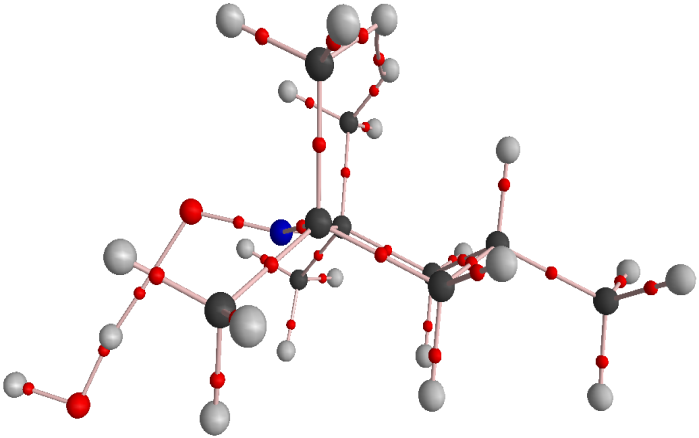  **BCP** |
| --- | --- |
| Tempo-H_2_O [*orthogonal*] | Tempo-CH_3_-H_2_O [*orthogonal*] |
| 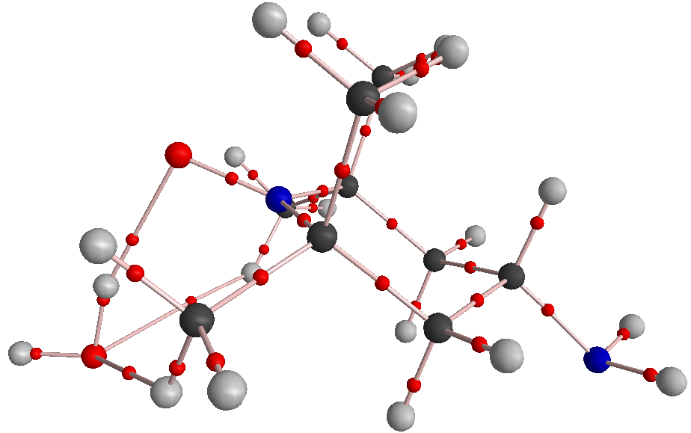  **BCP** | 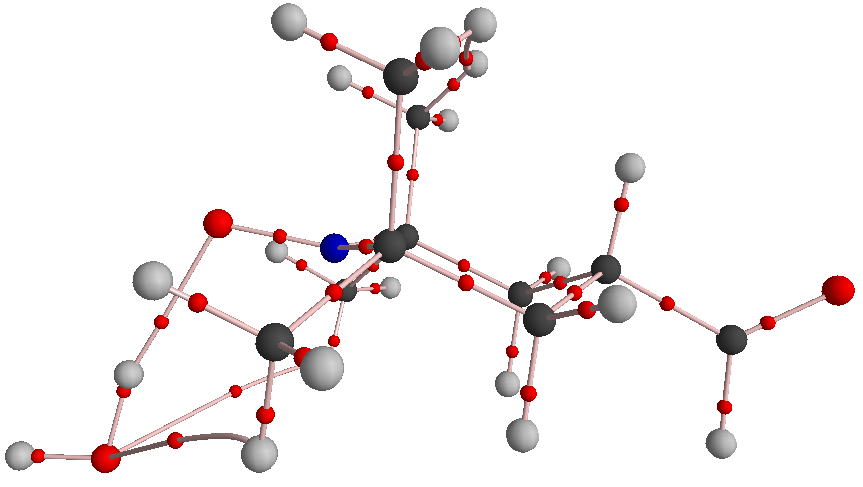  **BCP** |
| Tempo-NH_2_-H_2_O [*orthogonal*] | Tempo-CHO-H_2_O [*orthogonal*] |
| 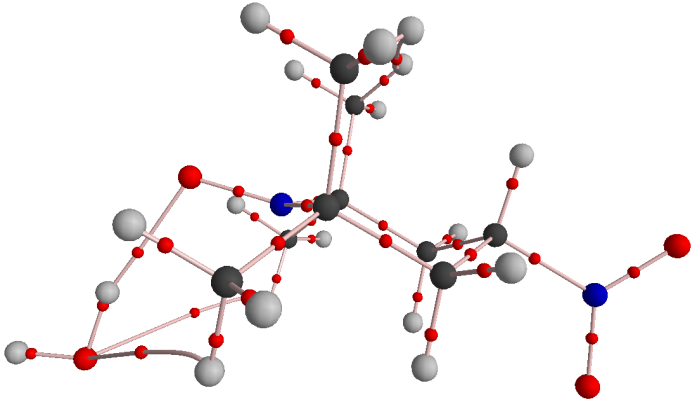  **BCP** | 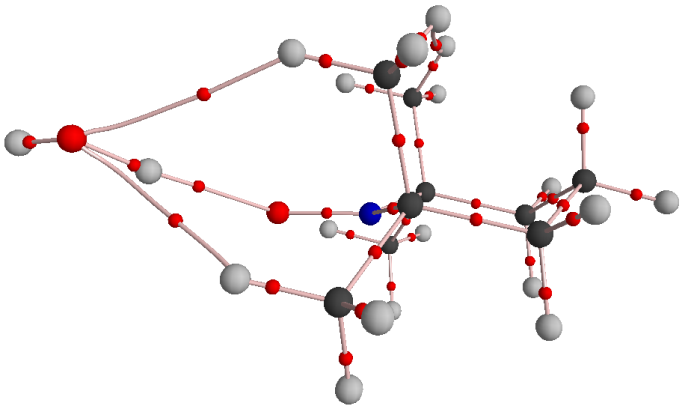  **BCP** |
| Tempo-NO_2_-H_2_O [*orthogonal*] | Tempo-H_2_O [*planer*] |
| 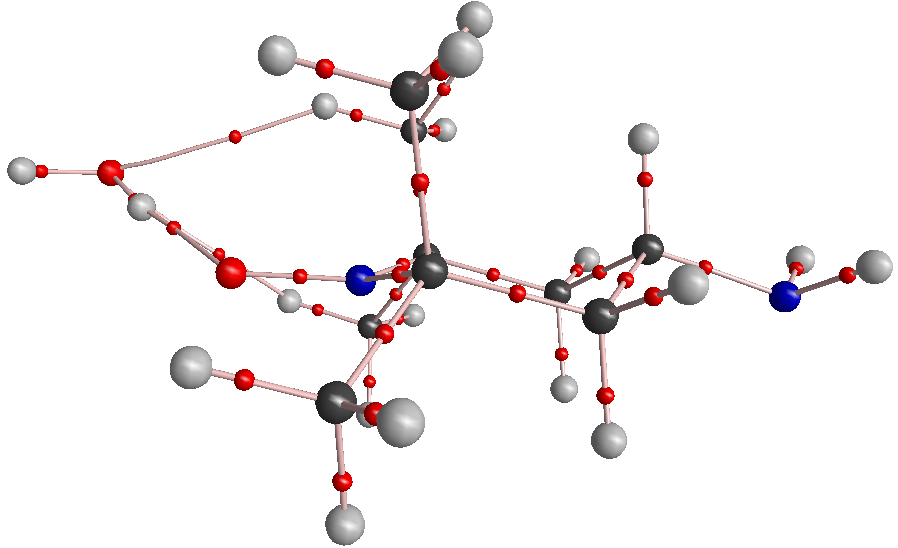  **BCP** | 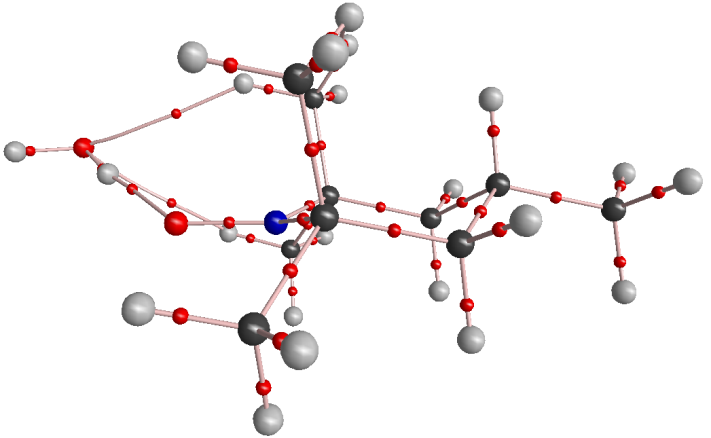  **BCP** |
| Tempo-NH_2_-H_2_O [*planer*] | Tempo-CH_3_-H_2_O [*planer*] |
| 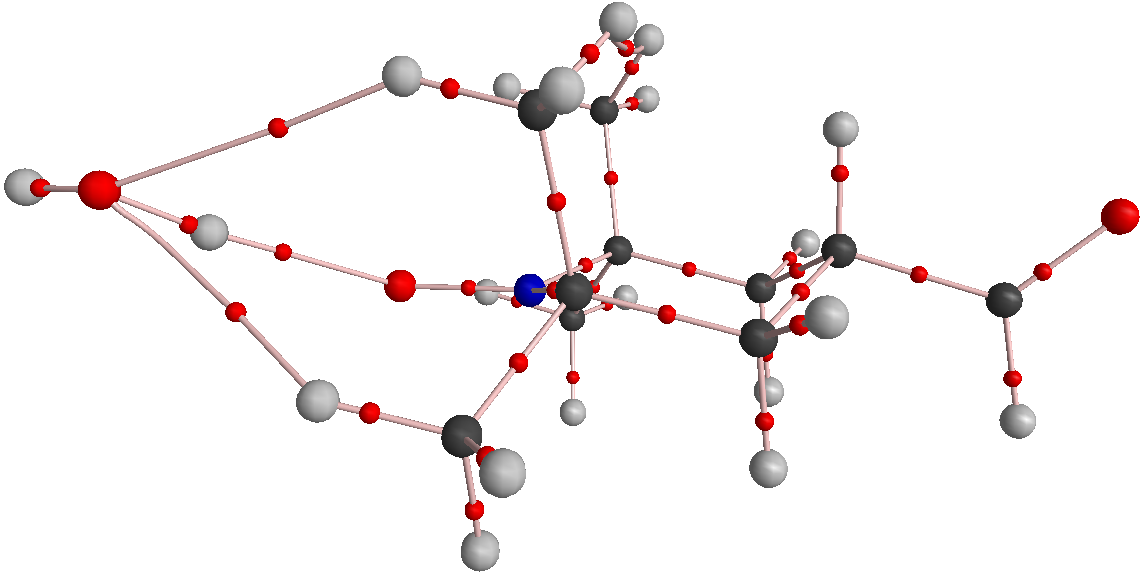  **BCP** | 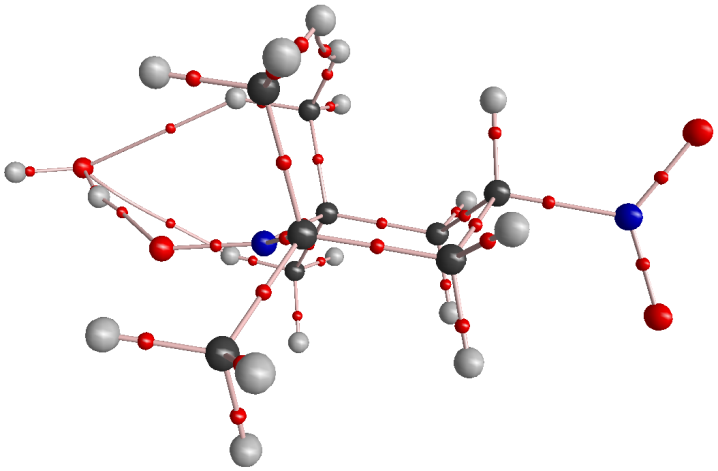  **BCP** |
| Tempo-CHO-H_2_O [*planer*] | Tempo-NO_2_-H_2_O [*planer*] |
| **BCP** | **BCP** |
| Tempo-H_2_O [*top*] | Tempo-CH_3_-H_2_O [*top*] |
| **BCP** | **BCP** |
| Tempo-NH_2_-H_2_O [*top*] | Tempo-CHO-H_2_O [*top*] |
| **BCP** | |
| Tempo-NO_2_-H_2_O [*top*] | |
| **Figure S4.** AIM features at the bond critical point of the TEMPO derivatives. The molecular graph was obtained at the B3LYP/6-311++G(d,p) level of theory. | |

1. ^🖂^ Corresponding authors:

   E-mails: abolfazl.shiroudi@pg.edu.pl (A. Shiroudi); Mohamed.Abdel-Rahman@sci.suezuni.edu.eg (M.A. Abdel-Rahman) [↑](#footnote-ref-1)
